# Immune-stromal heterogeneity in breast cancer across diverse ancestries: impact on prognosis and treatment response

**DOI:** 10.1038/s41523-025-00881-1

**Published:** 2025-12-12

**Authors:** Nanfizat A. Alamukii, Anikó Kovács, Sukanya Raghavan, Josefin Ilio, Per Karlsson, Khalil Helou, Toshima Z. Parris

**Affiliations:** 1 Department of Oncology, Institute of Clinical Sciences, Sahlgrenska Academy, University of Gothenburg, Gothenburg, Sweden; 2 Sahlgrenska Center for Cancer Research, Sahlgrenska Academy, University of Gothenburg, Gothenburg, Sweden; 3 Department of Clinical Pathology, Region Västra Götaland, Sahlgrenska University Hospital, Gothenburg, Sweden; 4 Department of Microbiology and Immunology, Institute of Biomedicine, Sahlgrenska Academy, University of Gothenburg, Gothenburg, Sweden; 5 Department of Clinical Immunology and Transfusion Medicine, Region Västra Götaland, Sahlgrenska University Hospital, Gothenburg, Sweden; 6 Department of Laboratory Medicine, Institute of Biomedicine, Sahlgrenska Academy, University of Gothenburg, Gothenburg, Sweden; 7 Department of Oncology, Region Västra Götaland, Sahlgrenska University Hospital, Gothenburg, Sweden

**Keywords:** Biomarkers, Cancer, Computational biology and bioinformatics, Immunology, Oncology

## Abstract

Breast cancer immune phenotypes influence treatment response and clinical outcomes, yet their ancestry-specific variations remain underexplored. Here, we analyzed transcriptomic data from over 13,000 breast tumors across six ancestry groups to characterize immune-stromal profiles and their association with ancestry, biological features, treatment response, and survival outcomes. Expression patterns were validated by spatial proteomics and immunohistochemistry. K-means clustering consistently identified three immune phenotypes (Hot, Moderate, or Cold) that varied significantly by ancestry, age, molecular subtype, and prognosis. Logistic regression and ancestry-associated analyses revealed that while immune phenotypes were primarily driven by PAM50 subtype, age, and disease stage, notable ancestry-related differences persisted, with European ancestry generally exhibiting higher immune and stromal activity across breast cancer subtypes. Hot tumors, enriched in the Basal-like and HER2 subtypes, were associated with younger age, higher immune infiltration, and improved overall survival. African ancestry was linked to elevated immune scores and upregulation of BTLA-mediated T cell co-inhibition, suggesting sensitivity to immunotherapy. European and East Asian tumors showed stromal enrichment, particularly inflammatory and myofibroblastic cancer-associated fibroblasts, associated with poor prognosis. Core immune activation genes (e.g., *CD3*, *CD2*, and *CXCL10*) were conserved, while ancestry-specific signatures and chemokine signaling were identified. This study uncovers both shared and ancestry-specific immunogenomic features of breast cancer, highlighting the role of ancestry and other biological features in shaping the tumor immune microenvironment. These findings re-emphasize the need for population-informed approaches in breast cancer immunotherapy and biomarker development, to ensure equitable precision oncology strategies across global populations.

## Introduction

The tumor microenvironment (TME) has been a central focus of cancer research for over a decade, driven by its critical role in tumor progression, therapeutic response, and disease outcomes. Understanding the TME is especially vital for improving treatment strategies across various cancer types, including breast cancer, a disease that remains a significant public health challenge worldwide, particularly in low- and middle-income countries^1^. Despite major advances in early detection and therapy, breast cancer continues to exhibit rising global incidence and mortality. In 2022, 2.3 million women were diagnosed with breast cancer globally, and approximately 670,000 deaths were recorded^2^. While mortality has declined in countries with very high Human Development Indices (HDI), these improvements are not universal^2^. However, some countries, such as Belgium and Denmark, have achieved the Global Breast Cancer Initiative goal of at least a 2.5% annual reduction. By 2050, breast cancer cases and deaths are projected to increase by 38% in regions with high-HDI and 68% in low-HDI, with the greatest burden expected in low-HDI regions^2^.

Although socioeconomic factors partially explain regional disparities in breast cancer outcomes, they do not fully account for the variation in disease progression, treatment response, and survival across populations^3^. Increasing evidence suggests that ancestral genetic differences contribute to tumor biology and immune landscape, influencing disease-free survival in breast cancer patients of diverse backgrounds^3^. Recent advances in immunotherapy have shown promising results in breast cancer treatment. For example, the inclusion of durvalumab in neoadjuvant chemotherapy regimens has significantly improved overall survival (OS) compared to chemotherapy alone in triple-negative breast cancer (TNBC)^4^. However, response to durvalumab varies by ancestry, with Asian patients exhibiting better OS compared to their Caucasian counterparts^5^. Identifying robust biomarkers that can predict response to such treatments remains an urgent clinical need^4,6^. Programmed death-ligand 1 (PD-L1) is currently the most established biomarker guiding immunotherapy in breast cancer, with multiple clinical trials confirming its predictive value^6,7^. High PD-L1 expression is generally associated with a favorable prognosis and increased sensitivity to chemotherapy^8^. Nonetheless, expanding the repertoire of predictive biomarkers is essential to optimize treatment selection and improve patient outcomes across populations.

The TME is defined by the presence of cancer cells, stromal cells, and the extracellular matrix. Based on the degree and pattern of T cell infiltration, tumors can be classified as immune-infiltrated-inflamed (hot), immune-excluded (moderate) or immune-desert (cold) tumors, collectively referred to as tumor immune phenotypes. The immune microenvironment in breast cancer is highly variable and subtype-specific. For instance, TNBC frequently exhibits high levels of CD8^+^ T cell infiltration, which is strongly associated with improved disease-free survival^9^. Moreover, subtle shifts in genetic ancestry are known to drive molecular differences even within PAM50 subtypes, potentially influencing disparities in tumor behavior and survival outcomes^3^. We hypothesize that ancestry-associated molecular differences significantly influence the composition and dynamics of the TME, thereby affecting breast cancer progression and patient survival. In this study, we assessed the clinical relevance of ancestry-specific variations in tumor immune phenotypes across diverse populations, with a focus on immune-stroma cell profiles, therapeutic response, and survival outcomes.

## Results

### Distribution of immune phenotypes in breast cancer patients based on ancestry, age, and PAM50 subtype

**Fig. 1:**
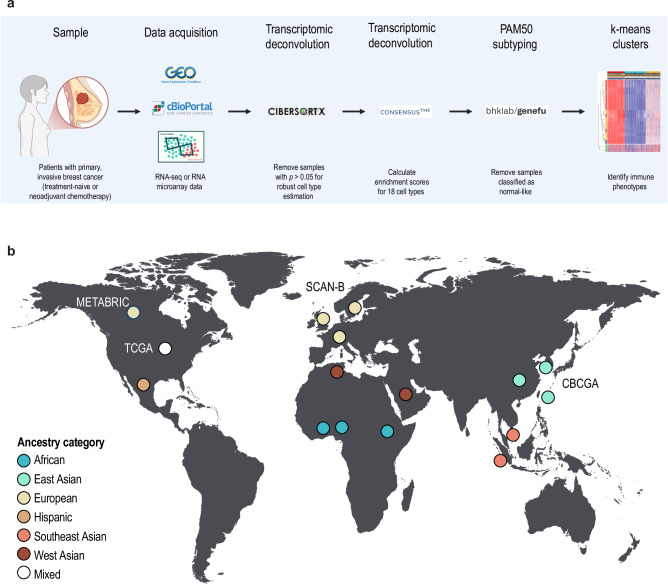
Overview of the workflow and study population. **a** The study design, including data collection and multiple analytical approaches used to characterize the immune phenotypes. **b** World map displaying the ancestral backgrounds of the compiled patient datasets included in the study, highlighting the global diversity of the cohort. Studies conducted in the USA (e.g., The Cancer Genome Atlas, TCGA) were classified into the six ancestry categories (African, East Asian, European, Hispanic, Southeast Asian, and West Asian) based on self-reported race listed in the study clinical data. Data from the USA were therefore of mixed ancestry. (Created in BioRender. Parris, T. (2025*)* https://BioRender.com/u29q104).

To evaluate ancestry-related differences in the proportion of immune and stroma cells in primary, invasive breast cancer, CIBERSORTx and ConsensusTME deconvolution were conducted using transcriptomic data for 13,731 patients (Fig. 1a). In total, transcriptomic and clinical data were compiled for 22 studies from North America, Europe, Africa, and Asia. Most patients were ≥50 years of age (69%) with tumors classified as T1/T2 (61%), grade 2/3 (55%), and Luminal A/B (72%) with no metastatic spread to the axillary lymph nodes (41%; Supplementary Table 1). The patients were then categorized into six ancestry groups (African, East Asian, European, Hispanic, Southeast Asian, and West Asian) based on self-reported race or the geographical region of the study’s country of origin (Fig. 1b). The cohort was primarily comprised of patients with European ancestry (83%), followed by East Asian ancestry (7.5%; Supplementary Table 1). The remaining four ancestry categories were comparatively uncommon (0.9–3.3%). As expected, the Luminal subtype was common among most ancestral groups, while African ancestry was primarily associated with the Basal-like and Luminal B subtypes.

**Fig. 2:**
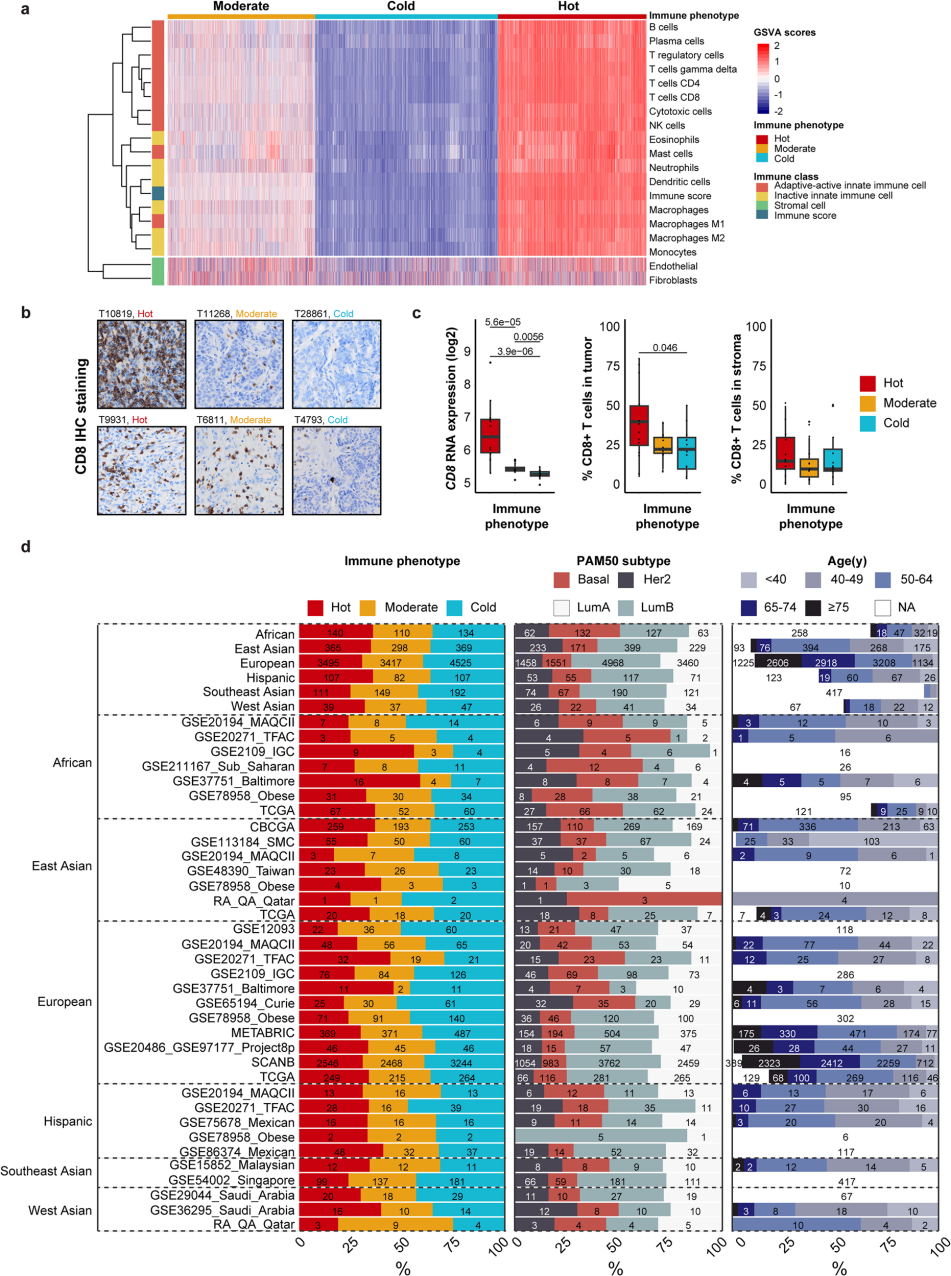
A comparative analysis of the immune phenotypes, PAM50 molecular subtypes, and age distribution across different ancestry groups in patients with breast cancer. **a** Heatmap for the 22 datasets showing samples classified into the three immune phenotypes (Hot, Moderate, Cold). **b** Representative CD8 immunostaining for two samples from each immune phenotype (Hot, Moderate, and Cold) are shown. Images were scanned at ×20 magnification. **c** *CD8A* RNA expression differences across immune phenotypes and CD8+ cell infiltration in tumor and stroma across immune phenotypes (*p* > 0.05). **d** Patients and their tumor specimens were classified by immune phenotype (Hot, Moderate, Cold), PAM50 subtyping (Basal, Luminal A [LumA], Luminal B [LumB], and Her2), and age groups (<40, 40–49, 50–64, 65–74, ≥75 years); the numbers in the stacked bar plot depict the number of patients. Cold tumors were the most prevalent across all ancestries, making up 39% of cases. Southeast Asians had the highest proportion of cold tumors (43%), while Hot phenotypes were observed at similar frequencies in African and East Asian groups (both around 36%). The moderate immune phenotype was relatively evenly distributed among all ancestry groups. LumB was the most frequently observed subtype (43%), followed by LumA (29%) and Basal (15%). The aggressive Basal subtype was prevalent among patients of African ancestry (34%), while East Asians showed an enrichment of the Her2 subtype (23%) and Europeans exhibited a higher frequency of LumA (30%) and LumB (43%) samples. Patients of European ancestry were primarily aged >50 years, whereas East Asians had the highest representation among younger patients aged <50 years. GSVA gene set variation analysis.

Classification of the immune phenotypes was conducted in a two-step process using (1) the online CIBERSORTx tool to first identify 22 immune cell fractions and remove samples with unreliable immune cell estimations (*p* > 0.05), followed by (2) the ConsensusTME workflow to calculate enrichment scores for 18 cell types (16 immune cells and two stromal cells). Using k-means clustering of the immune scores, all datasets were consistently classified into three immune phenotype groups (Fig. 2a): Hot (immune-infiltrated; 31%), Moderate (immune-excluded; 30%), and Cold (immune-desert; 39%). Using selected patient samples from the GSE97177 and GSE20486 cohorts, immunohistochemistry was performed for CD8 to validate the three immune phenotypes among breast cancer patients (Fig. 2b). The Hot immune phenotype showed a significantly higher proportion of CD8+ cell infiltration in the tumor compared to the Moderate and Cold immune phenotypes. Similar *CD8A* expression patterns were observed across immune phenotypes (*p* < 0.05). In contrast, no significant differences were found in CD8+ cell infiltration within the stromal compartment (Fig. 2c). Tumor-infiltrating lymphocyte (TIL) assessment on hematoxylin and eosin–stained slides also showed that tumors with a Hot immune phenotype had a higher percentage of TILs compared to cold and moderate tumors. Across phenotypes, the proportion of TILs with intermediate CD8 expression (10–49%) was relatively higher (Supplementary Fig. S1).

The overall immune scores were significantly higher in tumors classified as Hot (mean 0.49, SD 0.14) compared to Moderate (mean −0.01, SD 0.17) and Cold (mean -0.45, SD 0.12; *p* < 0.001; Supplementary Table 2). Despite a relatively even distribution of the three immune phenotypes within each ancestry group, Hot tumors were more common in the African, East Asian, and Hispanic populations, while Cold tumors were more prevalent among Europeans, Southeast Asians, and West Asians (Fig. 2c). Intriguingly, the highest proportion of Hot tumors was found in three datasets (GSE2109 African, GSE37751 African and European, and GSE20271 European). Molecular subtype distribution (based on PAM50 classification) and patient age were also significantly associated with immune phenotype (*p* < 0.001; Supplementary Table 2). Hot tumors were predominantly comprised of Luminal B (33%) and Basal-like (27%) tumors, while both Moderate and Cold tumors were primarily classified as the Luminal subtype (73-86%). Basal-like tumors were predominantly found in the Hot group (27%), compared to 12% in Moderate and 6% in Cold tumors. Her2-enriched tumors showed similar patterns, with 22% in the Hot group, 14% in Moderate, and 7% in Cold tumors. Intriguingly, patients >75 years old represented 19% of the total cohort, with more patients in this age group being classified as Cold (21%), while individuals aged 50–64 years and <40 years were more frequently found in the Hot group. No significant differences in immune phenotype distribution were found for patients aged 65–74 years.

**Fig. 3:**
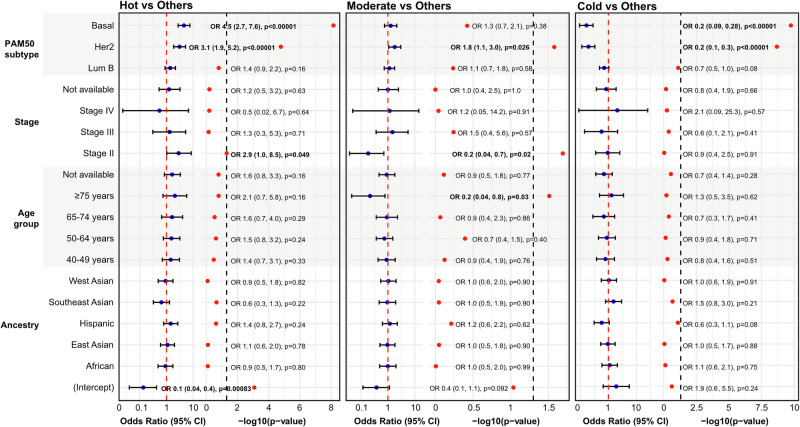
Association between ancestry, age, tumor stage, and PAM50 subtypes with immune phenotypes with a matched balanced sampled size. Forest plots displaying logistic regression between the immune phenotypes and ancestry, age, tumor stage, and PAM50 subtype. Basal-like and Her2-enriched (PAM50 subtypes) were significantly associated with the Hot immune phenotype (Odds ratio [OR] > 1, *p* < 0.05), while Her2-enriched, stage II breast cancer, and age (≥75 years) were significantly associated with the Moderate immune phenotype (OR > 1, *p* < 0.05), and Basal-like and Her2-enriched (PAM50 subtypes) were significantly associated with the Cold immune phenotype (OR > 1, *p* < 0.05). References for each category are Ancestry European, age <40, stage I, and subtype LumA.

Using cardinality matching to balance sample sizes across all features, logistic regression analysis was then performed to identify key predictors (e.g., ancestry, age group, tumor stage, and PAM50 subtype) of immune phenotypes in breast cancer (Fig. 3). By comparing each immune phenotype to the others (Hot vs others, Moderate vs others, and Cold vs others), this analysis revealed that Basal-like and Her2-enriched (PAM50 subtypes) were significantly associated with the Hot immune phenotype (Odds ratio [OR] > 1, *p* < 0.05), while Her2-enriched, stage II breast cancer, and age (≥75 years) were significantly associated with the Moderate immune phenotype (OR > 1, *p* < 0.05) and Basal-like/Her2-enriched (PAM50 subtypes) were significantly associated with the Cold immune phenotype (OR > 1, *p* < 0.05), indicating that breast cancer molecular subtype, age, and tumor stage rather than ancestry are the primary determinants of immune phenotype differences across patients.

### Immune and stromal cell profiling across ancestries and PAM50 subtypes

**Fig. 4:**
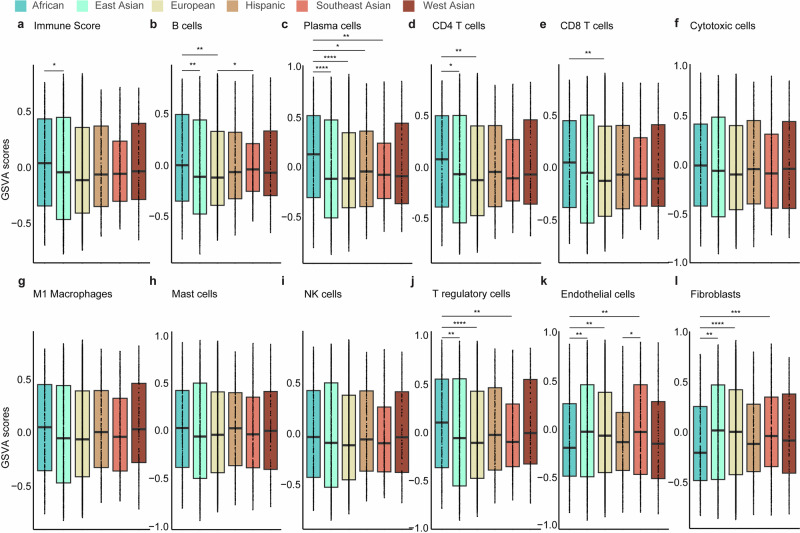
Variation in immune and stroma cell composition and gene set variation analysis (GSVA) scores across the six ancestries (African, East Asian, European, Hispanic, Southeast Asian, and West Asian). Box plots display the **a** overall immune score and GSVA scores for **b** B cells, **c** plasma cells, **d** CD4 + T cells, **e** CD8 + T cells, **f** cytotoxic cells, **g** M1 macrophages, **h** mast cells, **i** NK cells, **j** T regulatory cells, **k** endothelial cells, and **l** fibroblasts. The Wilcoxon test was used to calculate statistically significant differences (Benjamini–Hochberg adjusted *p* values) between the ancestry groups. Not significant (*p-adj* > 0.05); *p-adj < 0.05; **p-adj ≤ 0.01; ***p-adj ≤ 0.001; ****p-adj ≤ 0.0001. Only statistically significant differences are shown in the figure.

Using the ConsensusTME-derived gene set variation analysis (GSVA) enrichment scores, we then assessed immune and stromal cell profiles across the different ancestries and PAM50 subtypes. The African population consistently exhibited higher immune GSVA scores, particularly for adaptive and active innate immune cells, including B cells, CD4+ and CD8 + T cells, M1 macrophages, plasma cells, and regulatory T cells, compared to the other groups (Fig. 4). Notably, immune scores were significantly elevated in the African group relative to the East Asian population. Similarly, the African and Southeast Asian populations showed significantly higher GSVA scores for B cells, CD4 + T cells, and CD8 + T cells compared to the East Asian, European, and Hispanic groups. Plasma cell scores were also markedly higher in the African population compared to European, East Asian, Hispanic, and Southeast Asian populations. Additionally, regulatory T cell scores were significantly elevated in the African and West Asian populations compared to European and Hispanic groups. Conversely, GSVA scores for stromal cells, including endothelial cells and fibroblasts, were significantly lower in the African and Hispanic populations compared to East Asian, European, and Southeast Asian groups. No significant differences in GSVA scores were observed for cytotoxic cells, M1 macrophages, mast cells, or NK cells across the ancestral groups.

For the PAM50 subtypes, Basal-like and Her2-enriched tumors exhibited the highest immune scores across all profiled immune cells, whereas the Luminal A and Luminal B subtypes had lower immune infiltration. Significant differences were also noted in GSVA scores for stromal cells. Specifically, the Basal-like subtype had significantly lower endothelial cell scores than Her2-enriched and Luminal A tumors and lower fibroblast scores compared to all other subtypes. In contrast, Luminal A displayed the highest GSVA scores for stromal cells. The Her2-enriched subtype exhibited higher stromal scores than Luminal B and Basal-like tumors but significantly lower scores than Luminal A (Supplementary Fig. S3).

**Fig. 5:**
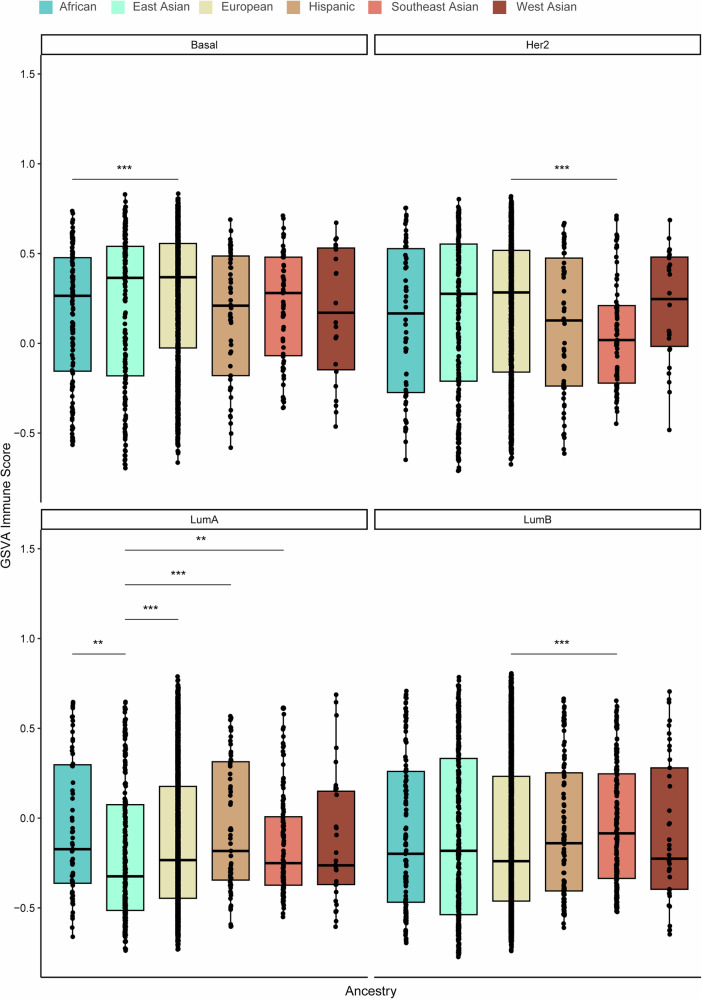
Variation in gene set variation analysis (GSVA) immune scores among PAM50 breast cancer subtypes: Basal-like (Basal), HER2-enriched (Her2), Luminal A (LumA) and Luminal B (Lum B) between ancestries. The Wilcoxon test was used to calculate statistically significant differences (Benjamini–Hochberg adjusted *p* values) between the ancestries. Not significant (*p* > 0.05); *p < 0.05; **p ≤ 0.01; ***p ≤ 0.001; ****p ≤ 0.0001. Only statistically significant differences are shown in the figure.

**Fig. 6:**
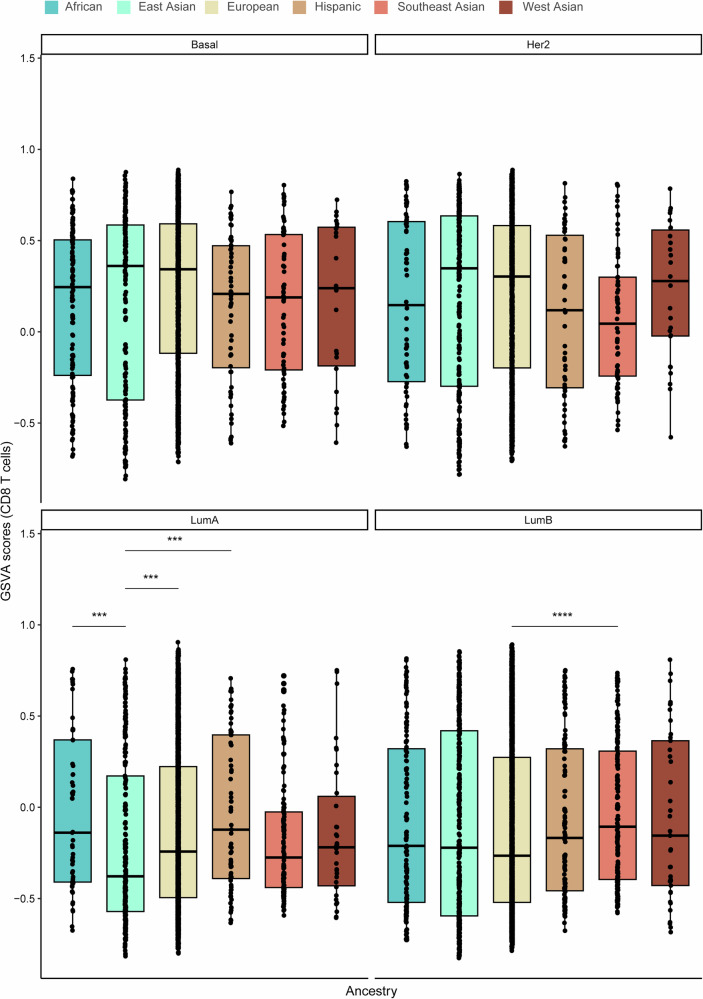
Variation in gene set variation analysis (GSVA) CD8 T cell scores among PAM50 breast cancer subtypes: Basal-like (Basal), HER2-enriched (Her2), Luminal A (LumA) and Luminal B (Lum B) between ancestries. The Wilcoxon test was used to calculate statistically significant differences (Benjamini–Hochberg adjusted *p* values) between the ancestries. Not significant (*p* > 0.05); *p < 0.05; **p ≤ 0.01; ***p ≤ 0.001; ****p ≤ 0.0001. Only statistically significant differences are shown in the figure.

**Fig. 7:**
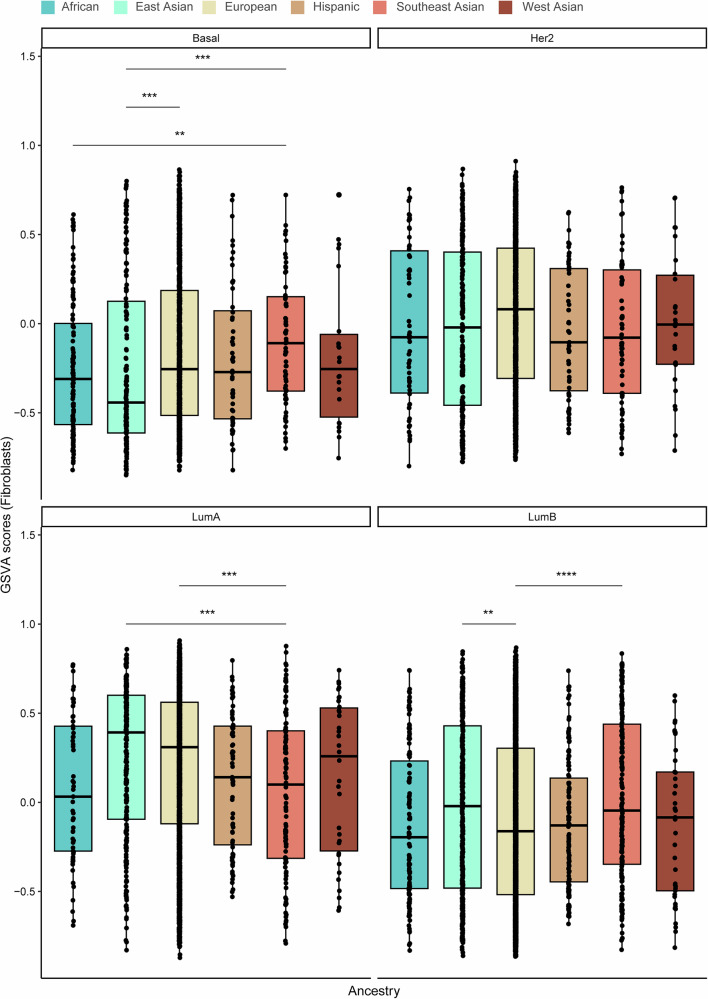
Variation in gene set variation analysis (GSVA) fibroblasts scores among PAM50 breast cancer subtypes: Basal-like (Basal), HER2-enriched (Her2), Luminal A (LumA) and Luminal B (Lum B) between ancestries. The Wilcoxon test was used to calculate statistically significant differences (Benjamini–Hochberg adjusted *p* values) between the ancestries. Not significant (*p* > 0.05); *p < 0.05; **p ≤ 0.01; ***p ≤ 0.001; ****p ≤ 0.0001. Only statistically significant differences are shown in the figure.

**Fig. 8:**
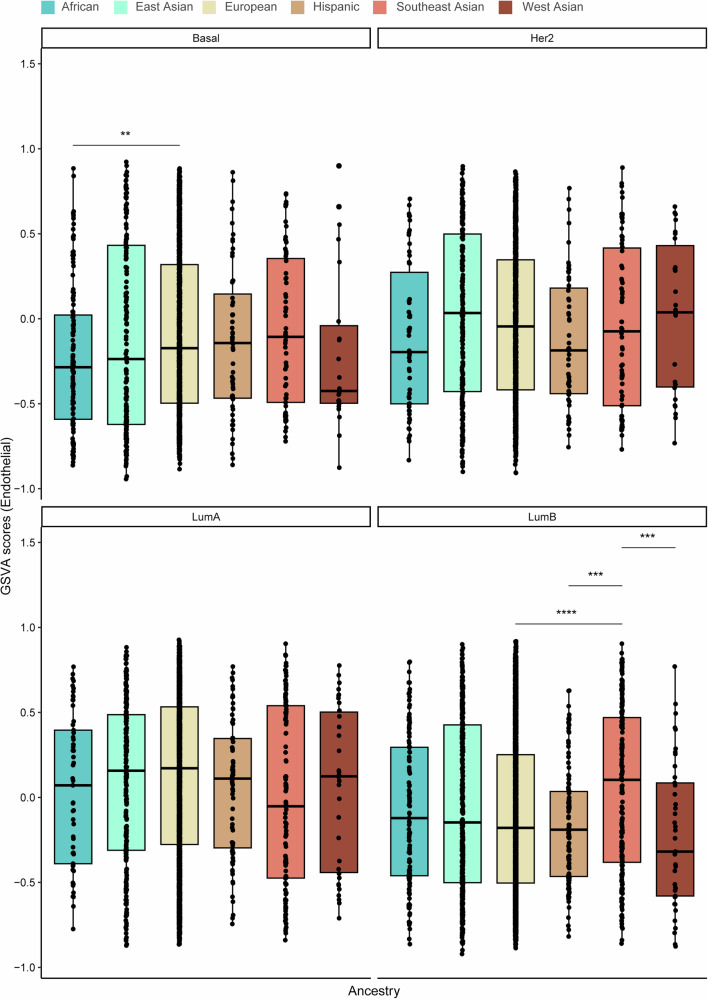
Variation in gene set variation analysis (GSVA) endothelial scores among PAM50 breast cancer subtypes: Basal-like (Basal), HER2-enriched (Her2), Luminal A (LumA) and Luminal B (Lum B) between ancestries. The Wilcoxon test was used to calculate statistically significant differences (Benjamini–Hochberg adjusted *p* values) between the ancestries. Not significant (*p* > 0.05); *p < 0.05; **p ≤ 0.01; ***p ≤ 0.001; ****p ≤ 0.0001. Only statistically significant differences are shown in the figure.

To further delineate ancestry-associated immune and stromal heterogeneity within molecular subtypes, we compared GSVA-derived immune, CD8^+^ T-cell, endothelial, and fibroblast scores across PAM50 breast cancer subtypes **(**Figs. 5–8**)**. Distinct ancestry-related differences were observed across multiple subtypes. Notably, within the Basal-like subtype, individuals of European ancestry exhibited significantly higher immune and endothelial scores compared to those of African ancestry. In the Her2-enriched subtype, immune and endothelial scores were also elevated in European relative to Southeast Asian ancestry. For Luminal A tumors, significant ancestry-associated variation was observed across all four phenotypes, with European and African ancestries displaying higher immune, CD8^+^ T-cell, and fibroblast scores compared to East Asian, Hispanic, and Southeast Asian groups. In the Luminal B subtype, European ancestry demonstrated significantly higher immune, CD8^+^ T-cell, endothelial, and fibroblast scores relative to Southeast Asian ancestry. Together, these findings highlight pronounced ancestry-associated differences in both immune and stromal cell activity within specific PAM50 subtypes of breast cancer.

**Fig. 9:**
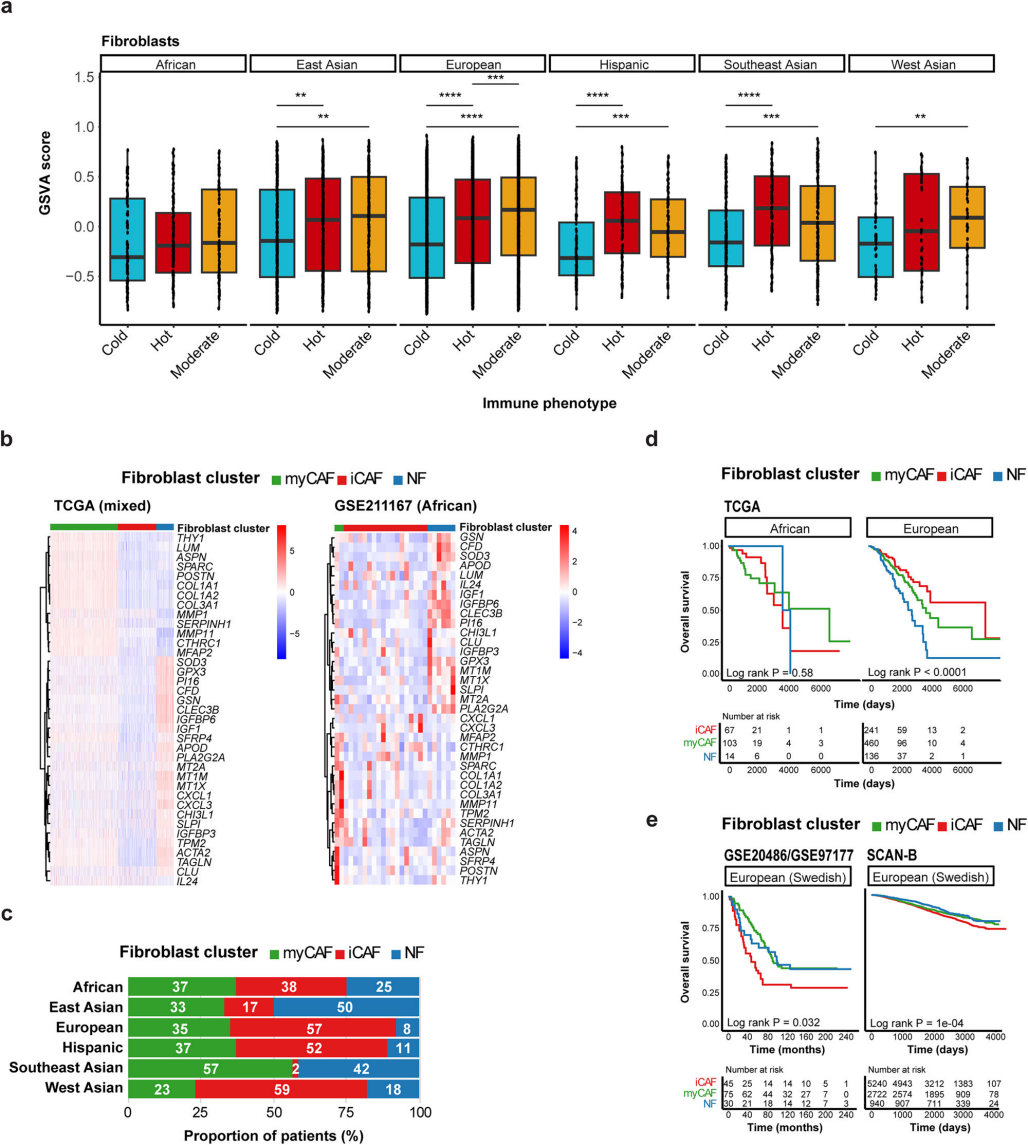
Fibroblast clusters are associated with immune phenotypes, ancestry, and survival outcomes in breast cancer. **a** Box plots display GSVA scores for fibroblasts based on immune phenotype and ancestry. The Wilcoxon test was used to calculate statistically significant differences (Benjamini–Hochberg adjusted p-values) between the immune phenotype groups. Not significant (*p-adj* > 0.05) and significant (*p-adj < 0.05; **p-adj ≤ 0.01; ***p-adj ≤ 0.001; ****p-adj ≤ 0.0001). Only statistically significant differences are shown in the figure. **b** Heatmaps showing gene expression patterns across fibroblast subtypes (myCAF, iCAF, NF) in TCGA (mixed ancestry) and GSE211167 (African cohort). **c** Distribution of fibroblast clusters (myCAF: green, iCAF: red, NF: blue) across different ancestries, indicating variability in the prevalence of the fibroblast subtypes. **d** Kaplan–Meier survival curves for African and European patients from TCGA, stratified by fibroblast subtype. iCAF enrichment is associated with worse survival in Europeans but not in Africans. **e** Validation of survival association with fibroblast subtypes in two independent European cohorts from Sweden (GSE20486/GSE97177 and SCAN-B). The iCAF subtype consistently shows poorer overall survival.

Given the significantly lower GSVA scores for fibroblasts in the African population, we then investigated the relationship between the fibroblast scores, immune phenotype, and ancestry. We were thereby able to show consistently higher fibroblast scores in hot/moderate vs cold tumors in all ancestry groups except the African population (Fig. 9a). To explore this further, we used 37 markers to classify fibroblasts into normal fibroblasts (NF) and cancer-associated fibroblasts (CAFs; myofibroblast-like CAFs [myCAFs] and inflammatory CAFs [iCAFs]). Across the different ancestries, gene expression profiling revealed distinct transcriptional signatures corresponding to the three fibroblast subtypes. These subtype-specific expression patterns were evident in both the TCGA mixed-ancestry cohort and an independent African cohort (GSE211167; Fig. 9b). When stratified by ancestry, the prevalence of fibroblast subtypes varied markedly. For example, African and Southeast Asian patients displayed a more balanced distribution of the fibroblast subtypes, whereas European, West Asian, and Hispanic patients had a higher proportion of iCAFs, and East Asian patients showed a relative enrichment of NFs (Fig. 9c and Supplementary Table 3).

Survival analyses indicated that fibroblast composition has prognostic value. In the TCGA dataset, the presence of NFs was associated with significantly worse OS in European patients, while no significant differences in survival were observed in African patients (Fig. 9d). However, two independent European cohorts from Sweden (GSE20486/GSE97177 and SCAN-B) revealed that patients with iCAF-dominant tumors consistently exhibited worse survival (Fig. 9e). Together, these results suggest that fibroblast heterogeneity is shaped by immune context and ancestry, and that specific fibroblast subtypes, particularly iCAFs, may contribute to adverse clinical outcomes in breast cancer.

### Gene signatures linked to the hot immune phenotype

**Fig. 10:**
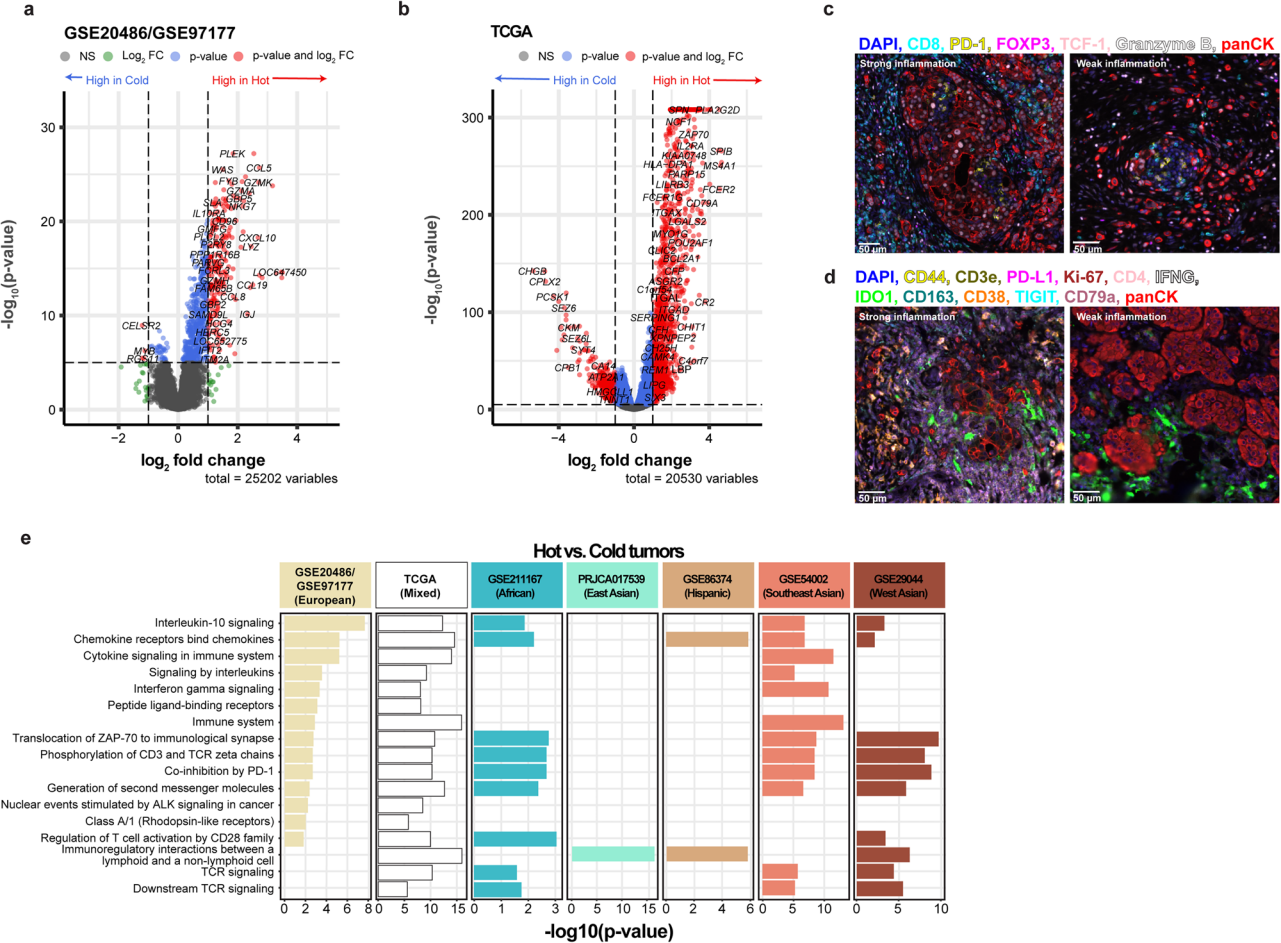
Differential gene expression and pathway analyses for the hot vs cold immune phenotypes in representative ancestries. Using the R package DESeq2, differentially regulated genes were identified and visualized using the Enhanced Volcano package, where the *x*-axis represents the log₂ fold change and the *y*-axis corresponds to the –log₁₀ *p* value. Differentially regulated genes (log₂ fold change > |2| and *p*-adj<0.05) are highlighted in red. Differentially expressed genes in the **a** GSE20486/GSE97177 dataset and **b** TCGA dataset. Multiplex immunofluorescence spatial proteomics images of two breast cancer patient samples exhibiting strong and weak inflammation, stained for immune markers **c** CD8, FOXP3, GZMB, PD-1, pan-cytokeratin, and nuclear TCF-1, as well as **d** CD38, CD3ε, CD4, CD44, CD79A, CD163, IDO1, IFN-γ, Ki-67, PD-L1, pan-cytokeratin, and TIGIT. **e** Pathway analyses of significantly up- and downregulated genes (log2fold change > |2|, *p* < 0.00001) in each representative dataset were performed using the online platform, Reactome. The top significant pathways (identified in at least two ancestral populations) associated to the differentially expressed genes in the Hot vs Cold immune phenotype in GSE20486/GSE97177 (European), TCGA (mixed), GSE211167 (African), PRJCA017539 (East Asian), GSE86374 (Hispanic), GSE54002 (Southeast Asian), and GSE29044 (West Asian) datasets.

To identify gene signatures linked to the hot immune phenotype compared to the cold immune phenotype, differential gene expression (DGE) and pathway analyses were performed using representative datasets from diverse ancestries, including African (GSE211167), East Asian (PRJCA017539), European (GSE20486/GSE97177), Hispanic (GSE86374), Southeast Asian (GSE54002), West Asian (GSE29044), and Mixed (TCGA dataset). DGE analysis revealed distinct transcriptomic patterns characterized by gene deregulation (log2 fold change > |2|, *p-adj* < 0.05). Differentially expressed genes were predominantly upregulated in Hot tumors across the datasets (African: 14 UP, 18 DOWN; East Asian: 857 UP, 83 DOWN; European: 25 UP, 0 DOWN; Hispanic: 20 UP, 0 DOWN; Southeast Asian: 77 UP, 8 DOWN; West Asian: 58 UP, 0 DOWN; Mixed: 342 UP, 49 DOWN; Fig. 10a, b). Notably, no downregulated genes were identified in the European (GSE20486/GSE97177), Hispanic (GSE86374), and West Asian (GSE29044) datasets. Key immune activation and tumor suppression genes including *CD2, CD3, CD247 (*CD3 zeta chain*), CD48, CD84, CCL19, CXCL10*, and *SLAMF6* were consistently upregulated in the hot phenotype across all ancestries (Fig. 10). Genes encoding cytotoxic granules characteristic of T cells and natural killer (NK) cells, such as *GZMA, GZMB*, and *GZMK*, were upregulated in all ancestries except African and Hispanic. The macrophage M2 marker *CD163* was specifically upregulated in the Southeast Asian and Mixed (TCGA) groups. To validate these results, spatial proteomics was performed for 17 markers (CD38, CD3ε, CD4, CD8, CD44, CD79A, CD163, FOXP3, GZMB, IDO1, IFN-γ, Ki-67, PD-1, PD-L1, pan-cytokeratin, TCF-1, and TIGIT) with breast cancer samples exhibiting strong and weak inflammation (Fig. 10c, d). All markers were visibly expressed in both inflammation states, with higher expression generally observed in samples with strong inflammation. Notably, Granzyme B showed weak expression in both conditions.

Pathway analysis of the deregulated genes revealed significant enrichment in immune signaling pathways, including phosphorylation of CD3 and TCR zeta chains (*CD3D, CD247, HLA-DQA1*, and *TRAC*), T cell activation via the CD28 family (*CD3D, CD247, HLA-DQA1*, and *TRAC*), immune co-inhibition through PD-1 (*CD3D, CD247, HLA-DQA1*, and *TRAC*), chemokines receptor binding chemokines (*CXCL10, CXCL9, CCL5*, and *CCL19*), and Interleukin-10 signaling (*CCL5, CCL19*, and *CXCL10*; Fig. 10e). Notably, the BTLA-mediated co-inhibition of T cell responses (*BTLA*) was exclusive to the African ancestry group. In contrast, members of the TNF receptor superfamily (TNFSF), which mediate the noncanonical NF-κB pathway, a potential tumor-promoting mechanism (*NCR3, CD40LG, LTA, LTB*, and *TNFRSF13C*), were observed in East Asian and Mixed ancestry populations (TCGA). A separate tumor-promoting pathway involving ALK-stimulated nuclear events (*GZMB*) in cancer was only found in European and Mixed ancestry. Other pathways included regulation of gene expression in endocrine-committed (NEUROG3+) progenitor cells (*INSM1*) in Mixed ancestry and multiple estrogen-related signaling pathways in Southeast Asian ancestry, including Constitutive Signaling by Aberrant PI3K in Cancer (*ESR1*), extra-nuclear estrogen signaling (*ESR1*), TFAP2 (AP-2)-mediated transcriptional regulation of growth factors (*ESR1*), and ESR-mediated signaling (*TFF1, ESR1*). Although the East Asian and Hispanic cohorts shared few pathways with other ancestries, they both exhibited an enrichment of pathways related to Leishmania parasitic infections and immune responses, including Leishmania phagocytosis, FCGR3A−mediated phagocytosis, Parasite infection and FCGR3A−mediated IL10 synthesis (*IGHV4-59, IGKV3D-20*, and *IGKV1D-33*; Fig. 10e and Supplementary Fig. S3).

### Prognostic significance of immune phenotype and immune-stromal scores across ancestries

**Fig. 11:**
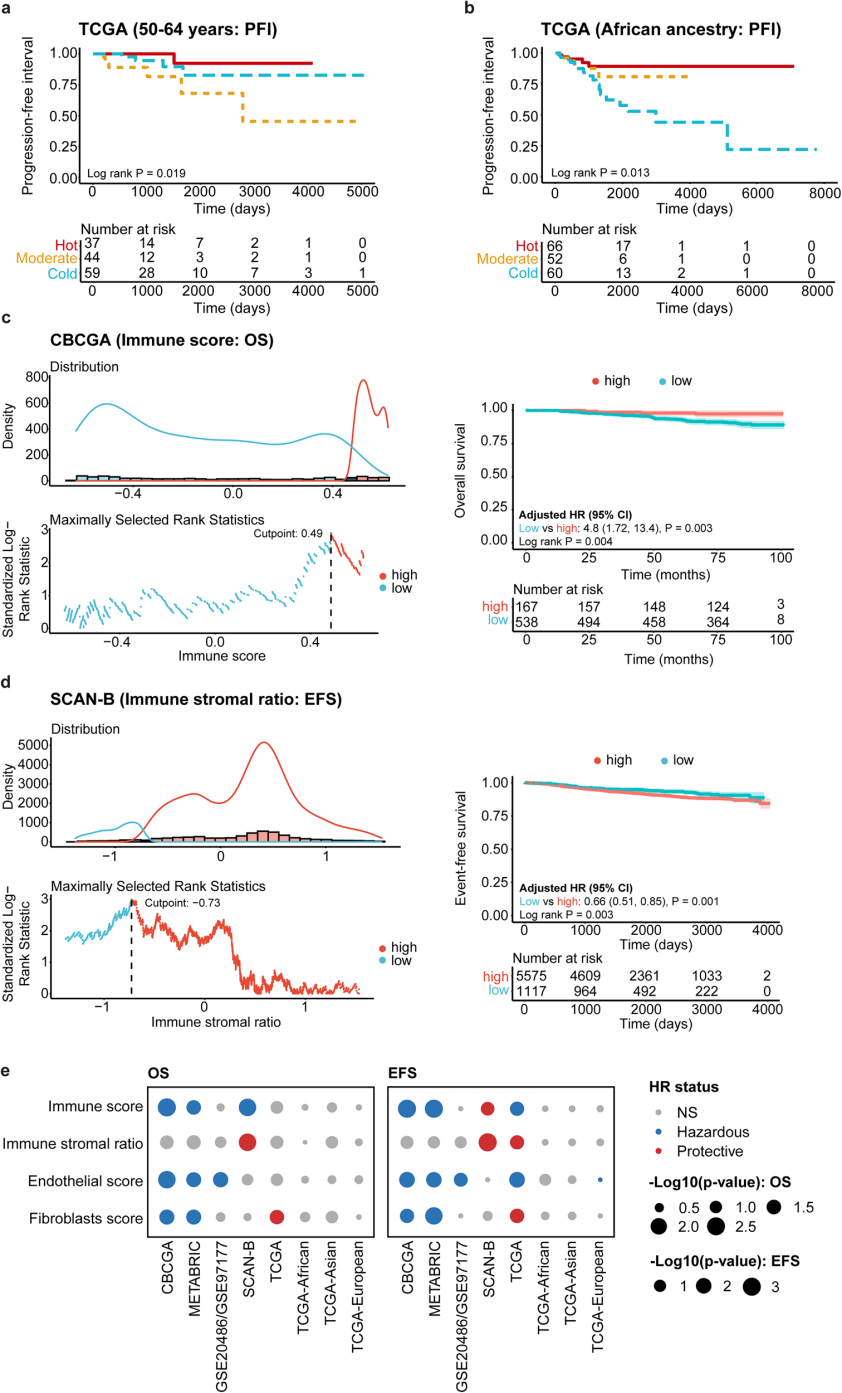
Survival analysis of progression-free interval (PFI), overall survival (OS), and event-free survival (EFS) across immune phenotypes, as well as immune and stromal cell scores, in selected datasets (CBCGA, METABRIC, GSE20486/GSE97177, SCAN-B, and TCGA). These analyses were performed using the survminer (version 0.4.9), survival (version 3.4-0), and gtsummary (version 1.7.2) packages to determine the optimal cutpoint for the variables. Survival analysis for PFI between the immune phenotypes (Hot, Moderate, and Cold) in the TCGA dataset for patients **a** aged 50-64 years and **b** of African ancestry. **c** The cutpoint (left) and Kaplan–Meier survival analysis (right) for the association between high and low immune scores and OS in the CBCGA dataset. Multivariable Cox regression models were adjusted using patient age at surgery and tumor grade. Lower immune score was significantly associated with worse OS (*p* < 0.05). **d** The cutpoint (left) and Kaplan–Meier survival analysis (right) for the association between high and low immune stromal ratios (calculated using the immune score/[endothelial score + fibroblasts score]) and OS in the SCAN-B dataset. High immune stromal ratios were associated with worse EFS (*p* < 0.05). The models were adjusted using patient age and Nottingham histological grade. **e** Dot plots displaying the −log10(*p* value) for the multivariable Cox regression analysis between immune scores, immune stromal ratio, endothelial scores, and fibroblasts scores and OS (left) and EFS (right).

Given the link between the immune phenotypes and established prognosticators (e.g., PAM50 subtypes, tumor stage), we then performed survival analysis to explore the prognostic significance of immune phenotype and immune-stromal scores across five selected datasets containing survival data (CBCGA, METABRIC, GSE20486/GSE97177, SCAN-B, and TCGA) for progression-free interval (PFI), OS, and event-free survival (EFS). In the TCGA dataset, the moderate immune phenotype was associated with significantly lower PFI in patients aged 50–64 years compared to the hot and cold phenotypes (Fig. 11a), while the cold immune phenotype exhibited significantly lower PFI in patients of African ancestry (Fig. 11b; *p* < 0.05). No further differences in OS or PFI were found between the immune phenotypes based on age, PAM50 subtype or ancestry (*p* > 0.05).

Next, we explored the possible link between the immune-stromal component and clinical outcome. Patients from each dataset were first categorized into high and low GSVA score groups based on defined cutoff points for immune scores, endothelial scores, fibroblast scores, and an immune stromal ratio (calculated as immune score/[endothelial score + fibroblast score]). Kaplan–Meier analysis and multivariable Cox regression models adjusted for patient age at surgery and/or tumor grade were then performed based on these GSVA score groups (high scores used as reference). In 3/5 datasets, patients with high immune scores had significantly better OS compared to those with lower immune scores, while patients with lower immune stromal ratios exhibited better EFS in 2/5 datasets (Fig. 11c–e; *p* < 0.05). Specifically, low immune scores were associated with a higher risk of EFS in the CBCGA, METABRIC, and TCGA datasets, whereas it was protective in the SCAN-B dataset. Low immune stromal ratios were linked to a protective effect in the SCAN-B and TCGA datasets for EFS and in SCAN-B for OS. Low endothelial scores were associated with an increased risk of OS for the CBCGA, METABRIC, and GSE20486/GSE97177datasets, and EFS for CBCGA, METABRIC, GSE20486/GSE97177, and TCGA. Lastly, low fibroblast scores were significantly associated with an increased risk of OS in CBCGA and METABRIC, but protective in the TCGA dataset for both OS and EFS.

Interestingly, stratification of the TCGA dataset by ancestry (African, Asian, and European) revealed that patients of African descent with low immune scores (*p* = 0.0035) and immune stromal ratios (*p* = 0.0027) had significantly more unfavorable and better PFI, respectively (Supplementary Fig. S4). However, this effect was lost after adjustment for age. Similar trends for OS (immune score and immune stromal ratio) and PFI (fibroblast score) were shown in patients of European ancestry. However, European ancestry was associated with low endothelial scores and an increased risk of PFI, even after adjustment for age (Supplementary Fig. S5).

### Immune phenotypes and treatment response

**Fig. 12:**
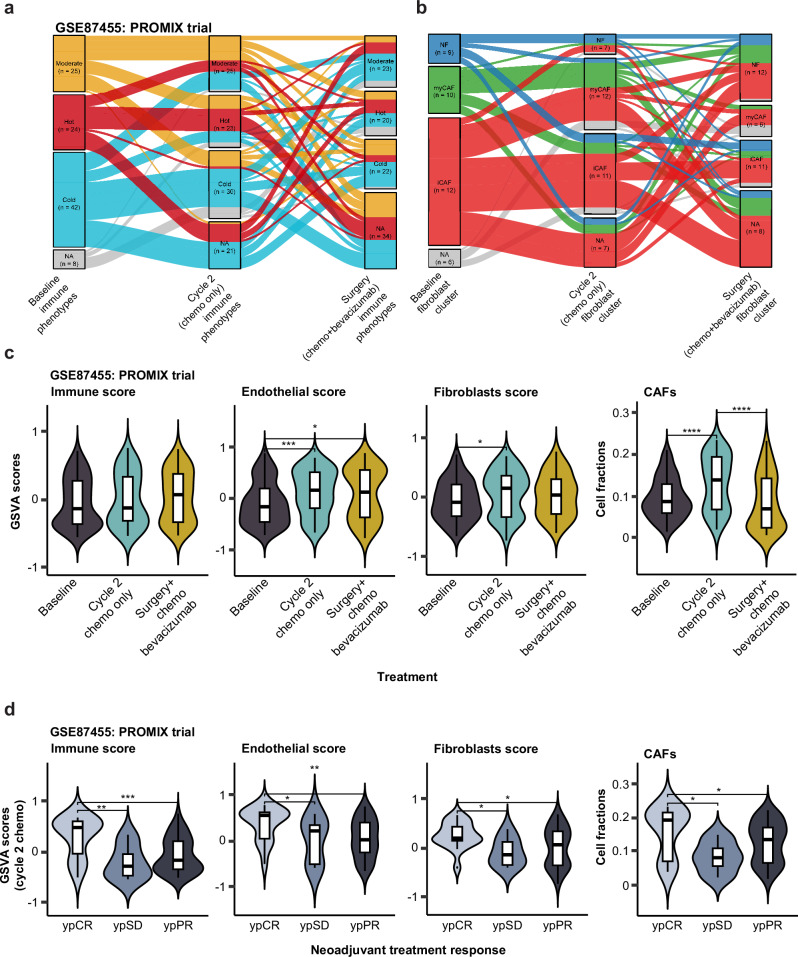
Immune phenotypes and treatment response. **a** Immune phenotype transition across therapy time points for the PROMIX trial (GSE87455). **b** Fibroblast subtype transition across therapy time points for the PROMIX trial (GSE87455). **c** GSVA-derived immune, endothelial, and fibroblast scores across treatment time points. **d** Comparison of scores by treatment response (ypCR, ypSD, and ypPR). CAF Cancer-associated fibroblasts, GSVA score gene set variation analysis score, ypCR pathologic complete response, ypPR partial response, ypSD stable disease. The Wilcoxon test was used to calculate statistically significant differences (Benjamini–Hochberg adjusted *p* values) between the immune phenotype groups. Not significant (*p-adj* > 0.05) and significant (*p-adj < 0.05; **p-adj ≤ 0.01; ***p-adj ≤ 0.001; ****p-adj ≤ 0.0001).

Lastly, we used longitudinal data from the PROMIX trial (GSE87455) to explore the significance of immune phenotypes for predicting treatment response among breast cancer patients undergoing neoadjuvant chemotherapy and surgery. Transitions in immune phenotypes were observed over the course of treatment, with notable changes occurring after the second cycle of therapy with epirubicin and docetaxel, and following an additional cycle of chemotherapy (epirubicin and docetaxel) combined with bevacizumab, after which residual tumors were surgically excised (Fig. 12a). Among the 24 patients who presented with a hot immune phenotype at baseline, 23 retained this phenotype after the second cycle of chemotherapy, and 20 remained hot after completing treatment with neoadjuvant chemotherapy plus bevacizumab and surgery. The remaining patients transitioned to either a cold or moderate phenotype. Of the 25 patients with a moderate baseline immune phenotype, 23 maintained this status throughout treatment, with the remaining two patients transitioning to hot or cold. In contrast, among the 42 patients classified as cold at baseline, only 22 remained cold after treatment, while the remaining shifted to moderate or hot phenotypes. This overall trend indicates that therapy induced an immune-activating effect in the TME for a subset of estrogen receptor positive (ER+) patients. GSVA scores further supported this observation, showing a consistent increase in immune scores from baseline to surgery. Endothelial scores also rose significantly, particularly after the second cycle of chemotherapy, reflecting an increase in vascular-related gene expression. Fibroblast-related scores showed a significant increase, most notably following the addition of bevacizumab to the chemotherapy regimen. Then, we evaluated the role of fibroblasts in breast cancer treatment response in the same cohort from the PROMIX trial. A similar pattern of transitions between fibroblast subtypes was observed, suggesting that fibroblasts may influence treatment outcomes in these patients (Fig. 12b).

Stratification of the cohort by treatment response categorized as pathologic complete response (ypCR), partial response (ypPR) or stable disease (ypSD) revealed that patients achieving ypCR exhibited significantly higher immune scores at cycle 2, as well as elevated endothelial and fibroblast scores (Fig. 12d). Significantly higher delta GSVA endothelial and fibroblast scores (ΔGSVA) between cycle 2 chemotherapy and baseline were found in the ypCR group compared to those with ypSD or ypPR, suggesting that patients achieving complete response also maintain a more robust immune response during treatment (Supplementary Fig. S6a). Similarly, endothelial scores remained significantly higher in the ypCR group compared to ypSD after surgery, though no significant differences were observed between ypCR and ypPR (Supplementary Fig. S6b). In contrast, no significant variation in fibroblast scores was found between the treatment response groups at the time of surgery. Intriguingly, ΔGSVA immune scores were higher in the ypPR group compared to those achieving ypCR.

Deconvolution analysis with the estimate the proportion of immune and cancer cells (EPIC) online tool further revealed that CAF cell fractions were significantly higher in tumors from patients with ypCR compared to those with partial or no response. Together, these findings suggest that effective neoadjuvant therapy in breast cancer not only enhances immune activation but also reshapes the stromal microenvironment, including endothelial and fibroblast components.

## Discussion

In this study, we assess the clinical relevance and ancestry-specific variation in tumor immune phenotypes across diverse populations, focusing on immune-stroma cell profiles, therapeutic response, and survival outcomes. This analysis of over 13,000 breast cancer transcriptomes across globally diverse cohorts provides new insight into ancestry-associated variation in the TME, thereby elucidating potential strategies in therapeutics development for breast cancer based on ancestral lineages. Our findings reveal substantial variation in immune-stromal phenotypes in breast cancer by molecular subtype, age, and ancestry. Hot tumors were prevalent in African, East Asian, and Hispanic patients, the Her2-enriched and Basal-like subtypes, and those <65 years. Cold tumors were more common in European, Southeast Asian, and West Asian patients, the Luminal subtypes, and those >65 years. Fibroblast subtypes varied by ancestry: myCAFs were most common in Southeast Asians, iCAFs in Europeans, West Asians, and Hispanics, and NFs in East Asians. Africans showed a balanced distribution of the fibroblast subtypes. In Europeans, lower immune-stromal ratios predicted better EFS, while iCAFs indicated poor prognosis. Therapy increased immune activation and fibroblast scores, especially post-bevacizumab. Only Africans showed BTLA-mediated T cell co-inhibition. Chemokine receptor-binding was enriched across all ancestries, while East Asians and Hispanics showed unique Leishmania-related immune pathway enrichment.

Genetic ancestry influences both subtype frequency and prognosis^10,11^. For instance, individuals of African ancestry are more likely to be diagnosed with aggressive subtypes such as TNBC, while individuals of European ancestry more frequently present with luminal subtypes^12,13^. These disparities underscore the importance of considering ancestry as a biological and socio-environmental variable in breast cancer research. Understanding how ancestry shapes tumor biology is critical for improving diagnostic accuracy and therapeutic efficacy. Differences in genetic background, gene expression profiles, immune microenvironment, and socioeconomic factors all contribute to the observed disparities in breast cancer incidence and outcomes. Understanding how ancestry shapes tumor biology will provide insights into disease mechanisms and inform the development of equitable and effective precision medicine strategies.

Using a multi-step deconvolution strategy, we characterized the immune and stromal composition of tumors from patients spanning six ancestry groups. While the dataset was predominantly composed of individuals of European ancestry (83%), the inclusion of non-European populations such as African and Hispanic, though comparatively underrepresented, enabled the identification of distinct immunogenomic patterns tied to ancestral origin, reflecting the role of genetic admixture in these populations^11^. The variation in the distribution of immune phenotypes across ancestries suggests underlying ancestry-related differences in tumor immunogenicity and microenvironment composition. The enrichment of Basal-like and Her2-enriched tumors in the Hot phenotype supports prior evidence that these subtypes may be more responsive to immunotherapeutic interventions^14^.

Consistent with a previous study, younger patients (<65 years) were more likely to present with Hot tumors, while those over 75 years were enriched for Cold phenotypes, reflecting age-related immunosenescence or differences in tumor progression^15^. However, logistic regression analysis revealed that immune phenotype was most strongly predicted by PAM50 subtype, age and disease stage, rather than ancestry. Basal-like, Luminal B, and Her2-enriched subtypes were significantly associated with Hot immune phenotypes, while stage IV disease was strongly linked to Cold tumors, suggestive of immune evasion in advanced cancer^16^. Nevertheless, our findings highlight a complex interplay between ancestry, tumor subtype, and the TME in breast cancer. African ancestry was associated with elevated immune scores, particularly across adaptive and active innate immune cells, suggesting a more immunologically active TME. These enriched immune signatures also observed in aggressive molecular subtypes such as Basal-like and Her2-enriched tumors may have important implications for treatment response and clinical outcomes, especially in the context of immunotherapy. Despite increased awareness of immunotherapy in Africa, there is still limited accessibility and uptake^17^. Our analysis presents evidence that the African population may benefit from immunotherapy.

Consequently, stromal cell types like endothelial cells and fibroblasts were significantly reduced in tumors from African and Hispanic populations. This diminished stromal presence may affect tumor vascularization, behavior, and therapeutic delivery^18^. Conversely, East Asian, European, and Southeast Asian populations showed higher stromal GSVA scores, indicative of a denser or more fibrotic TME that could potentially hinder immune infiltration and contribute to therapy resistance^18^. Interestingly, despite the lower overall fibroblast scores in the African ancestry group, no specific enrichment of fibroblast subtypes was observed. In contrast, tumors from individuals of European ancestry exhibited a higher proportion of iCAFs, which was associated with poor prognosis. No significant association was found in the TCGA dataset from this study. These differences could partly be explained by the presence of mixed populations in the TGCA dataset. However, we observed a higher proportion of myCAFs in Southeast Asian patients, a trend that requires further validation, but CAF enrichment has repeatedly been linked to poorer outcomes in breast cancer across large cohorts^19^.

This study highlights the prognostic significance of the immune-stromal balance in breast cancer, revealing that higher immune scores are generally associated with improved OS, while lower immune-stromal ratios are linked to better EFS in European populations, suggesting that reduced stromal dominance may favor better outcomes. This is in agreement with the study of Zeng et al.^20^. Our findings also suggest that the interplay between immune and stromal elements shaped by ancestry, age, and tumor subtypes offers a more modulating and accurate predictor of clinical outcomes than either component alone.

Therapy-induced immune activation and stromal remodeling were evident, particularly after bevacizumab. Importantly, elevated CAF fractions in responders suggests that specific fibroblast subtypes may play an active role in facilitating or reflecting therapeutic efficacy. These findings showed that effective neoadjuvant therapy not only triggers immune activation but also reshapes the stromal landscape, including vasculature and fibroblast populations, which may contribute to more favorable clinical outcomes^6^. Overall, the results highlight the predictive value of immune phenotyping and stromal profiling in gauging treatment response and support their potential utility in tailoring breast cancer therapies.

Lastly, differential expression between the hot vs cold immune phenotypes across ancestries revealed common immune genes across ancestries and ancestry-specific gene signatures. Across all ancestries, a core set of immune activation genes, including *CD2*, *CD3*, *CD247* (CD3 zeta chain), *CD48*, *CD84*, *CCL19*, *CXCL10*, and *SLAMF6*, were consistently upregulated in the hot immune phenotype. These genes are integral to T cell signaling and immune cell communication^21–24^. However, variation in the expression of cytotoxic effector molecules such as *GZMA*, *GZMB*, and *GZMK* was observed. While these genes were upregulated in most ancestries, their absence in the African and Hispanic datasets may reflect the presence of a population of dysfunctional or exhausted T cells in these cohorts^25^. Validation of this observation will require larger cohorts from diverse populations. Several lymphocyte activation markers, such as *CD25*^26^*, CD69*^26^*, PRF1*^27^, and *IFN-Ɣ*^28^ were not upregulated in both hot and cold tumors, suggesting a possible lack of T cell activation in the study cohorts. Interestingly, *CD163*, a marker of M2-like macrophages often associated with immunosuppression, was upregulated mostly in Southeast Asians. CD163-positive cancer cells were shown to be a predictor of worse clinical outcome in lung adenocarcinoma and squamous cell carcinoma^29^.

To validate these transcriptomic findings, immunofluorescence staining was performed on breast cancer samples exhibiting strong and weak inflammatory profiles. Expression of CD8 and PD-L1 was observed in samples with weak and strong inflammation, with strong inflammation having higher expression, while GZMB showed low expression in both inflammation states. High CD8 plus low GZMB expression has been reported to be associated with poor prognosis of lung adenocarcinoma^30^. In our study cohort, lower GZMB expression further suggests the lack of T cell activation, warranting further validation of T cell activation markers such as CD69 and CD25, PRF1 and IFN*-Ɣ*.

Pathway analysis revealed widespread activation of immune signaling pathways, which are hallmarks of active immune surveillance within tumors. However, additional ancestry-specific enrichments were observed. The African dataset uniquely exhibited upregulation of BTLA-mediated T cell co-inhibition, suggesting distinct checkpoint regulatory mechanisms that can potentially be a target for immunotherapy in the African population^31^. However, a larger sample size is needed to validate our findings for this cohort. Interestingly, a single pathway was consistent across the ancestries, i.e., chemokine receptors binding chemokines pathway. Chemokines and chemokine receptors are known to play a significant part in carcinogenesis with an unclear regulatory mechanism^32^. However, chemokines can promote the proliferation and survival of cancer cells by initiating different pathways^32^. These pathways may serve as potential therapeutic targets that can be beneficial to most individuals of different ancestral backgrounds. More importantly, pathway enrichment analyses revealed signatures overlapping with immune responses to *Leishmania* infection in the East Asian and Hispanic cohorts. While not direct evidence of infection, these findings suggest potential shared immune regulatory mechanisms between parasitic disease and cancer^33^.

A major limitation of this study is the underrepresentation of certain ancestries, particularly African and Southeast Asian populations, which is largely attributable to the limited availability of publicly accessible transcriptomic datasets. This imbalance in ancestry representation may restrict the generalizability of our findings across diverse populations. Moreover, the lack of access to raw data further constrained our ability to perform detailed molecular characterization of ancestry, such as investigating population-specific genetic signatures or disentangling fine-scale heterogeneity. Although we were able to validate some of our observations in a cohort with a more balanced sample size and further supported these findings using an external dataset for proteomic validation, these validations remain preliminary, and additional studies are required to confirm their robustness across ancestries.

Another important consideration is the role of genetic admixture. Populations such as Hispanic, African American, and European individuals frequently demonstrate varying degrees of admixture, which may blur ancestry-specific molecular distinctions. This admixture could have contributed to the overlaps observed in some of our analyses, complicating the interpretation of ancestry-driven effects. Taken together, these factors highlight the urgent need for larger, more diverse transcriptomic datasets, with raw data availability, to enable deeper molecular resolution and more equitable representation of ancestries in future studies^34^.

In summary, this study reveals both conserved and ancestry-specific immune signatures underlying hot/cold tumor phenotypes. While immune activation is a consistent feature of hot tumors, the involvement of distinct effector molecules, immunoregulatory pathways, and hormonal influences varies by ancestry. Although immune phenotypes are mostly associated with molecular subtypes, age, and tumor stage, there is still a need to consider population-specific immune biology in cancer research and immunotherapy design.

## Methods

### Transcriptomics profiling data

To evaluate differences in immune phenotype based on ancestry, publicly available gene expression profiling (RNA-seq or RNA microarray) and clinical data were retrieved from various data repositories for 13,731 patients with invasive breast cancer. Treatment-naïve patients or those treated with chemotherapy with primary or metastatic tumors were included. The datasets were classified according to 6 ancestry categories (African, East Asian, European, Hispanic, Southeast Asian, and West Asian) using self-reported racial categories included in the clinical data or based on the geographical region of the study’s country of origin (e.g., studies from China were classified as East Asian). Self-reported categories were reclassified as follows: Asian to East Asian, Black/African American to African, Hispanic/Latino to Hispanic, Middle Eastern/North African to West Asian, and White/Caucasian to European. The following accession numbers were obtained from the Gene Expression Omnibus (GEO) database: GSE20486/GSE97177, GSE260693, GSE264252, GSE96058, GSE15852, GSE20194, GSE37751, GSE78958, GSE48390, GSE54002, GSE2109, GSE75678, GSE86374, GSE113184, GSE20271, GSE59590, GSE211167, GSE87455, and GSE65194^11,12,35–51^. The Cancer Genome Atlas (TCGA) dataset was retrieved via the Xena Browser, METABRIC data from Kaggle, PRJCA017539 from the National Genomics Data Centre^52^, and the Breast Cancer Qatar dataset RA-QA was obtained from Figshare (Supplementary Table 4).

### Sample collection

Formalin-fixed paraffin-embedded (FFPE) specimens corresponding to primary invasive breast cancer were obtained from the Department of Clinical Pathology at Sahlgrenska University Hospital in Gothenburg, Sweden (*n* = 71; from dataset GSE20486/GSE97177). Clinical data were retrieved from patient medical records and/or national registries^1^. Human tissue was handled in accordance with Swedish law, as well as internationally established principles for Good Clinical Practice originating from the Declaration of Helsinki and its revisions. Ethical approval for collecting, biobanking, and performing molecular analyses on human cancer/normal tissue, along with patient information, was obtained from the Central Ethical Review Board (S164-02 and 768-14), Swedish Ethical Review Authority (2019-05676; 2021-04421; 2022-02946-02).

### Immune cell fraction deconvolution

Transcriptomics-based immune cell fraction deconvolution of the gene expression profiling data was performed in two steps. First, the online CIBERSORTx algorithm was used for each dataset separately to estimate the robustness of the 22 immune cell types within the TME (*p* < 0.05)^53^. The LM22 gene expression signature matrix, derived from melanoma tumor biopsies, was used to deconvolute immune cells while applying S-mode batch correction to minimize the impact of cross-platform variation. The analysis run mode was set to “absolute” with 100 permutations. For RNA-seq data, quantile normalization was disabled. Samples with *p* > 0.05 were removed from further analysis. Duplicate microarray gene symbols were removed. Then, each dataset was analyzed using ConsensusTME (version 0.0.1.9000)^54^ with the GSVA method in R/Bioconductor 4.2.2 to calculate enrichment scores for 18 immune cell types (16 immune and 2 stroma cells). ConsensusTME uses a consensus approach from multiple TME cell type estimation tools (CIBERSORTx, MCP-Counter, TIMER, and xCell^55–57^). Given the ConsensusTME GSVA scores, immune phenotypes (Hot [immune infiltrate], Moderate [immune excluded] or Cold [immune desert]) were assigned to each sample using the NbClust (version 3.0.1)^58^ package with k-means clustering and row scaling. Intrinsic breast cancer subtyping was performed using the genefu (version 2.30.0)^59^ package. An immune stromal ratio was calculated using the following equation: GSVA immune score/(endothelial score + fibroblast score).

Fibroblast subtype classification was performed using gene expression count data corresponding to marker genes specific to different fibroblast phenotypes. The classification included three fibroblast subtypes: NF, iCAFs, and myCAFs. Marker genes for each subtype were selected based on previous literature^60^. The NF gene set included *CFD, GSN, GPX3, SOD3, IGFBP6, CLU, CLEC3B, MT2A, APOD, PI16, SLPI, PLA2G2A, MT1M*, and *MT1X*. The iCAF gene set consisted of *CTHRC1, IL24, COL3A1, COL1A1, SPARC, LUM, ASPN, CHI3L1, COL1A2, IGF1, CXCL8, CXCL3, CCN1, CXCL1*, and *IGFBP3*. The myCAF gene set comprised *POSTN, MMP11, CTHRC1, MMP1, COL1A1, SPARC, MFAP2, SERPINH1, COL3A1, COL1A2, THY1, TAGLN, TPM2, SFRP4*, and *ACTA2*. Raw count data for all marker genes were z-score normalized on a per-gene basis to standardize expression values across samples. K-means clustering was then applied to the normalized data to identify three distinct fibroblast clusters. A fixed random seed (set to 123) was used to ensure reproducibility of the clustering results. To assign biological identities to the resulting clusters, representative marker subsets were defined for each fibroblast subtype. Clusters were scored based on the average normalized expression of these marker subsets. The cluster with the highest average expression of *POSTN, ACTA2, MMP11*, and *TAGLN* was annotated as myCAF; the cluster enriched in *IL24, CXCL8, CXCL1*, and *IGFBP3* was labeled as iCAF; and the cluster expressing high levels of *PI16, CFD, SLPI, APOD*, and *CLEC3B* was labeled as NF. The Estimate the Proportion of Immune and Cancer cells (EPIC) online tool was used to deconvolute CAF cell fractions and assess their association with corresponding treatment response outcomes in patients.

### Immunohistochemical analysis and digital pathology

Four-micrometer FFPE sections were first pretreated with the Dako EnvisionTM FLEX High pH Link Kit (pH 9) in the Dako PTLink system (Dako, Carpinteria, CA, USA). Following pretreatment, the sections were processed on an automated Dako Autostainer platform using a ready-to-use monoclonal mouse anti-human CD8 antibody (Agilent, clone CD8/144B). Peroxidase-catalyzed diaminobenzidine was used as the chromogen, followed by hematoxylin counterstain. One FFPE section was also stained with hematoxylin and eosin (H&E) for histological assessment. The stained slides were scanned using Olympus scanner ×20 magnification, viewed using Olyvia image viewer (version 4.1), and were evaluated by a board-certified pathologist and QuPath (version 0.5.1) for digital pathology.

### Spatial proteomics

Multiplexed spatial proteomic analysis was performed using the Phenocycler platform (Akoya Biosciences) according to the manufacturer’s protocols at the SciLifeLab Spatial Proteomics unit in Stockholm, Sweden. Tissue sections (FFPE) were cut to a thickness of 5 µm and mounted onto SuperFrost Plus microscope slides (ThermoFisher Scientific). A quality control was performed (after Dewaxing, Antigen Retrieval, and Photobleaching) to ensure retained attachment and quality of samples.

Multiplexed antibody staining was conducted using a validated panel of 17 DNA-barcoded antibodies (CD38, CD3e, CD4, CD8, CD44, CD79A, CD163, FOXP3, GZMB, IDO1, IFNG, Ki67, PD1, PD-L1, Pan-cytokeratin, TCF-1, and TIGIT) and conjugated to a unique oligonucleotide barcode^61–63^. High-resolution images were acquired using the integrated microscope system. (a) Image size: 15.6 mm × 32.9 mm, (b) Magnification: x20, (0.51 m/pixel), (c) Size in Pixels: 30720 × 64800, (d) Filters and Exposure time, DAPI: 1 ms for all samples; Atto550, Cy5, and AF750 vary depending on marker and sample. Tissue autofluorescence and background signals were minimized through optimized exposure settings and post-processing.

### Differential gene expression and pathway analysis

Representative datasets for each ancestry group (African [GSE211167], East Asian [PRJCA017539], European [GSE20486/GSE97177], Hispanic [GSE86374], Southeast Asian [GSE54002], West Asian [GSE29044], and Mixed [TCGA dataset]) were selected for DGE analysis. In brief, differentially expressed genes (log2fold change > |2| and *p-adj* < 0.05) between the hot and cold immune phenotypes were identified using DESeq2 (version 1.46.0)^64^ for RNA-seq data or limma (3.62.2) for microarray data. The differentially regulated genes (log2fold change > |2| and *p-adj* < 0.00001) were selected and analyzed in Reactome^65^ an online platform. Bar charts were used to illustrate the significant pathways (*p* < 0.05) for each selected dataset^66^.

### Statistical analysis

R/Bioconductor (R version 4.4.2) was used for statistical analysis, with a significance level of 0.05 and two-sided *p* values. The relationship between clinicopathological features and the immune phenotypes was evaluated using the tableone package (version 0.13.267) with the Chi-square test (with continuity correction) for categorical variables and ANOVA for continuous variables. Hierarchical clustering of gene expression patterns was performed using the pheatmap R package (version 1.0.12) with scaled rows. Box plots and ggforestplots were generated with the ggpubr (version 0.6.0)^67^ and rstatix (version 0.7.2)^68^ packages with Wilcoxon test and Benjamini–Hochberg adjusted *p* values. Cardinality matching was performed using the R package *designmatch* (version 0.5.4) to balance sample size across ancestry groups. EnhancedVolcano (version 1.24.0)^69^ was used to visualize significant differentially expressed genes between the hot and cold immune phenotypes among ancestries. Kaplan–Meier survival analysis was performed to estimate survival distributions and assess differences between immune phenotype groups using R packages “survminer” (version 0.4.9), “survival” (version 3.4-0), and gtsummary (version 1.7.2) packages to determine the optimal cutpoint for the variables and survival analysis. Multivariable Cox regression models were calculated for the immune phenotypes, adjusting for patient age and/or tumor grade. OS was defined as the time from the date of diagnosis to death of any cause. EFS was defined as the time from the date of diagnosis to an event (whichever occurred first: local recurrence, distant metastasis or progression) using either disease-specific survival, distant metastasis-free survival, distant recurrence-free interval, PFI, or recurrence-free survival. Alluvial plots were used to visualize changes in immune phenotypes before and after treatment in patients.

## Supplementary information

Supplementary material Aamukii et al.2025-October

## Data Availability

Transcriptomic datasets analyzed in this study are publicly available. GEO accession numbers include: GSE20486, GSE97177, GSE260693, GSE264252, GSE96058, GSE15852, GSE20194, GSE37751, GSE78958, GSE48390, GSE54002, GSE2109, GSE75678, GSE86374, GSE113184, GSE20271, GSE59590, GSE211167, GSE87455, and GSE65194. TCGA data were accessed via the Xena Browser, METABRIC from Kaggle, PRJCA017539 from the National Genomics Data Centre, and the Breast Cancer Qatar RA-QA dataset from Figshare. Links to these datasets are listed in the Supplementary File.

## References

[1] Francies, F. Z., Hull, R., Khanyile, R. & Dlamini, Z. Breast cancer in low-middle income countries: abnormality in splicing and lack of targeted treatment options. *Am. J. Cancer Res.* **10**, 1568 (2020). 32509398 PMC7269781

[2] Kim, J. et al. Global patterns and trends in breast cancer incidence and mortality across 185 countries. *Nat. Med.* **31**, 1154–1162 (2025). 39994475 10.1038/s41591-025-03502-3

[3] Telonis, A. G., Rodriguez, D. A., Spanheimer, P. M., Figueroa, M. E. & Goel, N. Genetic ancestry-specific molecular and survival differences in admixed patients with breast cancer. *Ann. Surg.* **279**, 866–873 (2024). 38073557 10.1097/SLA.0000000000006135 PMC11611248

[4] Loibl, S. et al. Neoadjuvant durvalumab improves survival in early triple-negative breast cancer independent of pathological complete response. *Ann. Oncol.* **33**, 1149–1158 (2022). 35961599 10.1016/j.annonc.2022.07.1940

[5] Luo, M. et al. Ancestral differences in anticancer treatment efficacy and their underlying genomic and molecular alterations. *Cancer Discov* **15**, 511–529 (2025). 39601595 10.1158/2159-8290.CD-24-0827 PMC11875934

[6] Xu, Y., Zhang, B., Wu, H. & Wu, Y. Current status of breast cancer immunotherapy and prognosis-related markers. *Breast Cancer Targets Ther* **17**, 339–348 (2025). 10.2147/BCTT.S506949 PMC12009046 40256248

[7] Du, Q. et al. PD-L1 acts as a promising immune marker to predict the response to neoadjuvant chemotherapy in breast cancer patients. *Clin*. *Breast Cancer* **20**, e99–e111 (2020). 31521537 10.1016/j.clbc.2019.06.014

[8] Jiang, C. et al. High PD-L1 expression is associated with a favorable prognosis in patients with esophageal squamous cell carcinoma undergoing postoperative adjuvant radiotherapy. *Oncol. Lett.* **17**, 1626 (2018). 30675222 10.3892/ol.2018.9747 PMC6341902

[9] Oshi, M. et al. CD8 T cell score as a prognostic biomarker for triple negative breast cancer. *Int. J. Mol. Sci.* **21**, 6968 (2020). 32971948 10.3390/ijms21186968 PMC7555570

[10] Hayat, M. et al. Genome-wide association study identifies common variants associated with breast cancer in South African Black women. *Nat Commun* **16**, 3542 (2025). 40229280 10.1038/s41467-025-58789-0 PMC11997036

[11] Roelands, J. et al. Ancestry-associated transcriptomic profiles of breast cancer in patients of African, Arab, and European ancestry. *NPJ Breast Cancer* **7**, 10 (2021). 33558495 10.1038/s41523-021-00215-x PMC7870839

[12] Martini, R. et al. African ancestry–associated gene expression profiles in triple-negative breast cancer underlie altered tumor biology and clinical outcome in women of African descent. *Cancer Discov* **12**, 2530–2551 (2022). 36121736 10.1158/2159-8290.CD-22-0138 PMC9627137

[13] Prakash, O. et al. Racial disparities in triple negative breast cancer: a review of the role of biologic and non-biologic factors. *Front. Public Health* **8**, 576964 (2020). 33415093 10.3389/fpubh.2020.576964 PMC7783321

[14] Onkar, S. S. et al. The great immune escape: understanding the divergent immune response in breast cancer subtypes. *Cancer Discov* **13**, 23 (2023). 36620880 10.1158/2159-8290.CD-22-0475 PMC9833841

[15] Zirbes, A. et al. Changes in immune cell types with age in breast are consistent with a decline in immune surveillance and increased immunosuppression. *J. Mammary Gland Biol. Neoplasia* **26**, 247 (2021). 34341887 10.1007/s10911-021-09495-2 PMC8566425

[16] Whiteside, T. L. The tumor microenvironment and its role in promoting tumor growth. *Oncogene* **27**, 5904 (2008). 18836471 10.1038/onc.2008.271 PMC3689267

[17] Olatunji, E. et al. Utilization of cancer immunotherapy in sub-Saharan Africa. *Front. Oncol.* **13**, 1266514 (2023). 38179176 10.3389/fonc.2023.1266514 PMC10765613

[18] Kuo, C. et al. Tumor-associated stroma shapes the spatial tumor immune microenvironment of primary Ewing sarcomas. *Clin Cancer Res* **31**, 5051–5069 (2025). 40986513 10.1158/1078-0432.CCR-25-0635 PMC12700766

[19] Cui, M. et al. The relationship between cancer associated fibroblasts biomarkers and prognosis of breast cancer: a systematic review and meta-analysis. *PeerJ* **12**, e16958 (2024). 38410801 10.7717/peerj.16958 PMC10896086

[20] Zeng, Z. et al. Immune and stromal scoring system associated with tumor microenvironment and prognosis: a gene-based multi-cancer analysis. *J. Transl. Med.* **19**, 1–18 (2021). 34344410 10.1186/s12967-021-03002-1 PMC8336334

[21] Oba, T., Long, M. D., Ito, K., Ichi & Ito, F. Clinical and immunological relevance of SLAMF6 expression in the tumor microenvironment of breast cancer and melanoma. *Sci. Rep.* **14**, 2394 (2024). 38287061 10.1038/s41598-023-50062-y PMC10825192

[22] Kersh, A. E. et al. CXCL9, CXCL10, and CCL19 synergistically recruit T lymphocytes to skin in lichen planus. *JCI Insight* **9**, e179899 (2024). 39190494 10.1172/jci.insight.179899 PMC11533982

[23] Jin, Y., Yuan, H., Mehta, I., Ezenwa, O. & Morel, P. A. Alternatively spliced variants of murine CD247 influence T cell development and activation, revealing the importance of the CD3ζ C-terminal region. *J. Immunol.* **212**, 541–550 (2024). 38117282 10.4049/jimmunol.2300511 PMC10872740

[24] McArdel, S. L., Terhorst, C. & Sharpe, A. H. Roles of CD48 in regulating immunity and tolerance. *Clin. Immunol.* **164**, 10 (2016). 26794910 10.1016/j.clim.2016.01.008 PMC4860950

[25] Ni, L. Potential mechanisms of cancer stem-like progenitor T-cell bio-behaviours. *Clin. Transl. Med.* **14**, e1817 (2024). 39169517 10.1002/ctm2.1817 PMC11338842

[26] Adamczyk, M. et al. The expression of activation markers CD25 and CD69 increases during biologic treatment of psoriasis. *J. Clin. Med.* **12**, 6573 (2023). 37892710 10.3390/jcm12206573 PMC10607364

[27] Martínez-Lostao, L., Anel, A. & Pardo, J. How do cytotoxic lymphocytes kill cancer cells? *Clin. Cancer Res.* **21**, 5047–5056 (2015). 26567364 10.1158/1078-0432.CCR-15-0685

[28] Bhat, P., Leggatt, G., Waterhouse, N. & Frazer, I. H. Interferon-γ derived from cytotoxic lymphocytes directly enhances their motility and cytotoxicity. *Cell Death Dis* **8**, e2836–e2836 (2017). 28569770 10.1038/cddis.2017.67 PMC5520949

[29] Matsubara, E. et al. CD163-positive cancer cells are a predictor of a worse clinical course in lung adenocarcinoma. *Pathol. Int.* **71**, 666–673 (2021). 34231937 10.1111/pin.13144

[30] Kinoshita, F. et al. Granzyme B (GZMB)-positive tumor-infiltrating lymphocytes in lung adenocarcinoma: significance as a prognostic factor and association with immunosuppressive proteins. *Ann. Surg. Oncol.* **30**, 7579–7589 (2023). 37587364 10.1245/s10434-023-14085-z

[31] Pardoll, D. M. The blockade of immune checkpoints in cancer immunotherapy. *Nat. Rev. Cancer* **12**, 252–264 (2012). 22437870 10.1038/nrc3239 PMC4856023

[32] Yi, M. et al. Targeting cytokine and chemokine signaling pathways for cancer therapy. *Signal Transduct. Target. Ther. 2024* **9**, 1–48 (2024). 10.1038/s41392-024-01868-3 PMC11275440 39034318

[33] Rashidi, S. et al. Potential therapeutic targets shared between leishmaniasis and cancer. *Parasitology* **148**, 655 (2021). 33536086 10.1017/S0031182021000160 PMC10090780

[34] Bryc, K., Durand, E. Y., Macpherson, J. M., Reich, D. & Mountain, J. L. The Genetic Ancestry of African Americans, Latinos, and European Americans across the United States. *Am. J. Hum. Genet.* **96**, 37 (2015). 25529636 10.1016/j.ajhg.2014.11.010 PMC4289685

[35] Parris, T. Z. et al. Frequent MYC coamplification and DNA hypomethylation of multiple genes on 8q in 8p11-p12-amplified breast carcinomas. *Oncogenesis.* **3**, e95 (2014). 24662924 10.1038/oncsis.2014.8 PMC4038389

[36] Saal, L. H. et al. The Sweden Cancerome Analysis Network - Breast (SCAN-B) Initiative: a large-scale multicenter infrastructure towards implementation of breast cancer genomic analyses in the clinical routine. *Genome Med* **7**, 20 (2015). 25722745 10.1186/s13073-015-0131-9 PMC4341872

[37] Maubant, S. et al. Transcriptome analysis of wnt3a-treated triple-negative breast cancer cells. *PLoS ONE* **10**, e0122333 (2015). 25848952 10.1371/journal.pone.0122333 PMC4388387

[38] Pau Ni, I. B. et al. Gene expression patterns distinguish breast carcinomas from normal breast tissues: the Malaysian context. *Pathol. Res. Pract.* **206**, 223–228 (2010). 20097481 10.1016/j.prp.2009.11.006

[39] Popovici, V. et al. Effect of training-sample size and classification difficulty on the accuracy of genomic predictors. *Breast Cancer Res* **12**, R5 (2010). 20064235 10.1186/bcr2468 PMC2880423

[40] Tabchy, A. et al. Evaluation of a 30-gene paclitaxel, fluorouracil, doxorubicin, and cyclophosphamide chemotherapy response predictor in a multicenter randomized trial in breast cancer. *Clin. Cancer Res.* **16**, 5351–5361 (2010). 20829329 10.1158/1078-0432.CCR-10-1265 PMC4181852

[41] Tang, W. et al. Integrated proteotranscriptomics of breast cancer reveals globally increased protein-mRNA concordance associated with subtypes and survival. *Genome Med* **10**, 94 (2018). 30501643 10.1186/s13073-018-0602-x PMC6276229

[42] Chen, Y. J. et al. Molecular subtyping of breast cancer intrinsic taxonomy with oligonucleotide microarray and NanoString nCounter. *Biosci. Rep.* **41**, BSR20211428 (2021). 34387660 10.1042/BSR20211428 PMC8385191

[43] Tan, T. Z. et al. Epithelial-mesenchymal transition spectrum quantification and its efficacy in deciphering survival and drug responses of cancer patients. *EMBO Mol. Med.* **6**, 1279–1293 (2014). 25214461 10.15252/emmm.201404208 PMC4287932

[44] Tamez-Peña, J. G. et al. Radiogenomics analysis identifies correlations of digital mammography with clinical molecular signatures in breast cancer. *PLoS ONE* **13**, e0193871 (2018). 29596496 10.1371/journal.pone.0193871 PMC5875760

[45] Kan, Z. et al. Multi-omics profiling of younger Asian breast cancers reveals distinctive molecular signatures. *Nat. Commun.* **9**, 1725 (2018). 29713003 10.1038/s41467-018-04129-4 PMC5928087

[46] Colak, D. et al. Age-specific gene expression signatures for breast tumors and cross-species conserved potential cancer progression markers in young women. *PLoS ONE* **8**, e63204 (2013). 23704896 10.1371/journal.pone.0063204 PMC3660335

[47] Merdad, A. et al. Expression of matrix metalloproteinases (MMPs) in primary human breast cancer: MMP-9 as a potential biomarker for cancer invasion and metastasis. *Anticancer Res* **34**, 1355–1366 (2014). 24596383

[48] Zhang, Y. et al. The 76-gene signature defines high-risk patients that benefit from adjuvant tamoxifen therapy. *Breast Cancer Res. Treat* **116**, 303–309 (2009). 18821012 10.1007/s10549-008-0183-2

[49] Bedi, D., Martini, R., Davis, M. B. & Yates, C. Abstract B101: obesity mediates altered triple-negative breast cancer tumor biology in African American women independent of ancestry. *Cancer Epidemiol. Biomark. Prev.* **32**, 280–290 (2023).

[50] Kimbung, S. et al. Assessment of early response biomarkers in relation to long-term survival in patients with HER2-negative breast cancer receiving neoadjuvant chemotherapy plus bevacizumab: results from the Phase II PROMIX trial. *Int. J. Cancer* **142**, 618 (2017). 28940389 10.1002/ijc.31070 PMC5765477

[51] Parris, T. Z. et al. Clinical implications of gene dosage and gene expression patterns in diploid breast carcinoma. *Clin. Cancer Res.* **16**, 3860–3874 (2010). 20551037 10.1158/1078-0432.CCR-10-0889

[52] Jiang, Y. Z. et al. Integrated multiomic profiling of breast cancer in the Chinese population reveals patient stratification and therapeutic vulnerabilities. *Nat. Cancer* **5**, 673–690 (2024). 38347143 10.1038/s43018-024-00725-0

[53] Newman, A. M. et al. Determining cell type abundance and expression from bulk tissues with digital cytometry. *Nat. Biotechnol.* **37**, 773–782 (2019). 31061481 10.1038/s41587-019-0114-2 PMC6610714

[54] Jimenez-Sanchez, A., Cast, O. & Miller, M. L. Comprehensive benchmarking and integration of tumor microenvironment cell estimation methods. *Cancer Res* **79**, 6238–6246 (2019). 31641033 10.1158/0008-5472.CAN-18-3560

[55] Bindea, G. et al. Spatiotemporal dynamics of intratumoral immune cells reveal the immune landscape in human cancer. *Immunity* **39**, 782–795 (2013). 24138885 10.1016/j.immuni.2013.10.003

[56] Danaher, P. et al. Gene expression markers of Tumor Infiltrating Leukocytes. *J. Immunother. Cancer* **5**, 18 (2017). 28239471 10.1186/s40425-017-0215-8 PMC5319024

[57] Davoli, T., Uno, H., Wooten, E. C. & Elledge, S. J. Tumor aneuploidy correlates with markers of immune evasion and with reduced response to immunotherapy. *Science* **355**, eaaf8399 (2017). 28104840 10.1126/science.aaf8399 PMC5592794

[58] Charrad, M., Ghazzali, N., Boiteau, V. & Niknafs, A. Nbclust: an R package for determining the relevant number of clusters in a data set. *J. Stat. Softw.* **61**, 1–36 (2014).

[59] Gendoo, D. M. A. et al. Genefu: an R/Bioconductor package for computation of gene expression-based signatures in breast cancer. *Bioinformatics* **32**, 1097–1099 (2016). 26607490 10.1093/bioinformatics/btv693 PMC6410906

[60] Chen, Y. et al. Epithelial cells activate fibroblasts to promote esophageal cancer development. *Cancer Cell* **41**, 903–918.e8 (2023). 36963399 10.1016/j.ccell.2023.03.001

[61] Black, S. et al. CODEX multiplexed tissue imaging with DNA-conjugated antibodies. *Nat. Protoc.* **16**, 3802–3835 (2021). 34215862 10.1038/s41596-021-00556-8 PMC8647621

[62] Goltsev, Y. et al. Deep profiling of mouse splenic architecture with CODEX multiplexed imaging. *Cell* **174**, 968–981.e15 (2018). 30078711 10.1016/j.cell.2018.07.010 PMC6086938

[63] Schürch, C. M. et al. Coordinated cellular neighborhoods orchestrate antitumoral immunity at the colorectal cancer invasive front. *Cell* **182**, 1341–1359.e19 (2020). 32763154 10.1016/j.cell.2020.07.005 PMC7479520

[64] Love, M. I., Huber, W. & Anders, S. Moderated estimation of fold change and dispersion for RNA-seq data with DESeq2. *Genome Biol* **15**, 550 (2014). 25516281 10.1186/s13059-014-0550-8 PMC4302049

[65] Milacic, M. et al. The Reactome Pathway Knowledgebase 2024. *Nucleic Acids Res* **52**, D672–D678 (2024). 37941124 10.1093/nar/gkad1025 PMC10767911

[66] Package ‘Tableone’. https://orcid.org/0000-0002-2030-3549 (2022).

[67] Alboukadel Kassambara. ggpubr: ‘ggplot2’ based publication ready plots. *R package version 0.6.0* (2023). https://rpkgs.datanovia.com/ggpubr/.

[68] Alboukadel, K. rstatix: pipe-friendly framework for basic statistical tests_. *R package version 0.7.2* (2023). https://rpkgs.datanovia.com/rstatix

[69] Blighe K, R. S. L. M. EnhancedVolcano: publication-ready volcano plots with enhanced colouring and labeling. R package version 1.20.0 (2023). https://bioconductor.org/packages/EnhancedVolcano.

