# Supplementary material for: Immune-stromal heterogeneity in breast cancer across diverse ancestries: impact on prognosis and treatment response
Source: NPJ Breast Cancer. 2025 Dec 12;11:142. doi: 10.1038/s41523-025-00881-1 (PMC12712059; doi:10.1038/s41523-025-00881-1)
Supplement: Supplementary file 1 — Supplementary material Aamukii et al.2025-October [file 41523_2025_881_MOESM1_ESM.pdf]

Supplementary material

Immune-Stromal Heterogeneity in Breast Cancer Across Diverse Ancestries: Impact on Prognosis and Treatment Response

Nanfizat A. Alamukii, Anikó Kovács, Sukanya Raghavan, Josefin Ilio, Per Karlsson, Khalil Helou, Toshima Z. Parris

Contents

Supplementary Figure 1 3
Supplementary Figure 2 4
Supplementary Figure 3 5
Supplementary Figure 4 6
Supplementary Figure 5 7
Supplementary Figure 6 8
Supplementary Table 1 9
Supplementary Table 2 12
Supplementary Table 3 14
Supplementary Table 4 16
Supplementary Table 5 29

Supplementary Figure 1

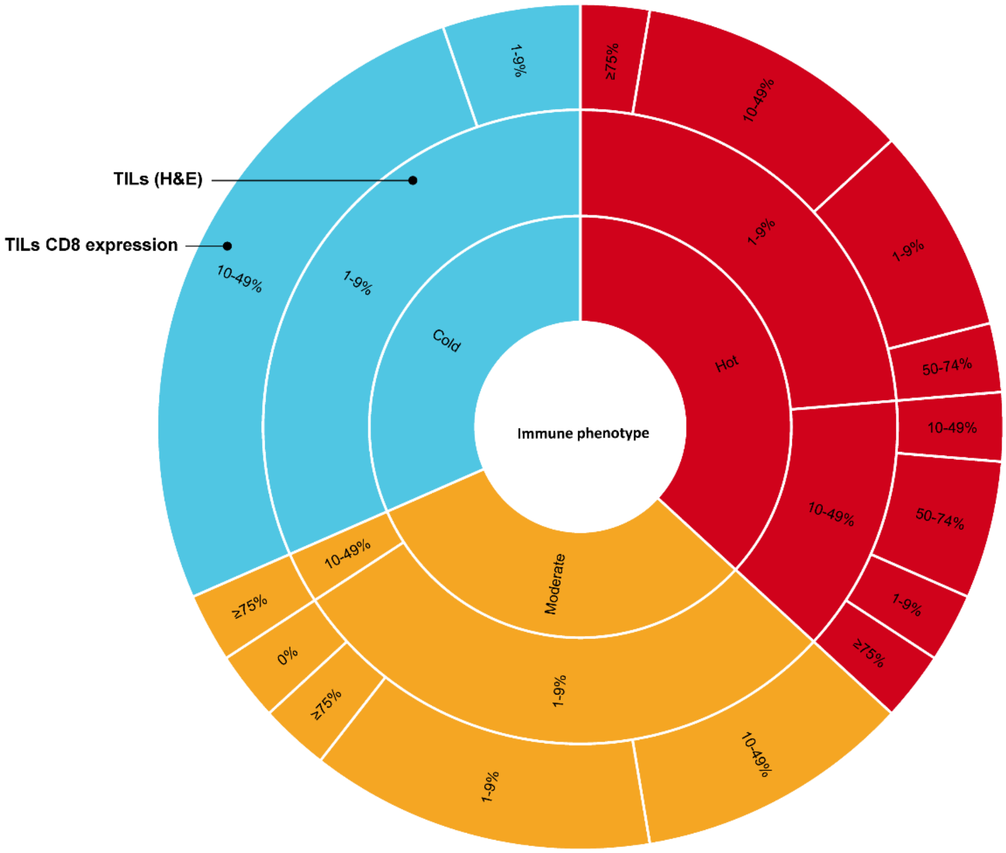

Fig. S1 | Pie chart showing the proportion of tumor-infiltrating lymphocytes (TILs) across immune phenotypes and the proportion of CD8 expression within TILs across the same phenotypes. Tumors with a Hot immune phenotype displayed a higher percentage of TILs compared with Cold and Moderate tumors. Across phenotypes, the proportion of TILs with intermediate CD8 expression (10-49%) was relatively higher**.**

Supplementary Figure 2

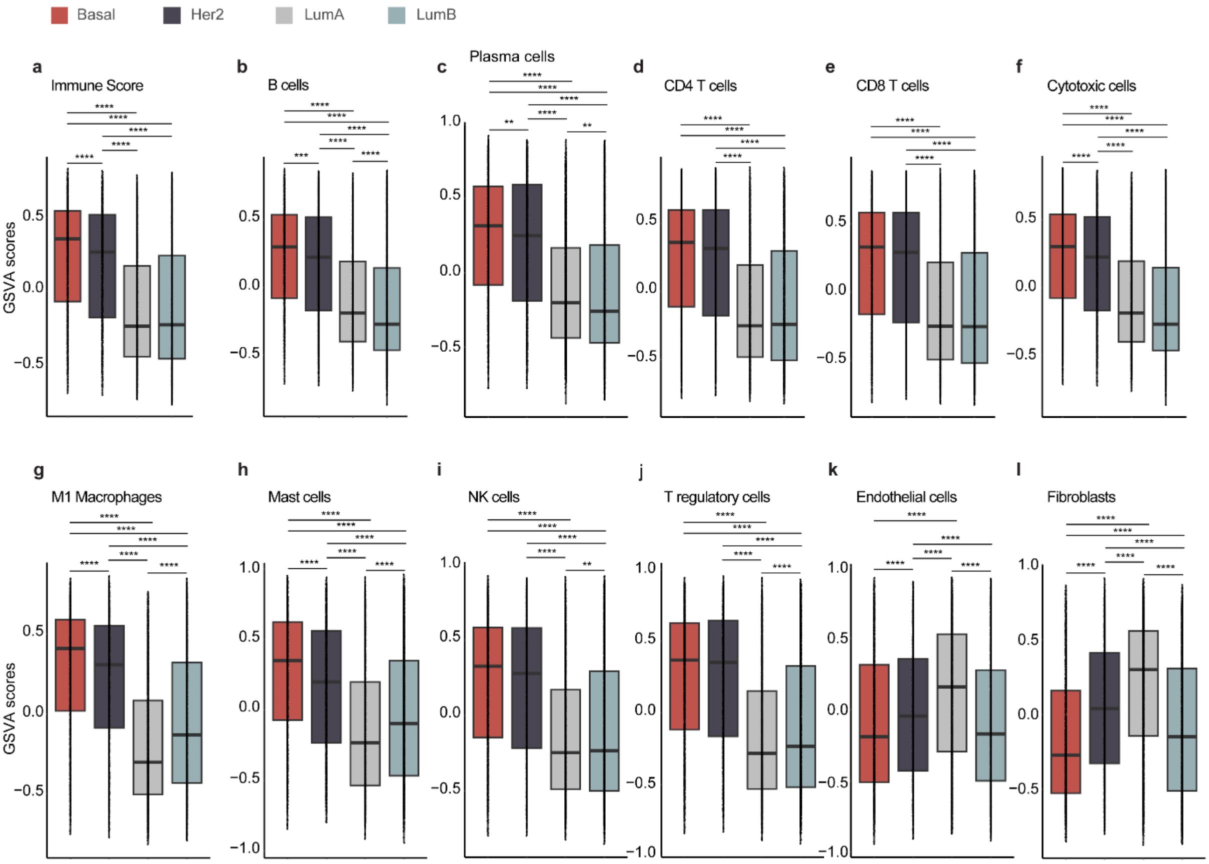

Fig. S2 | Variation in immune and stroma cell composition and gene set variation analysis (GSVA) scores among PAM50 breast cancer subtypes: **Basal-like (Basal), HER2 enriched (Her2), Luminal A (LumA) and Luminal B (Lum B)**. Box plots display the **a** overall immune score and GSVA scores for **b** B cells, **c** plasma cells, **d** CD4+ T cells, **e** CD8+ T cells, **f** cytotoxic cells, **g** M1 macrophages, **h** mast cells, **i** NK cells, **j** T regulatory cells, **k** endothelial cells, and **l** fibroblasts. The Wilcoxon test was used to calculate statistically significant differences (Benjamini-Hochberg adjusted p-values) between the subtypes. Not significant ($p > 0.05$); *$p < 0.05$; **$p \leq 0.01$; ***$p \leq 0.001$; ****$p \leq 0.0001$. Only statistically significant differences are shown in the figure.

Supplementary Figure 3

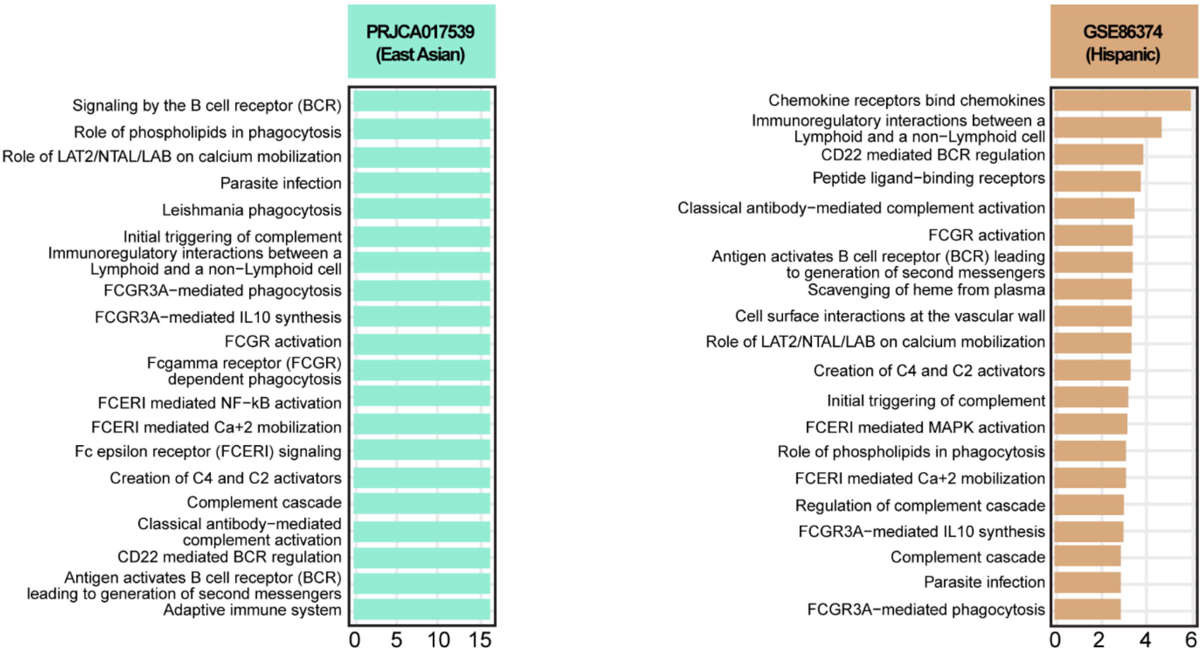

Fig. S3 | Bar plots showing exclusive and comparable enriched leishmaniasis and immune-related pathways in the transcriptomic data of East Asian (PRJCA017539, teal) and Hispanic (GSE86374, brown) populations.

Supplementary Figure 4

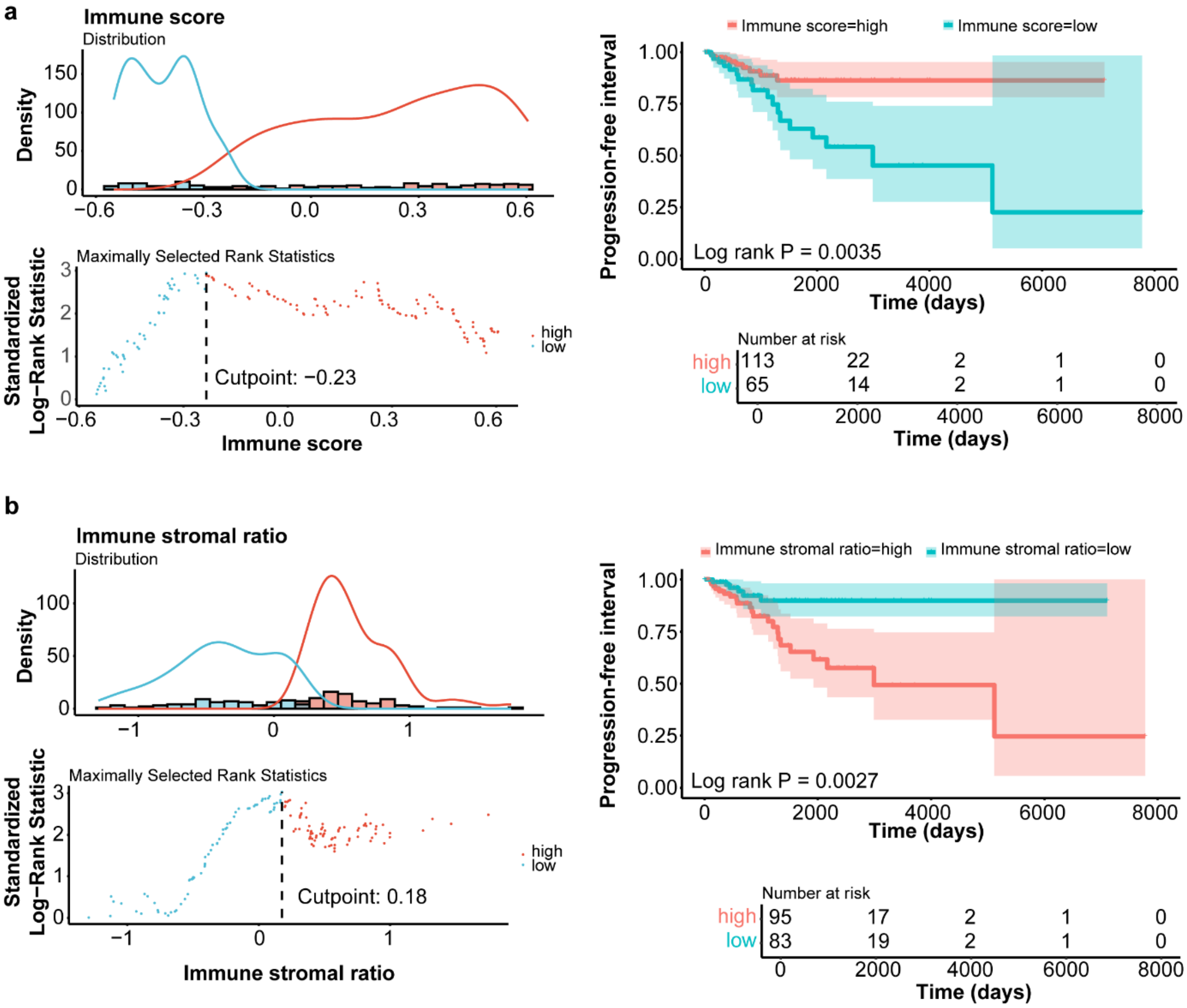

Fig. S4 | Survival analysis of progression-free interval (PFI) and immune and stromal cell scores in TCGA patients of African ancestry. The cutpoint (left) and Kaplan-Meier survival analysis (right) for the association between **a** high and low immune scores and PFI, as well as **b** high and low immune stromal ratio and PFI (p<0.05).

Supplementary Figure 5

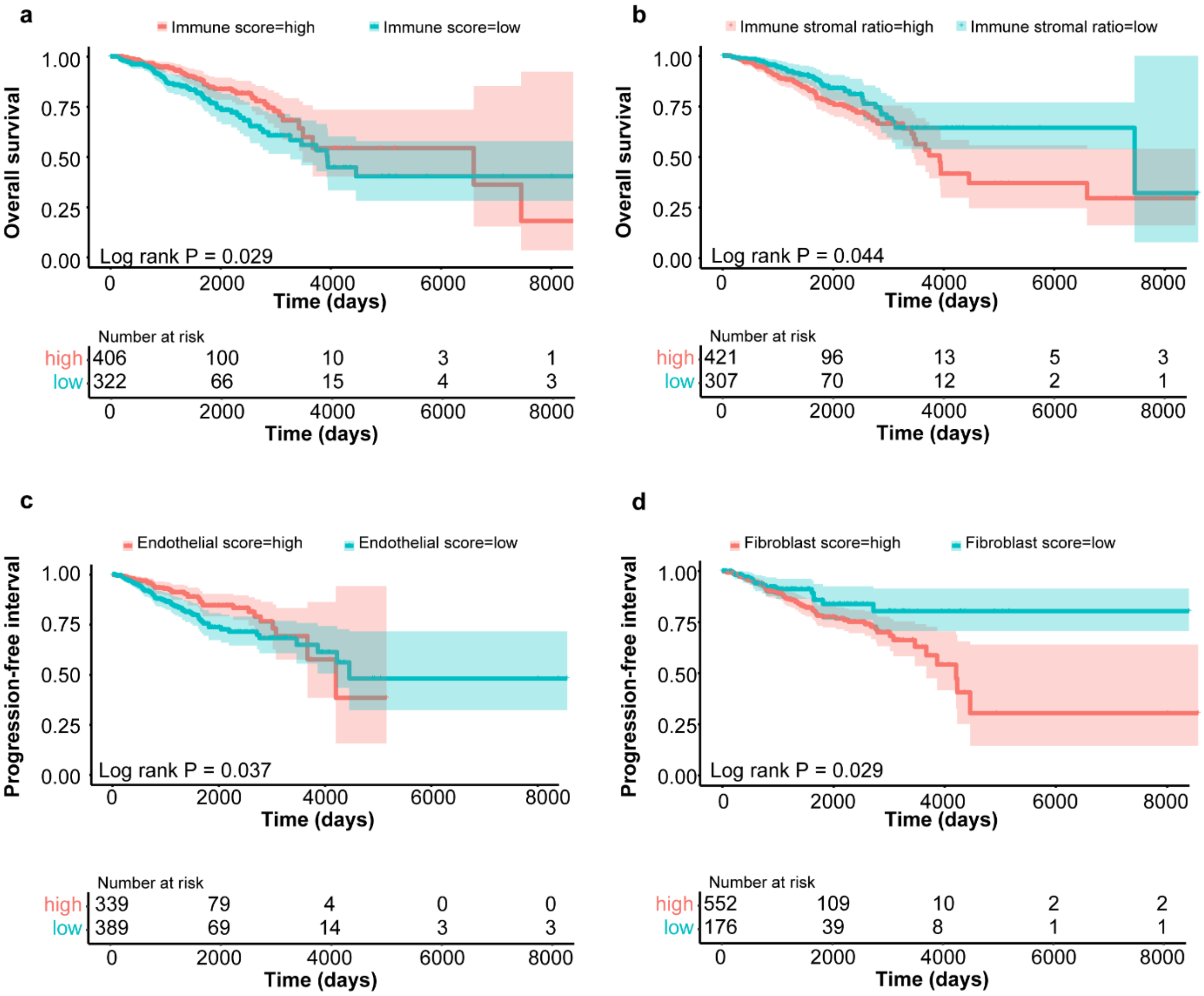

Fig. S5 | Survival analysis of immune and stromal cell scores in TCGA patients of European ancestry. The Kaplan-Meier survival analysis for the association between **a** immune scores and overall survival (OS), **b** immune stromal ratio and OS, **c** endothelial scores and progression-free interval (PFI), and **d** fibroblast scores and PFI (p<0.05).

Supplementary Figure 6

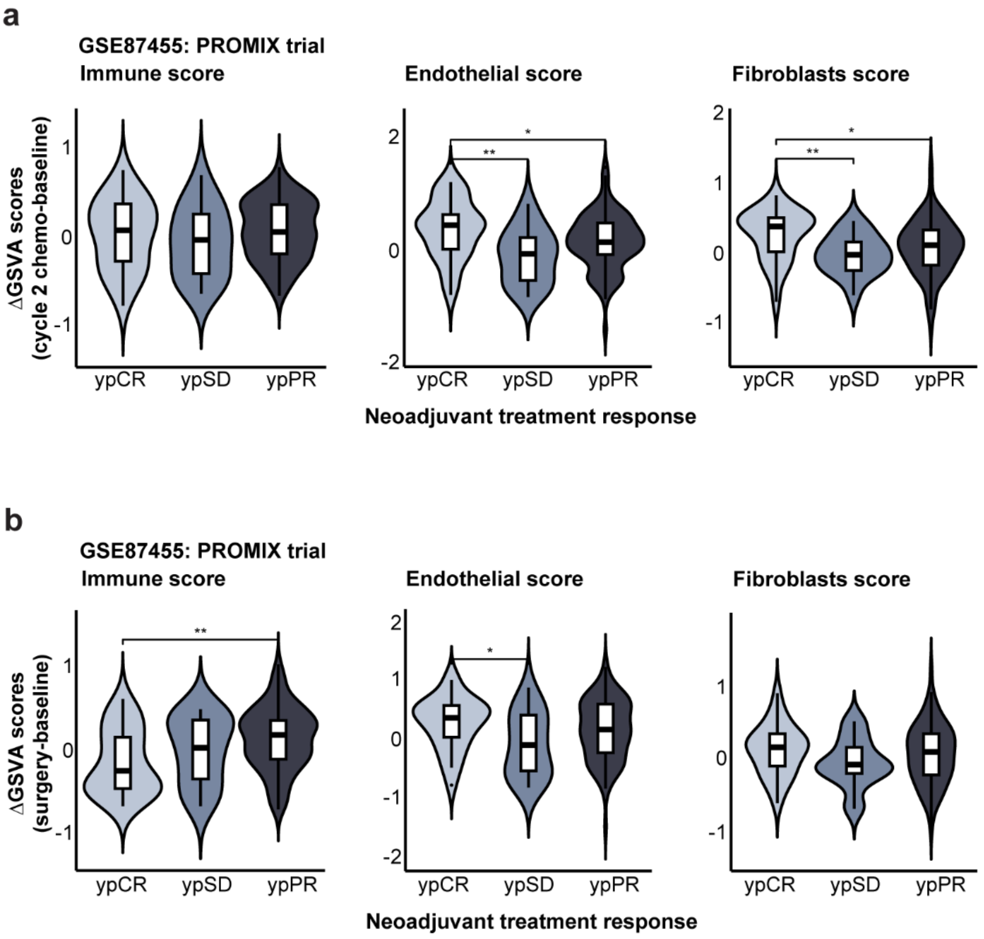

Fig. S6 | Comparison of changes in GSVA scores (ΔGSVA) for immune and stromal scores based on neoadjuvant treatment response of patients in the GSE87455 PROMIX trial dataset. ΔGSVA scores were calculated between **a** cycle 2 chemotherapy and baseline, as well as **b** surgery and baseline. GSVA score: Gene Set Variation Analysis score; ypCR: Pathologic complete response, ypPR: Partial response, ypSD: Stable disease. The Wilcoxon test was used to calculate statistically significant differences (Benjamini-Hochberg adjusted p-values) between the treatment response groups. Not significant (*p-adj* > 0.05) and significant (**p-adj* < 0.05; ***p-adj* ≤ 0.01; ****p-adj* ≤ 0.001; *****p-adj* ≤ 0.0001).

Supplementary Table 1

Clinicopathological characteristics of the 13,731 breast cancer samples included in the study, stratified by ancestry

Characteristic	Overall (n = 13731)	African (n = 384)	East Asian (n = 1035)	European (n = 11437)	Hispanic (n = 300)	Southeast Asian (n = 452)	West Asian (n = 123)	p
**Immune phenotype (%)**								**0.001**
Hot	4260 (31.0)	140 (36.5)	366 (35.4)	3495 (30.6)	109 (36.3)	111 (24.6)	39 (31.7)	
Moderate	4094 (29.8)	110 (28.6)	298 (28.8)	3417 (29.9)	83 (27.7)	149 (33.0)	37 (30.1)	
Cold	5377 (39.2)	134 (34.9)	371 (35.8)	4525 (39.6)	108 (36.0)	192 (42.5)	47 (38.2)	
**Immune score (mean (SD))**	-0.03 (0.42)	0.04 (0.42)	-0.02 (0.47)	-0.03 (0.42)	0.01 (0.39)	-0.02 (0.33)	0.01 (0.39)	**0.022**
**Age group (%)**								**<0.001**
<40 years	583 (4.2)	19 (4.9)	175 (16.9)	346 (3.0)	26 (8.7)	5 (1.1)	12 (9.8)	
40-49 years	1538 (11.2)	32 (8.3)	269 (26.0)	1134 (9.9)	67 (22.3)	14 (3.1)	22 (17.9)	
50-64 years	3739 (27.2)	47 (12.2)	394 (38.1)	3208 (28.0)	60 (20.0)	12 (2.7)	18 (14.6)	
65-74 years	3036 (22.1)	18 (4.7)	76 (7.3)	2918 (25.5)	19 (6.3)	2 (0.4)	3 (2.4)	
≥75 years	2646 (19.3)	10 (2.6)	26 (2.5)	2606 (22.8)	1 (0.3)	2 (0.4)	1 (0.8)	
Missing data	2189 (15.9)	258 (67.2)	95 (9.2)	1225 (10.7)	127 (42.3)	417 (92.3)	67 (54.5)	
**T stage (%)**								**<0.001**
T1	4923 (35.9)	71 (18.5)	19 (1.8)	4828 (42.2)	5 (1.7)	0 (0.0)	0 (0.0)	
T1a	3 (0.0)	0 (0.0)	3 (0.3)	0 (0.0)	0 (0.0)	0 (0.0)	0 (0.0)	
T1b	33 (0.2)	0 (0.0)	33 (3.2)	0 (0.0)	0 (0.0)	0 (0.0)	0 (0.0)	
T1c	272 (2.0)	0 (0.0)	272 (26.3)	0 (0.0)	0 (0.0)	0 (0.0)	0 (0.0)	
T2	3156 (23.0)	83 (21.6)	414 (40.0)	2646 (23.1)	13 (4.3)	0 (0.0)	0 (0.0)	
T3	354 (2.6)	16 (4.2)	19 (1.8)	298 (2.6)	21 (7.0)	0 (0.0)	0 (0.0)	
T4	83 (0.6)	5 (1.3)	0 (0.0)	69 (0.6)	9 (3.0)	0 (0.0)	0 (0.0)	
Tx	2 (0.0)	0 (0.0)	2 (0.2)	0 (0.0)	0 (0.0)	0 (0.0)	0 (0.0)	
TX	2 (0.0)	0 (0.0)	0 (0.0)	2 (0.0)	0 (0.0)	0 (0.0)	0 (0.0)	
Missing data	4902 (35.7)	209 (54.4)	272 (26.3)	3594 (31.4)	252 (84.0)	452 (100.0)	123 (100.0)	
**N stage (%)**								**<0.001**
N0	5581 (40.6)	15 (3.9)	348 (33.6)	5212 (45.6)	6 (2.0)	0 (0.0)	0 (0.0)	

N1	2732 (19.9)	0 (0.0)	213 (20.6)	2494 (21.8)	25 (8.3)	0 (0.0)	0 (0.0)	
N2	969 (7.1)	0 (0.0)	85 (8.2)	867 (7.6)	17 (5.7)	0 (0.0)	0 (0.0)	
N3	124 (0.9)	0 (0.0)	59 (5.7)	65 (0.6)	0 (0.0)	0 (0.0)	0 (0.0)	
Missing data	4325 (31.5)	369 (96.1)	330 (31.9)	2799 (24.5)	252 (84.0)	452 (100.0)	123 (100.0)	
**ER status (%)**								**<0.001**
Positive	9108 (66.3)	74 (19.3)	624 (60.3)	8254 (72.2)	96 (32.0)	0 (0.0)	60 (48.8)	
Negative	2235 (16.3)	64 (16.7)	221 (21.4)	1833 (16.0)	81 (27.0)	0 (0.0)	36 (29.3)	
Missing data	2388 (17.4)	246 (64.1)	190 (18.4)	1350 (11.8)	123 (41.0)	452 (100.0)	27 (22.0)	
**PR status (%)**								**<0.001**
Positive	6752 (49.2)	50 (13.0)	499 (48.2)	6084 (53.2)	75 (25.0)	0 (0.0)	44 (35.8)	
Negative	3267 (23.8)	61 (15.9)	274 (26.5)	2778 (24.3)	102 (34.0)	0 (0.0)	52 (42.3)	
Missing data	3712 (27.0)	273 (71.1)	262 (25.3)	2575 (22.5)	123 (41.0)	452 (100.0)	27 (22.0)	
**HER2 status (%)**								**<0.001**
Positive	1689 (12.3)	13 (3.4)	221 (21.4)	1375 (12.0)	35 (11.7)	0 (0.0)	45 (36.6)	
Negative	9365 (68.2)	91 (23.7)	623 (60.2)	8460 (74.0)	140 (46.7)	0 (0.0)	51 (41.5)	
Missing data	2677 (19.5)	280 (72.9)	191 (18.5)	1602 (14.0)	125 (41.7)	452 (100.0)	27 (22.0)	
**TNBC (%)**	1173 (8.5)	69 (18.0)	9 (0.9)	1043 (9.1)	52 (17.3)	0 (0.0)	0 (0.0)	**<0.001**
**PAM50 subtype (%)**								**<0.001**
Basal	1999 (14.6)	132 (34.4)	171 (16.5)	1551 (13.6)	56 (18.7)	67 (14.8)	22 (17.9)	
Her2	1908 (13.9)	62 (16.1)	233 (22.5)	1458 (12.7)	55 (18.3)	74 (16.4)	26 (21.1)	
LumA	3980 (29.0)	63 (16.4)	231 (22.3)	3460 (30.3)	71 (23.7)	121 (26.8)	34 (27.6)	
LumB	5844 (42.6)	127 (33.1)	400 (38.6)	4968 (43.4)	118 (39.3)	190 (42.0)	41 (33.3)	
**Grade (%)**								**<0.001**
G1	1214 (8.8)	11 (2.9)	9 (0.9)	1180 (10.3)	2 (0.7)	5 (1.1)	7 (5.7)	
G2	4181 (30.4)	48 (12.5)	325 (31.4)	3695 (32.3)	45 (15.0)	18 (4.0)	50 (40.7)	
G3	3309 (24.1)	73 (19.0)	317 (30.6)	2808 (24.6)	59 (19.7)	12 (2.7)	40 (32.5)	
**CIBERSORTx scores (median [IQR])**								
B cells naive	0.06 [0.03, 0.10]	0.08 [0.03, 0.15]	0.08 [0.03, 0.15]	0.05 [0.03, 0.09]	0.07 [0.00, 0.12]	0.09 [0.05, 0.13]	0.09 [0.03, 0.13]	**<0.001**
B cells memory	0.00 [0.00, 0.02]	0.01 [0.00, 0.06]	0.01 [0.00, 0.06]	0.00 [0.00, 0.02]	0.07 [0.01, 0.13]	0.00 [0.00, 0.02]	0.00 [0.00, 0.04]	**<0.001**
Plasma cells	0.10 [0.04, 0.19]	0.13 [0.05, 0.30]	0.11 [0.05, 0.21]	0.09 [0.04, 0.17]	0.14 [0.08, 0.22]	0.19 [0.13, 0.29]	0.33 [0.09, 0.70]	**<0.001**

T cells CD8	0.12 [0.05, 0.20]	0.07 [0.00, 0.18]	0.11 [0.04, 0.21]	0.12 [0.06, 0.20]	0.23 [0.12, 0.34]	0.01 [0.00, 0.05]	0.07 [0.00, 0.21]	**<0.001**
T cells CD4 naive	0.00 [0.00, 0.00]	0.00 [0.00, 0.07]	0.00 [0.00, 0.00]	0.00 [0.00, 0.00]	0.00 [0.00, 0.12]	0.00 [0.00, 0.01]	0.00 [0.00, 0.00]	**<0.001**
T cells CD4 memory resting	0.33 [0.21, 0.43]	0.13 [0.00, 0.35]	0.46 [0.30, 0.65]	0.33 [0.24, 0.43]	0.06 [0.00, 0.22]	0.00 [0.00, 0.01]	0.38 [0.25, 0.55]	**<0.001**
T cells CD4 memory activated	0.00 [0.00, 0.03]	0.00 [0.00, 0.04]	0.00 [0.00, 0.01]	0.00 [0.00, 0.03]	0.05 [0.01, 0.13]	0.00 [0.00, 0.03]	0.02 [0.00, 0.07]	**<0.001**
T cells follicular helper	0.06 [0.03, 0.11]	0.21 [0.12, 0.30]	0.08 [0.03, 0.16]	0.06 [0.03, 0.09]	0.03 [0.01, 0.07]	0.45 [0.38, 0.53]	0.13 [0.08, 0.19]	**<0.001**
T cells regulatory (Tregs)	0.03 [0.00, 0.08]	0.07 [0.03, 0.12]	0.00 [0.00, 0.02]	0.04 [0.00, 0.08]	0.04 [0.00, 0.08]	0.02 [0.00, 0.05]	0.00 [0.00, 0.03]	**<0.001**
T cells gamma delta	0.00 [0.00, 0.04]	0.00 [0.00, 0.12]	0.00 [0.00, 0.00]	0.00 [0.00, 0.03]	0.00 [0.00, 0.07]	0.06 [0.01, 0.13]	0.17 [0.00, 0.39]	**<0.001**
NK cells resting	0.00 [0.00, 0.04]	0.04 [0.00, 0.10]	0.06 [0.01, 0.11]	0.00 [0.00, 0.03]	0.08 [0.02, 0.15]	0.03 [0.00, 0.06]	0.00 [0.00, 0.09]	**<0.001**
NK cells activated	0.04 [0.00, 0.08]	0.02 [0.00, 0.09]	0.01 [0.00, 0.04]	0.05 [0.01, 0.08]	0.00 [0.00, 0.05]	0.03 [0.00, 0.08]	0.00 [0.00, 0.05]	**<0.001**
Monocytes	0.04 [0.01, 0.07]	0.01 [0.00, 0.04]	0.03 [0.01, 0.07]	0.04 [0.01, 0.07]	0.06 [0.02, 0.12]	0.00 [0.00, 0.02]	0.00 [0.00, 0.09]	**<0.001**
Macrophages M0	0.19 [0.07, 0.35]	0.34 [0.18, 0.54]	0.26 [0.13, 0.43]	0.17 [0.06, 0.33]	0.13 [0.06, 0.28]	0.27 [0.18, 0.43]	0.24 [0.14, 0.47]	**<0.001**
Macrophages M1	0.13 [0.09, 0.18]	0.14 [0.09, 0.24]	0.13 [0.07, 0.23]	0.13 [0.09, 0.18]	0.08 [0.04, 0.17]	0.17 [0.11, 0.23]	0.16 [0.07, 0.30]	**<0.001**
Macrophages M2	0.51 [0.38, 0.66]	0.37 [0.23, 0.53]	0.47 [0.36, 0.61]	0.54 [0.41, 0.68]	0.16 [0.09, 0.29]	0.26 [0.20, 0.34]	0.50 [0.27, 0.69]	**<0.001**
Dendritic cells resting	0.02 [0.00, 0.05]	0.00 [0.00, 0.04]	0.00 [0.00, 0.01]	0.02 [0.01, 0.05]	0.03 [0.00, 0.10]	0.05 [0.03, 0.09]	0.02 [0.00, 0.04]	**<0.001**
Dendritic cells activated	0.00 [0.00, 0.01]	0.01 [0.00, 0.04]	0.01 [0.00, 0.04]	0.00 [0.00, 0.00]	0.02 [0.00, 0.04]	0.03 [0.00, 0.06]	0.00 [0.00, 0.04]	**<0.001**
Mast cells resting	0.15 [0.08, 0.26]	0.10 [0.04, 0.18]	0.10 [0.06, 0.16]	0.17 [0.10, 0.28]	0.07 [0.00, 0.15]	0.00 [0.00, 0.06]	0.11 [0.02, 0.19]	**<0.001**
Mast cells activated	0.00 [0.00, 0.02]	0.00 [0.00, 0.02]	0.00 [0.00, 0.00]	0.00 [0.00, 0.01]	0.01 [0.00, 0.08]	0.11 [0.06, 0.16]	0.05 [0.00, 0.13]	**<0.001**
Eosinophils	0.00 [0.00, 0.00]	0.00 [0.00, 0.00]	0.00 [0.00, 0.00]	0.00 [0.00, 0.00]	0.00 [0.00, 0.00]	0.00 [0.00, 0.01]	0.00 [0.00, 0.00]	**<0.001**
Neutrophils	0.02 [0.00, 0.03]	0.00 [0.00, 0.01]	0.00 [0.00, 0.01]	0.02 [0.01, 0.04]	0.01 [0.00, 0.08]	0.01 [0.00, 0.02]	0.00 [0.00, 0.01]	**<0.001**

Abbreviations:ER, estrogen receptor; PR, progesterone receptor; TNBC, triple-negative breast cancer. P-values were calculated using Chi-square test for categorical variables (with continuity correction) and ANOVA for continuous variables.

Supplementary Table 2

Clinicopathological characteristics of the 13,731 breast cancer samples included in the study, stratified by immune phenotype

Characteristic	Overall (n = 13731)	Hot (n = 4260)	Moderate (n = 4094)	Cold (n = 5377)	p
**Ancestry**					**0.001**
African	384 (2.8)	140 (3.3)	110 (2.7)	134 (2.5)	
East Asian	1035 (7.5)	366 (8.6)	298 (7.3)	371 (6.9)	
European	11437 (83.3)	3495 (82.0)	3417 (83.5)	4525 (84.2)	
Hispanic	300 (2.2)	109 (2.6)	83 (2.0)	108 (2.0)	
Southeast Asian	452 (3.3)	111 (2.6)	149 (3.6)	192 (3.6)	
West Asian	123 (0.9)	39 (0.9)	37 (0.9)	47 (0.9)	
**Immune score**	-0.03 (0.42)	0.49 (0.14)	-0.01 (0.17)	-0.45 (0.12)	**<0.001**
**Age group (%)**					**<0.001**
<40 years	583 (4.2)	216 (5.1)	157 (3.8)	210 (3.9)	
40-49 years	1538 (11.2)	521 (12.2)	458 (11.2)	559 (10.4)	
50-64 years	3739 (27.2)	1232 (28.9)	1112 (27.2)	1395 (25.9)	
65-74 years	3036 (22.1)	933 (21.9)	904 (22.1)	1199 (22.3)	
≥75 years	2646 (19.3)	729 (17.1)	794 (19.4)	1123 (20.9)	
Missing data	2189 (15.9)	629 (14.8)	669 (16.3)	891 (16.6)	
**T stage (%)**					0.172
T1	4923 (35.9)	1531 (35.9)	1466 (35.8)	1926 (35.8)	
T1a	3 (0.0)	1 (0.0)	0 (0.0)	2 (0.0)	
T1b	33 (0.2)	12 (0.3)	8 (0.2)	13 (0.2)	
T1c	272 (2.0)	100 (2.3)	77 (1.9)	95 (1.8)	
T2	3156 (23.0)	1037 (24.3)	918 (22.4)	1201 (22.3)	
T3	354 (2.6)	115 (2.7)	107 (2.6)	132 (2.5)	
T4	83 (0.6)	31 (0.7)	22 (0.5)	30 (0.6)	
Tx	2 (0.0)	1 (0.0)	1 (0.0)	0 (0.0)	
TX	2 (0.0)	1 (0.0)	0 (0.0)	1 (0.0)	
Missing data	4902 (35.7)	1431 (33.6)	1495 (36.5)	1976 (36.8)	
**N stage (%)**					**<0.001**
N0	5581 (40.6)	1720 (40.4)	1611 (39.4)	2250 (41.8)	
N1	2732 (19.9)	796 (18.7)	826 (20.2)	1110 (20.6)	
N2	969 (7.1)	349 (8.2)	299 (7.3)	321 (6.0)	
N3	124 (0.9)	49 (1.2)	35 (0.9)	40 (0.7)	
Missing data	4325 (31.5)	1346 (31.6)	1323 (32.3)	1656 (30.8)	
**ER status (%)**					**<0.001**
Positive	9108 (66.3)	2345 (55.0)	2738 (66.9)	4025 (74.9)	
Negative	2235 (16.3)	1226 (28.8)	621 (15.2)	388 (7.2)	
Missing data	2388 (17.4)	689 (16.2)	735 (18.0)	964 (17.9)	
**PR status (%)**					**<0.001**
Positive	6752 (49.2)	1698 (39.9)	2040 (49.8)	3014 (56.1)	
Negative	3267 (23.8)	1457 (34.2)	927 (22.6)	883 (16.4)	
Missing data	3712 (27.0)	1105 (25.9)	1127 (27.5)	1480 (27.5)	
**HER2 status (%)**					**<0.001**
Positive	1689 (12.3)	721 (16.9)	527 (12.9)	441 (8.2)	
Negative	9365 (68.2)	2745 (64.4)	2754 (67.3)	3866 (71.9)	
Missing data	2677 (19.5)	794 (18.6)	813 (19.9)	1070 (19.9)	

**TNBC (%)**	1173 (8.5)	654 (15.4)	311 (7.6)	208 (3.9)	**<0.001**
**PAM50 subtype (%)**					**<0.001**
Basal	1999 (14.6)	1147 (26.9)	509 (12.4)	343 (6.4)	
Her2	1908 (13.9)	943 (22.1)	582 (14.2)	383 (7.1)	
LumA	3980 (29.0)	761 (17.9)	1286 (31.4)	1933 (35.9)	
LumB	5844 (42.6)	1409 (33.1)	1717 (41.9)	2718 (50.5)	
**Grade (%)**					**<0.001**
G1	1214 (8.8)	211 (5.0)	393 (9.6)	610 (11.3)	
G2	4181 (30.4)	987 (23.2)	1270 (31.0)	1924 (35.8)	
G3	3309 (24.1)	1458 (34.2)	918 (22.4)	933 (17.4)	
**CIBERSORTx score (median [IQR])**					
B cells naive	0.06 [0.03, 0.10]	0.06 [0.03, 0.12]	0.05 [0.03, 0.10]	0.05 [0.03, 0.09]	**<0.001**
B cells memory	0.00 [0.00, 0.02]	0.00 [0.00, 0.04]	0.00 [0.00, 0.02]	0.00 [0.00, 0.01]	**<0.001**
Plasma cells	0.10 [0.04, 0.19]	0.11 [0.05, 0.20]	0.09 [0.04, 0.17]	0.10 [0.04, 0.18]	**<0.001**
T cells CD8	0.12 [0.05, 0.20]	0.20 [0.11, 0.29]	0.11 [0.04, 0.18]	0.08 [0.03, 0.14]	**<0.001**
T cells CD4 naive	0.00 [0.00, 0.00]	0.00 [0.00, 0.00]	0.00 [0.00, 0.00]	0.00 [0.00, 0.00]	**0.039**
T cells CD4 memory resting	0.33 [0.21, 0.43]	0.34 [0.22, 0.48]	0.34 [0.24, 0.45]	0.31 [0.20, 0.40]	**<0.001**
T cells CD4 memory activated	0.00 [0.00, 0.03]	0.02 [0.00, 0.07]	0.00 [0.00, 0.02]	0.00 [0.00, 0.01]	**<0.001**
T cells follicular helper	0.06 [0.03, 0.11]	0.07 [0.03, 0.12]	0.06 [0.03, 0.11]	0.06 [0.04, 0.10]	**<0.001**
T cells regulatory (Tregs)	0.03 [0.00, 0.08]	0.04 [0.00, 0.10]	0.03 [0.00, 0.08]	0.03 [0.00, 0.07]	**<0.001**
T cells gamma delta	0.00 [0.00, 0.04]	0.00 [0.00, 0.06]	0.00 [0.00, 0.05]	0.00 [0.00, 0.02]	**<0.001**
NK cells resting	0.00 [0.00, 0.04]	0.00 [0.00, 0.04]	0.00 [0.00, 0.03]	0.01 [0.00, 0.04]	**<0.001**
NK cells activated	0.04 [0.00, 0.08]	0.04 [0.00, 0.08]	0.04 [0.00, 0.08]	0.04 [0.00, 0.07]	**<0.001**
Monocytes	0.04 [0.01, 0.07]	0.04 [0.01, 0.07]	0.04 [0.01, 0.08]	0.03 [0.01, 0.07]	**<0.001**
Macrophages M0	0.19 [0.07, 0.35]	0.21 [0.10, 0.36]	0.19 [0.06, 0.35]	0.17 [0.05, 0.35]	**<0.001**
Macrophages M1	0.13 [0.09, 0.18]	0.17 [0.12, 0.23]	0.13 [0.09, 0.18]	0.10 [0.08, 0.14]	**<0.001**
Macrophages M2	0.51 [0.38, 0.66]	0.45 [0.33, 0.58]	0.53 [0.40, 0.65]	0.57 [0.41, 0.71]	**<0.001**
Dendritic cells resting	0.02 [0.00, 0.05]	0.02 [0.00, 0.05]	0.02 [0.01, 0.05]	0.02 [0.01, 0.05]	**<0.001**
Dendritic cells activated	0.00 [0.00, 0.01]	0.00 [0.00, 0.01]	0.00 [0.00, 0.01]	0.00 [0.00, 0.01]	**<0.001**
Mast cells resting	0.15 [0.08, 0.26]	0.12 [0.07, 0.19]	0.16 [0.08, 0.26]	0.19 [0.10, 0.31]	**<0.001**
Mast cells activated	0.00 [0.00, 0.02]	0.00 [0.00, 0.00]	0.00 [0.00, 0.03]	0.00 [0.00, 0.03]	**<0.001**
Eosinophils	0.00 [0.00, 0.00]	0.00 [0.00, 0.00]	0.00 [0.00, 0.00]	0.00 [0.00, 0.00]	**<0.001**
Neutrophils	0.02 [0.00, 0.03]	0.01 [0.00, 0.03]	0.02 [0.00, 0.04]	0.02 [0.00, 0.04]	**<0.001**

Abbreviations: ER, estrogen receptor; PR, progesterone receptor; TNBC, triple-negative breast cancer. P-values were calculated using Chi-square test for categorical variables (with continuity correction) and ANOVA for continuous variables.

Supplementary Table 3

Clinicopathological characteristics of the 13,731 breast cancer samples included in the study, stratified by fibroblast cluster

Characteristic	Overall (n = 13731)	iCAF (n = 7130)	myCAF (n = 4950)	NF (n = 1651)	p
**Ancestry (%)**					**<0.001**
African	384 (2.8)	182 (2.6)	174 (3.5)	28 (1.7)	
East Asian	1035 (7.5)	179 (2.5)	337 (6.8)	519 (31.4)	
European	11437 (83.3)	6534 (91.6)	4042 (81.7)	861 (52.2)	
Hispanic	300 (2.2)	155 (2.2)	112 (2.3)	33 (2.0)	
Southeast Asian	452 (3.3)	7 (0.1)	257 (5.2)	188 (11.4)	
West Asian	123 (0.9)	73 (1.0)	28 (0.6)	22 (1.3)	
**Immune Score (mean (SD))**	-0.03 (0.42)	-0.05 (0.43)	0.01 (0.39)	-0.01 (0.43)	**<0.001**
**Immune phenotype (%)**					**<0.001**
Hot	4260 (31.0)	2153 (30.2)	1571 (31.7)	536 (32.5)	
Moderate	4094 (29.8)	1937 (27.2)	1700 (34.3)	457 (27.7)	
Cold	5377 (39.2)	3040 (42.6)	1679 (33.9)	658 (39.9)	
**Age (mean (SD))**	62.00 (13.99)	63.63 (14.19)	60.47 (13.59)	59.23 (13.34)	**<0.001**
**Age group (%)**					**<0.001**
<40 years	583 (4.2)	287 (4.0)	216 (4.4)	80 (4.8)	
40-49 years	1538 (11.2)	680 (9.5)	602 (12.2)	256 (15.5)	
50-64 years	3739 (27.2)	1764 (24.7)	1458 (29.5)	517 (31.3)	
65-74 years	3036 (22.1)	1704 (23.9)	1033 (20.9)	299 (18.1)	
≥75 years	2646 (19.3)	1698 (23.8)	733 (14.8)	215 (13.0)	
Missing data	2189 (15.9)	997 (14.0)	908 (18.3)	284 (17.2)	
**T stage (%)**					**<0.001**
T1	4923 (35.9)	2663 (37.3)	1850 (37.4)	410 (24.8)	
T1a	3 (0.0)	0 (0.0)	0 (0.0)	3 (0.2)	
T1b	33 (0.2)	0 (0.0)	16 (0.3)	17 (1.0)	
T1c	272 (2.0)	0 (0.0)	85 (1.7)	187 (11.3)	
T2	3156 (23.0)	1392 (19.5)	1235 (24.9)	529 (32.1)	
T3	354 (2.6)	192 (2.7)	140 (2.8)	22 (1.3)	
T4	83 (0.6)	43 (0.6)	32 (0.6)	8 (0.5)	
Tx	2 (0.0)	0 (0.0)	1 (0.0)	1 (0.1)	
TX	2 (0.0)	0 (0.0)	2 (0.0)	0 (0.0)	
Missing data	4902 (35.7)	2840 (39.8)	1589 (32.1)	473 (28.7)	
**N stage (%)**					**<0.001**
N0	5581 (40.6)	3046 (42.7)	1902 (38.4)	633 (38.3)	
N1	2732 (19.9)	1469 (20.6)	909 (18.4)	354 (21.4)	
N2	969 (7.1)	533 (7.5)	305 (6.2)	131 (7.9)	
N3	124 (0.9)	34 (0.5)	43 (0.9)	47 (2.8)	
Missing data	4325 (31.5)	2048 (28.7)	1791 (36.2)	486 (29.4)	
**ER status (%)**					**<0.001**
Positive	9108 (66.3)	4766 (66.8)	3305 (66.8)	1037 (62.8)	
Negative	2235 (16.3)	1245 (17.5)	663 (13.4)	327 (19.8)	
Missing data	2388 (17.4)	1119 (15.7)	982 (19.8)	287 (17.4)	

**PR status (%)**					**<0.001**
Positive	6752 (49.2)	3612 (50.7)	2437 (49.2)	703 (42.6)	
Negative	3267 (23.8)	1907 (26.7)	931 (18.8)	429 (26.0)	
Missing data	3712 (27.0)	1611 (22.6)	1582 (32.0)	519 (31.4)	
**HER2 status (%)**					**<0.001**
Positive	1689 (12.3)	889 (12.5)	582 (11.8)	218 (13.2)	
Negative	9365 (68.2)	5017 (70.4)	3258 (65.8)	1090 (66.0)	
Missing data	2677 (19.5)	1224 (17.2)	1110 (22.4)	343 (20.8)	
**TNBC (%)**	1173 (8.5)	773 (10.8)	313 (6.3)	87 (5.3)	**<0.001**
**PAM50 subtype (%)**					**<0.001**
Basal	1999 (14.6)	1132 (15.9)	577 (11.7)	290 (17.6)	
Her2	1908 (13.9)	891 (12.5)	782 (15.8)	235 (14.2)	
LumA	3980 (29.0)	1604 (22.5)	1729 (34.9)	647 (39.2)	
LumB	5844 (42.6)	3503 (49.1)	1862 (37.6)	479 (29.0)	
**Grade (%)**					**<0.001**
G1	1214 (8.8)	546 (7.7)	559 (11.3)	109 (6.6)	
G2	4181 (30.4)	2324 (32.6)	1377 (27.8)	480 (29.1)	
G3	3309 (24.1)	2064 (28.9)	896 (18.1)	349 (21.1)	
**CIBERSORTx scores (median [IQR])**					
B cells naive	0.06 [0.03, 0.10]	0.05 [0.03, 0.09]	0.06 [0.03, 0.10]	0.08 [0.04, 0.14]	**<0.001**
B cells memory	0.00 [0.00, 0.02]	0.00 [0.00, 0.02]	0.00 [0.00, 0.01]	0.00 [0.00, 0.05]	**<0.001**
Plasma cells	0.10 [0.04, 0.19]	0.09 [0.04, 0.18]	0.10 [0.04, 0.18]	0.13 [0.07, 0.23]	**<0.001**
T cells CD8	0.12 [0.05, 0.20]	0.12 [0.05, 0.21]	0.11 [0.04, 0.19]	0.12 [0.04, 0.20]	**<0.001**
T cells CD4 naive	0.00 [0.00, 0.00]	0.00 [0.00, 0.00]	0.00 [0.00, 0.00]	0.00 [0.00, 0.00]	**<0.001**
T cells CD4 memory resting	0.33 [0.21, 0.43]	0.33 [0.23, 0.44]	0.32 [0.20, 0.42]	0.33 [0.17, 0.50]	**<0.001**
T cells CD4 memory activated	0.00 [0.00, 0.03]	0.00 [0.00, 0.04]	0.00 [0.00, 0.02]	0.00 [0.00, 0.02]	**<0.001**
T cells follicular helper	0.06 [0.03, 0.11]	0.06 [0.03, 0.10]	0.06 [0.03, 0.13]	0.06 [0.02, 0.15]	**<0.001**
T cells regulatory (Tregs)	0.03 [0.00, 0.08]	0.03 [0.00, 0.08]	0.04 [0.00, 0.08]	0.01 [0.00, 0.06]	**<0.001**
T cells gamma delta	0.00 [0.00, 0.04]	0.00 [0.00, 0.05]	0.00 [0.00, 0.03]	0.00 [0.00, 0.02]	**<0.001**
NK cells resting	0.00 [0.00, 0.04]	0.00 [0.00, 0.03]	0.00 [0.00, 0.04]	0.03 [0.00, 0.09]	**<0.001**
NK cells activated	0.04 [0.00, 0.08]	0.04 [0.01, 0.08]	0.04 [0.00, 0.08]	0.03 [0.00, 0.07]	**<0.001**
Monocytes	0.04 [0.01, 0.07]	0.04 [0.01, 0.08]	0.03 [0.00, 0.06]	0.05 [0.02, 0.09]	**<0.001**
Macrophages M0	0.19 [0.07, 0.35]	0.15 [0.05, 0.30]	0.25 [0.11, 0.42]	0.20 [0.07, 0.36]	**<0.001**
Macrophages M1	0.13 [0.09, 0.18]	0.14 [0.10, 0.19]	0.12 [0.09, 0.17]	0.11 [0.07, 0.17]	**<0.001**
Macrophages M2	0.51 [0.38, 0.66]	0.53 [0.39, 0.68]	0.52 [0.38, 0.65]	0.43 [0.30, 0.58]	**<0.001**
Dendritic cells resting	0.02 [0.00, 0.05]	0.02 [0.01, 0.05]	0.02 [0.00, 0.04]	0.01 [0.00, 0.04]	**<0.001**
Dendritic cells activated	0.00 [0.00, 0.01]	0.00 [0.00, 0.00]	0.00 [0.00, 0.01]	0.00 [0.00, 0.03]	**<0.001**
Mast cells resting	0.15 [0.08, 0.26]	0.14 [0.08, 0.24]	0.18 [0.10, 0.29]	0.11 [0.05, 0.22]	**<0.001**
Mast cells activated	0.00 [0.00, 0.02]	0.00 [0.00, 0.02]	0.00 [0.00, 0.02]	0.00 [0.00, 0.04]	**0.002**
Eosinophils	0.00 [0.00, 0.00]	0.00 [0.00, 0.00]	0.00 [0.00, 0.00]	0.00 [0.00, 0.00]	**<0.001**
Neutrophils	0.02 [0.00, 0.03]	0.02 [0.01, 0.04]	0.02 [0.00, 0.03]	0.00 [0.00, 0.02]	**<0.001**

Abbreviations: ER, estrogen receptor; PR, progesterone receptor; TNBC, triple-negative breast cancer. P-values were calculated using Chi-square test for categorical variables (with continuity correction) and ANOVA for continuous variables.

Supplementary Table 4

Transcriptomic datasets (n = 22) included in the study

Dataset number	Dataset name	Data source	Data type	Sequencing and microarray platform	Population	Number of patients	Treatment
**Treatment naive**							
1	GSE20486/ GSE97177	https://www.ncbi.nlm.nih.gov/gds/?term=GSE20486[Accession] https://www.ncbi.nlm.nih.gov/geo/query/acc.cgi?acc=GSE97177	RNA microarray	Illumina HumanHT-12 V3.0 expression beadchip	Sweden	150	Untreated

2	GSE65194	Gene Expression Omnibus, https://www.ncbi.nlm.nih.gov/geo/query/acc.cgi?acc=GSE65194	RNA microarrray	[HG-U133_Plus_2] Affymetrix Human Genome U133 Plus 2.0 Array	France	130 breast cancer samples (41 TNBC ; 30 Her2 ; 30 Luminal B and 29 Luminal A), 11 normal breast tissue samples and 14 TNBC cell lines
3	TCGA	Hub: https://tcga.xenahubs.net	RNA-seq	IlluminaHiSeq_RNASeqV2	USA	1247
4	METABRIC	https://www.cbioportal.org/study/summary?id=brca_metabric	RNA microarray	Illumina HT-12 v3 platforms	CANADA-UK	2509

		https://www.kaggle.com/datasets/raghadalharbi/breast-cancer-gene-expression-profiles-metabric				
5	GSE96058-SCAN-B	Gene Expression Omnibus, https://www.ncbi.nlm.nih.gov/gds/?term=GSE96058[Accession] https://data.mendeley.com/datasets/yzxtxn4nmd/3,	RNA-seq	Illumina HiSeq 2000 (Homo sapiens) Illumina NextSeq 500 (Homo sapiens)	SWEDEN	9206
6	GSE15852	Gene Expression Omnibus, https://www.ncbi.nlm.nih.gov/gds/?term=GSE15852[Accession]	RNA microarray	[HG-U133A] Affymetrix Human Genome U133A Array	MALAYSIA	86 paired (normal/tumor) samples from 43 patients

						29, 10 and 4 were from Malay, Chinese and Indian patients, respectively	
7	GSE20194	[Gene Expression Omnibus, https://www.ncbi.nlm.nih.gov/geo/query/acc.cgi?acc=GSE20194](https://www.ncbi.nlm.nih.gov/geo/query/acc.cgi?acc=GSE20194)	RNA microarray	Affymetrix Human Genome U133A Array	USA	230	Patients received 6 months of preoperative (neoadjuvant) chemotherapy including paclitaxel, 5-fluorouracil, cyclophosphamide and doxorubicin followed by surgical resection of the cancer

8	GSE20271	Gene Expression Omnibus, https://www.ncbi.nlm.nih.gov/gds/?term=GSE20271[Accession]	RNA microarray	[HG-U133A] Affymetrix Human Genome U133A Array	Peru (n=79)<br>Spain (n=60)<br>Mexico (n=19)<br>*USA=115*	273

9	GSE37751	Gene Expression Omnibus, https://www.ncbi.nlm.nih.gov/geo/query/acc.cgi?acc=GSE37751	RNA microarray	[HuGene-1_0-st] Affymetrix Human Gene 1.0 ST Array [transcript (gene) version]	USA	108 paired (normal/tumor) samples
10	GSE78958	Gene Expression Omnibus, https://www.ncbi.nlm.nih.gov/geo/query/acc.cgi?a cc=GSE78958	RNA microarray	[HG-U133A_2] Affymetrix Human Genome U133A 2.0 Array	USA	406 grouped by BMI
11	GSE48390	Gene Expression Omnibus, https://www.ncbi.nlm.nih.gov/geo/query/acc.cgi?acc=GSE48390	RNA microarray	[HG-U133_Plus_2] Affymetrix Human Genome U133 Plus 2.0 Array	Taiwan	81

12	GSE54002	Gene Expression Omnibus, https://www.ncbi.nlm.nih.gov/geo/query/acc.cgi?acc=GSE54002	RNA microarray	[HG-U133_Plus_2] Affymetrix Human Genome U133 Plus 2.0 Array	Singapore	433 (417 tumor and 16 non-tumor samples)
13	GSE2109	Gene Expression Omnibus, https://www.ncbi.nlm.nih.gov/geo/query/acc.cgi?acc=GSE2109	RNA microarray	[HG-U133_Plus_2] Affymetrix Human Genome U133 Plus 2.0 Array	USA	2158
14	GSE75678	Gene Expression Omnibus,	RNA microarray	Agilent-014850 Whole Human Genome Microarray 4x44K	USA-Mexican	54 tumor samples of mexican patients

		https://www.ncbi.nlm.nih.gov/geo/query/acc.cgi?acc=GSE75678		G4112F (Feature Number version)			with breast cancer. Samples obtained from the Hospital San Jose Tec de Monterrey
15	GSE86374	Gene Expression Omnibus, https://www.ncbi.nlm.nih.gov/geo/query/acc.cgi?acc=GSE86374	RNA microarray	GPL6244	[HuGene-1_0-st] Affymetrix Human Gene 1.0 ST Array [transcript (gene) version]	Mexican-Hispanic	159 breast tumors in Mexican or Hispanic populations

16	RA_QA	Figshare, https://figshare.com/articles/dataset/Breast_cancer_Qatar_dataset_RA-QA/12901928<br>https://www.nature.com/articles/s41523-021-00215-x?fromPaywallRec=false#data-availability	RNA-seq	Illumina HiSeq2500 platform (Illumina) with paired-end 25× coverage (PE100–125)	Middle Eastern/Asian	24
17	GSE113184	Gene Expression Omnibus, https://www.ncbi.nlm.nih.gov/geo/download/?acc=GSE113184	RNA-Seq	GPL24896	ASIAN	178 Korean breast cancer cohort (SMC)

	SMC	https://pmc.ncbi.nlm.nih.gov/articles/PMC5928087/?report=classic#sec24		Illumina hiseq 2500 (Homo sapiens)			
18	GSE211167	Gene Expression Omnibus, https://www.ncbi.nlm.nih.gov/geo/download/?acc=GSE211167	RNA-seq	GPL18573 Illumina NextSeq 500 (Homo sapiens)	AAs, West and East Africans with TNBC	27	
19	CBCGA	https://ngdc.cncb.ac.cn/bioproject/browse/PRJCA017539 https://www.nature.com/articles/s43018-024-00725-0#Sec38,	RNA-seq	HiSeq X Ten	CHINA	705	Adjuvant chemotherapy, Target therapy usage, Adjuvant radiotherapy, Adjuvant endocrine therapy.

20	GSE29044	Gene Expression Omnibus, https://www.ncbi.nlm.nih.gov/geo/query/acc.cgi?acc=GSE29044	RNA microarrray	GPL570	[HG-U133_Plus_2] Affymetrix Human Genome U133 Plus 2.0 Array	Middle East	RNA expression profile from tumor (n=73) and adjacent disease free tissues (n=36) microarray	Pre-treatment
21	GSE36295	Gene Expression Omnibus,	RNA microarrray	GPL6244	[HuGene-1_0-st] Affymetrix Human	Saudi Arabia	45 surgically resected breast cancer tissues and 8 healthy breast tissues	Naive

		https://www.ncbi.nlm.nih.gov/geo/query/acc.cgi?acc=GSE36295		Gene 1.0 ST Array [transcript (gene) version]			
22	GSE12093	Gene Expression Omnibus, https://www.ncbi.nlm.nih.gov/gds/?term=GSE12093[Accession] https://www.ncbi.nlm.nih.gov/pubmed/18821012	RNA microarrray	GPL96 [HG-U133A] Affymetrix Human Genome U133A Array	Slovenia, Italy, Germany, USA	136 breast cancer samples that were treated with tamoxifen	Treatment naive samples GE profiled, then treated with tamoxifen

Supplementary Table 5

Clinicopathological characteristics of the 13,731 breast cancer samples included in the study, stratified by dataset

	Overall (n = 13731)	CBCGA (n = 705)	GSE113184_SMC (n = 165)	GSE12093 (n = 118)	GSE15852_Malaysian (n = 35)	GSE20194_MAQCII (n = 258)	GSE20271_TFAC (n = 168)	GSE2109_IGC (n = 308)	GSE211167_Sub_Saharan (n = 26)	GSE29044_Saudi_Arabia (n = 67)	GSE36295_Saudi_Arabia (n = 40)	GSE37751_Baltimore (n = 51)	GSE48390_Taiwan (n = 72)	GSE54002_Singapore (n = 417)	GSE65194_Curie (n = 116)	GSE75678_Mexican (n = 48)	GSE78958_Obese (n = 413)	GSE86374_Mexican (n = 117)	METABRIC (n = 1227)	Project8p (n= 137)	RA_QA_Qatar (n = 20)	SCANB (n = 8258)	TCGA (n = 965)
**Ancestry (%)**																							
African	384 (2.8)	0 (0.0)	0 (0.0)	0 (0.0)	0 (0.0)	29 (11.2)	12 (7.1)	16 (5.2)	26 (100.0)	0 (0.0)	0 (0.0)	27 (52.9)	0 (0.0)	0 (0.0)	0 (0.0)	0 (0.0)	95 (23.0)	0 (0.0)	0 (0.0)	0 (0.0)	0 (0.0)	0 (0.0)	179 (18.5)
East Asian	1035 (7.5)	705 (100.0)	165 (100.0)	0 (0.0)	0 (0.0)	18 (7.0)	1 (0.6)	2 (0.6)	0 (0.0)	0 (0.0)	0 (0.0)	0 (0.0)	72 (100.0)	0 (0.0)	0 (0.0)	0 (0.0)	10 (2.4)	0 (0.0)	0 (0.0)	0 (0.0)	4 (20.0)	0 (0.0)	58 (6.0)
European	11437 (83.3)	0 (0.0)	0 (0.0)	118 (100.0)	0 (0.0)	169 (65.5)	72 (42.9)	286 (92.9)	0 (0.0)	0 (0.0)	0 (0.0)	24 (47.1)	0 (0.0)	0 (0.0)	116 (100.0)	0 (0.0)	302 (73.1)	0 (0.0)	1227 (100.0)	137 (100.0)	0 (0.0)	8258 (100.0)	728 (75.4)
Hispanic	300 (2.2)	0 (0.0)	0 (0.0)	0 (0.0)	0 (0.0)	42 (16.3)	83 (49.4)	4 (1.3)	0 (0.0)	0 (0.0)	0 (0.0)	0 (0.0)	0 (0.0)	0 (0.0)	0 (0.0)	48 (100.0)	6 (1.5)	117 (100.0)	0 (0.0)	0 (0.0)	0 (0.0)	0 (0.0)	0 (0.0)
Southeast Asian	452 (3.3)	0 (0.0)	0 (0.0)	0 (0.0)	35 (100.0)	0 (0.0)	0 (0.0)	0 (0.0)	0 (0.0)	0 (0.0)	0 (0.0)	0 (0.0)	0 (0.0)	417 (100.0)	0 (0.0)	0 (0.0)	0 (0.0)	0 (0.0)	0 (0.0)	0 (0.0)	0 (0.0)	0 (0.0)	0 (0.0)
West Asian	123 (0.9)	0 (0.0)	0 (0.0)	0 (0.0)	0 (0.0)	0 (0.0)	0 (0.0)	0 (0.0)	0 (0.0)	67 (100.0)	40 (100.0)	0 (0.0)	0 (0.0)	0 (0.0)	0 (0.0)	0 (0.0)	0 (0.0)	0 (0.0)	0 (0.0)	0 (0.0)	16 (80.0)	0 (0.0)	0 (0.0)
**Immune phenotype (%)**																							
Hot	4260 (31.0)	259 (36.7)	55 (33.3)	22 (18.6)	12 (34.3)	71 (27.5)	63 (37.5)	88 (28.6)	7 (26.9)	20 (29.9)	16 (40.0)	27 (52.9)	23 (31.9)	99 (23.7)	25 (21.6)	16 (33.3)	108 (26.2)	48 (41.0)	369 (30.1)	46 (33.6)	4 (20.0)	2546 (30.8)	336 (34.8)
Moderate	4094 (29.8)	193 (27.4)	50 (30.3)	36 (30.5)	12 (34.3)	87 (33.7)	40 (23.8)	88 (28.6)	8 (30.8)	18 (26.9)	10 (25.0)	6 (11.8)	26 (36.1)	137 (32.9)	30 (25.9)	16 (33.3)	126 (30.5)	32 (27.4)	371 (30.2)	45 (32.8)	10 (50.0)	2468 (29.9)	285 (29.5)
Cold	5377 (39.2)	253 (35.9)	60 (36.4)	60 (50.8)	11 (31.4)	100 (38.8)	65 (38.7)	132 (42.9)	11 (42.3)	29 (43.3)	14 (35.0)	18 (35.3)	23 (31.9)	181 (43.4)	61 (52.6)	16 (33.3)	179 (43.3)	37 (31.6)	487 (39.7)	46 (33.6)	6 (30.0)	3244 (39.3)	344 (35.6)
**Immune score (mean (SD))**	-0.03 (0.42)	-0.02 (0.48)	-0.01 (0.45)	-0.03 (0.32)	-0.01 (0.20)	-0.03 (0.37)	-0.01 (0.35)	-0.02 (0.34)	-0.01 (0.43)	0.01 (0.37)	0.05 (0.45)	0.09 (0.46)	-0.01 (0.36)	-0.02 (0.34)	-0.02 (0.37)	-0.02 (0.39)	-0.02 (0.38)	0.03 (0.41)	-0.02 (0.39)	-0.03 (0.38)	-0.09 (0.30)	-0.03 (0.43)	-0.01 (0.45)
**Age group (%)**																							
<40 years	583 (4.2)	63 (8.9)	103 (62.4)	0 (0.0)	5 (14.3)	32 (12.4)	24 (14.3)	0 (0.0)	0 (0.0)	0 (0.0)	10 (25.0)	10 (19.6)	0 (0.0)	0 (0.0)	15 (12.9)	4 (8.3)	0 (0.0)	0 (0.0)	77 (6.3)	11 (8.0)	2 (10.0)	163 (2.0)	64 (6.6)
40-49 years	1538 (11.2)	213 (30.2)	33 (20.0)	0 (0.0)	14 (40.0)	77 (29.8)	64 (38.1)	0 (0.0)	0 (0.0)	0 (0.0)	18 (45.0)	13 (25.5)	0 (0.0)	0 (0.0)	28 (24.1)	20 (41.7)	0 (0.0)	0 (0.0)	174 (14.2)	27 (19.7)	8 (40.0)	712 (8.6)	137 (14.2)
50-64 years	3739 (27.2)	336 (47.7)	25 (15.2)	0 (0.0)	12 (34.3)	111 (43.0)	57 (33.9)	0 (0.0)	0 (0.0)	0 (0.0)	8 (20.0)	12 (23.5)	0 (0.0)	0 (0.0)	56 (48.3)	20 (41.7)	0 (0.0)	0 (0.0)	471 (38.4)	44 (32.1)	10 (50.0)	2259 (27.4)	318 (33.0)
65-74 years	3036 (22.1)	71 (10.1)	0 (0.0)	0 (0.0)	2 (5.7)	33 (12.8)	23 (13.7)	0 (0.0)	0 (0.0)	0 (0.0)	3 (7.5)	8 (15.7)	0 (0.0)	0 (0.0)	11 (9.5)	3 (6.2)	0 (0.0)	0 (0.0)	330 (26.9)	28 (20.4)	0 (0.0)	2412 (29.2)	112 (11.6)
≥75 years	2646 (19.3)	22 (3.1)	0 (0.0)	0 (0.0)	2 (5.7)	5 (1.9)	0 (0.0)	0 (0.0)	0 (0.0)	0 (0.0)	1 (2.5)	8 (15.7)	0 (0.0)	0 (0.0)	6 (5.2)	1 (2.1)	0 (0.0)	0 (0.0)	175 (14.3)	26 (19.0)	0 (0.0)	2323 (28.1)	77 (8.0)
Not available	2189 (15.9)	0 (0.0)	4 (2.4)	118 (100.0)	0 (0.0)	0 (0.0)	0 (0.0)	308 (100.0)	26 (100.0)	67 (100.0)	0 (0.0)	0 (0.0)	72 (100.0)	417 (100.0)	0 (0.0)	0 (0.0)	413 (100.0)	117 (100.0)	0 (0.0)	1 (0.7)	0 (0.0)	389 (4.7)	257 (26.6)
**Stage (%)**																							
Stage I	318 (2.3)	0 (0.0)	0 (0.0)	0 (0.0)	0 (0.0)	0 (0.0)	0 (0.0)	31 (10.1)	0 (0.0)	0 (0.0)	0 (0.0)	1 (2.0)	0 (0.0)	0 (0.0)	0 (0.0)	0 (0.0)	161 (39.0)	0 (0.0)	0 (0.0)	0 (0.0)	0 (0.0)	0 (0.0)	125 (13.0)
Stage II	725 (5.3)	0 (0.0)	0 (0.0)	0 (0.0)	0 (0.0)	0 (0.0)	0 (0.0)	127 (41.2)	0 (0.0)	0 (0.0)	0 (0.0)	37 (72.5)	0 (0.0)	0 (0.0)	0 (0.0)	0 (0.0)	178 (43.1)	0 (0.0)	0 (0.0)	0 (0.0)	0 (0.0)	0 (0.0)	383 (39.7)
Stage III	280 (2.0)	0 (0.0)	0 (0.0)	0 (0.0)	0 (0.0)	0 (0.0)	0 (0.0)	63 (20.5)	0 (0.0)	0 (0.0)	0 (0.0)	13 (25.5)	0 (0.0)	0 (0.0)	0 (0.0)	0 (0.0)	57 (13.8)	0 (0.0)	0 (0.0)	0 (0.0)	0 (0.0)	0 (0.0)	147 (15.2)
Stage IV	32 (0.2)	0 (0.0)	0 (0.0)	0 (0.0)	0 (0.0)	0 (0.0)	0 (0.0)	6 (1.9)	0 (0.0)	0 (0.0)	0 (0.0)	0 (0.0)	0 (0.0)	0 (0.0)	0 (0.0)	0 (0.0)	16 (3.9)	0 (0.0)	0 (0.0)	0 (0.0)	0 (0.0)	0 (0.0)	10 (1.0)
Not available	12376 (90.1)	705 (100.0)	165 (100.0)	118 (100.0)	35 (100.0)	258 (100.0)	168 (100.0)	81 (26.3)	26 (100.0)	67 (100.0)	40 (100.0)	0 (0.0)	72 (100.0)	417 (100.0)	116 (100.0)	48 (100.0)	1 (0.2)	117 (100.0)	1227 (100.0)	137 (100.0)	20 (100.0)	8258 (100.0)	300 (31.1)
**T stage (%)**																							
T1	4923 (35.9)	0 (0.0)	0 (0.0)	0 (0.0)	0 (0.0)	0 (0.0)	0 (0.0)	0 (0.0)	0 (0.0)	0 (0.0)	0 (0.0)	0 (0.0)	0 (0.0)	0 (0.0)	44 (37.9)	5 (10.4)	0 (0.0)	0 (0.0)	782 (63.7)	82 (59.9)	0 (0.0)	3661 (44.3)	349 (36.2)
T1a	3 (0.0)	3 (0.4)	0 (0.0)	0 (0.0)	0 (0.0)	0 (0.0)	0 (0.0)	0 (0.0)	0 (0.0)	0 (0.0)	0 (0.0)	0 (0.0)	0 (0.0)	0 (0.0)	0 (0.0)	0 (0.0)	0 (0.0)	0 (0.0)	0 (0.0)	0 (0.0)	0 (0.0)	0 (0.0)	0 (0.0)
T1b	33 (0.2)	33 (4.7)	0 (0.0)	0 (0.0)	0 (0.0)	0 (0.0)	0 (0.0)	0 (0.0)	0 (0.0)	0 (0.0)	0 (0.0)	0 (0.0)	0 (0.0)	0 (0.0)	0 (0.0)	0 (0.0)	0 (0.0)	0 (0.0)	0 (0.0)	0 (0.0)	0 (0.0)	0 (0.0)	0 (0.0)

T1c	272 (2.0)	272 (38.6)	0 (0.0)	0 (0.0)	0 (0.0)	0 (0.0)	0 (0.0)	0 (0.0)	0 (0.0)	0 (0.0)	0 (0.0)	0 (0.0)	0 (0.0)	0 (0.0)	0 (0.0)	0 (0.0)	0 (0.0)	0 (0.0)	0 (0.0)	0 (0.0)	0 (0.0)	0 (0.0)	0 (0.0)
T2	3156 (23.0)	382 (54.3)	0 (0.0)	0 (0.0)	0 (0.0)	0 (0.0)	0 (0.0)	0 (0.0)	0 (0.0)	0 (0.0)	0 (0.0)	0 (0.0)	0 (0.0)	0 (0.0)	57 (49.1)	13 (27.1)	0 (0.0)	0 (0.0)	407 (33.2)	51 (37.2)	0 (0.0)	1772 (21.5)	474 (49.1)
T3	354 (2.6)	12 (1.7)	0 (0.0)	0 (0.0)	0 (0.0)	0 (0.0)	0 (0.0)	0 (0.0)	0 (0.0)	0 (0.0)	0 (0.0)	0 (0.0)	0 (0.0)	0 (0.0)	7 (6.0)	21 (43.8)	0 (0.0)	0 (0.0)	29 (2.4)	3 (2.2)	0 (0.0)	175 (2.1)	107 (11.1)
T4	83 (0.6)	0 (0.0)	0 (0.0)	0 (0.0)	0 (0.0)	0 (0.0)	0 (0.0)	0 (0.0)	0 (0.0)	0 (0.0)	0 (0.0)	0 (0.0)	0 (0.0)	0 (0.0)	0 (0.0)	9 (18.8)	0 (0.0)	0 (0.0)	9 (0.7)	1 (0.7)	0 (0.0)	39 (0.5)	25 (2.6)
Tx	2 (0.0)	2 (0.3)	0 (0.0)	0 (0.0)	0 (0.0)	0 (0.0)	0 (0.0)	0 (0.0)	0 (0.0)	0 (0.0)	0 (0.0)	0 (0.0)	0 (0.0)	0 (0.0)	0 (0.0)	0 (0.0)	0 (0.0)	0 (0.0)	0 (0.0)	0 (0.0)	0 (0.0)	0 (0.0)	0 (0.0)
TX	2 (0.0)	0 (0.0)	0 (0.0)	0 (0.0)	0 (0.0)	0 (0.0)	0 (0.0)	0 (0.0)	0 (0.0)	0 (0.0)	0 (0.0)	0 (0.0)	0 (0.0)	0 (0.0)	0 (0.0)	0 (0.0)	0 (0.0)	0 (0.0)	0 (0.0)	0 (0.0)	0 (0.0)	0 (0.0)	2 (0.2)
Not available	4902 (35.7)	0 (0.0)	165 (100.0)	118 (100.0)	35 (100.0)	258 (100.0)	168 (100.0)	308 (100.0)	26 (100.0)	67 (100.0)	40 (100.0)	51 (100.0)	72 (100.0)	417 (100.0)	8 (6.9)	0 (0.0)	413 (100.0)	117 (100.0)	0 (0.0)	0 (0.0)	20 (100.0)	2611 (31.6)	8 (0.8)
**N stage (%)**																							
N0	5581 (40.6)	348 (49.4)	0 (0.0)	0 (0.0)	0 (0.0)	0 (0.0)	0 (0.0)	0 (0.0)	0 (0.0)	0 (0.0)	0 (0.0)	29 (56.9)	0 (0.0)	0 (0.0)	50 (43.1)	6 (12.5)	0 (0.0)	0 (0.0)	641 (52.2)	63 (46.0)	0 (0.0)	4444 (53.8)	0 (0.0)
N1	2732 (19.9)	213 (30.2)	0 (0.0)	0 (0.0)	0 (0.0)	0 (0.0)	0 (0.0)	0 (0.0)	0 (0.0)	0 (0.0)	0 (0.0)	0 (0.0)	0 (0.0)	0 (0.0)	40 (34.5)	25 (52.1)	0 (0.0)	0 (0.0)	380 (31.0)	0 (0.0)	0 (0.0)	2074 (25.1)	0 (0.0)
N2	969 (7.1)	85 (12.1)	0 (0.0)	0 (0.0)	0 (0.0)	0 (0.0)	0 (0.0)	0 (0.0)	0 (0.0)	0 (0.0)	0 (0.0)	0 (0.0)	0 (0.0)	0 (0.0)	20 (17.2)	17 (35.4)	0 (0.0)	0 (0.0)	116 (9.5)	0 (0.0)	0 (0.0)	731 (8.9)	0 (0.0)
N3	124 (0.9)	59 (8.4)	0 (0.0)	0 (0.0)	0 (0.0)	0 (0.0)	0 (0.0)	0 (0.0)	0 (0.0)	0 (0.0)	0 (0.0)	0 (0.0)	0 (0.0)	0 (0.0)	6 (5.2)	0 (0.0)	0 (0.0)	0 (0.0)	59 (4.8)	0 (0.0)	0 (0.0)	0 (0.0)	0 (0.0)
Not available	4325 (31.5)	0 (0.0)	165 (100.0)	118 (100.0)	35 (100.0)	258 (100.0)	168 (100.0)	308 (100.0)	26 (100.0)	67 (100.0)	40 (100.0)	22 (43.1)	72 (100.0)	417 (100.0)	0 (0.0)	0 (0.0)	413 (100.0)	117 (100.0)	31 (2.5)	74 (54.0)	20 (100.0)	1009 (12.2)	965 (100.0)
**M stage (%)**																							
M0	134 (1.0)	0 (0.0)	0 (0.0)	0 (0.0)	0 (0.0)	0 (0.0)	0 (0.0)	0 (0.0)	0 (0.0)	0 (0.0)	0 (0.0)	0 (0.0)	0 (0.0)	0 (0.0)	90 (77.6)	44 (91.7)	0 (0.0)	0 (0.0)	0 (0.0)	0 (0.0)	0 (0.0)	0 (0.0)	0 (0.0)
M1	28 (0.2)	0 (0.0)	0 (0.0)	0 (0.0)	0 (0.0)	0 (0.0)	0 (0.0)	0 (0.0)	0 (0.0)	0 (0.0)	0 (0.0)	0 (0.0)	0 (0.0)	0 (0.0)	26 (22.4)	2 (4.2)	0 (0.0)	0 (0.0)	0 (0.0)	0 (0.0)	0 (0.0)	0 (0.0)	0 (0.0)
Not available	13569 (98.8)	705 (100.0)	165 (100.0)	118 (100.0)	35 (100.0)	258 (100.0)	168 (100.0)	308 (100.0)	26 (100.0)	67 (100.0)	40 (100.0)	51 (100.0)	72 (100.0)	417 (100.0)	0 (0.0)	2 (4.2)	413 (100.0)	117 (100.0)	1227 (100.0)	137 (100.0)	20 (100.0)	8258 (100.0)	965 (100.0)
**ER status (%)**																							
Negative	2235 (16.3)	175 (24.8)	0 (0.0)	0 (0.0)	0 (0.0)	104 (40.3)	77 (45.8)	78 (25.3)	0 (0.0)	19 (28.4)	17 (42.5)	27 (52.9)	24 (33.3)	0 (0.0)	0 (0.0)	23 (47.9)	0 (0.0)	0 (0.0)	298 (24.3)	25 (18.2)	0 (0.0)	1203 (14.6)	165 (17.1)
Positive	9108 (66.3)	530 (75.2)	0 (0.0)	0 (0.0)	0 (0.0)	154 (59.7)	91 (54.2)	142 (46.1)	0 (0.0)	42 (62.7)	18 (45.0)	24 (47.1)	48 (66.7)	0 (0.0)	0 (0.0)	25 (52.1)	0 (0.0)	0 (0.0)	894 (72.9)	111 (81.0)	0 (0.0)	6524 (79.0)	505 (52.3)
Not available	2388 (17.4)	0 (0.0)	165 (100.0)	118 (100.0)	35 (100.0)	0 (0.0)	0 (0.0)	88 (28.6)	26 (100.0)	6 (9.0)	5 (12.5)	0 (0.0)	0 (0.0)	417 (100.0)	116 (100.0)	0 (0.0)	413 (100.0)	117 (100.0)	35 (2.9)	1 (0.7)	20 (100.0)	531 (6.4)	295 (30.6)
**PR status (%)**																							
Negative	3267 (23.8)	249 (35.3)	0 (0.0)	0 (0.0)	0 (0.0)	147 (57.0)	93 (55.4)	103 (33.4)	0 (0.0)	26 (38.8)	26 (65.0)	0 (0.0)	0 (0.0)	0 (0.0)	0 (0.0)	25 (52.1)	0 (0.0)	0 (0.0)	0 (0.0)	57 (41.6)	0 (0.0)	2317 (28.1)	224 (23.2)
Positive	6752 (49.2)	456 (64.7)	0 (0.0)	0 (0.0)	0 (0.0)	111 (43.0)	75 (44.6)	115 (37.3)	0 (0.0)	35 (52.2)	9 (22.5)	0 (0.0)	0 (0.0)	0 (0.0)	0 (0.0)	23 (47.9)	0 (0.0)	0 (0.0)	0 (0.0)	79 (57.7)	0 (0.0)	5406 (65.5)	443 (45.9)
Not available	3712 (27.0)	0 (0.0)	165 (100.0)	118 (100.0)	35 (100.0)	0 (0.0)	0 (0.0)	90 (29.2)	26 (100.0)	6 (9.0)	5 (12.5)	51 (100.0)	72 (100.0)	417 (100.0)	116 (100.0)	0 (0.0)	413 (100.0)	117 (100.0)	1227 (100.0)	1 (0.7)	20 (100.0)	535 (6.5)	298 (30.9)
**HER2 status (%)**																							
Negative	9365 (68.2)	535 (75.9)	0 (0.0)	0 (0.0)	0 (0.0)	206 (79.8)	145 (86.3)	148 (48.1)	0 (0.0)	29 (43.3)	22 (55.0)	0 (0.0)	41 (56.9)	0 (0.0)	66 (56.9)	34 (70.8)	0 (0.0)	0 (0.0)	902 (73.5)	118 (86.1)	0 (0.0)	6551 (79.3)	568 (58.9)
Positive	1689 (12.3)	170 (24.1)	0 (0.0)	0 (0.0)	0 (0.0)	52 (20.2)	23 (13.7)	55 (17.9)	0 (0.0)	32 (47.8)	13 (32.5)	0 (0.0)	31 (43.1)	0 (0.0)	50 (43.1)	12 (25.0)	0 (0.0)	0 (0.0)	116 (9.5)	19 (13.9)	0 (0.0)	1023 (12.4)	93 (9.6)
Not available	2677 (19.5)	0 (0.0)	165 (100.0)	118 (100.0)	35 (100.0)	0 (0.0)	0 (0.0)	105 (34.1)	26 (100.0)	6 (9.0)	5 (12.5)	51 (100.0)	0 (0.0)	417 (100.0)	0 (0.0)	2 (4.2)	413 (100.0)	117 (100.0)	209 (17.0)	0 (0.0)	20 (100.0)	684 (8.3)	304 (31.5)
**TNBC (%)**																							
TNBC	1287 (9.3)	100 (14.2)	0 (0.0)	0 (0.0)	0 (0.0)	65 (25.2)	57 (33.9)	47 (15.3)	26 (100.0)	3 (4.5)	11 (27.5)	14 (27.5)	0 (0.0)	0 (0.0)	0 (0.0)	13 (27.1)	0 (0.0)	0 (0.0)	0 (0.0)	16 (11.7)	0 (0.0)	820 (9.9)	115 (11.9)
non-TNBC	8587 (62.5)	605 (85.8)	0 (0.0)	0 (0.0)	0 (0.0)	193 (74.8)	111 (66.1)	153 (49.7)	0 (0.0)	58 (86.6)	24 (60.0)	35 (68.6)	0 (0.0)	0 (0.0)	0 (0.0)	33 (68.8)	0 (0.0)	0 (0.0)	0 (0.0)	120 (87.6)	0 (0.0)	6747 (81.7)	508 (52.6)
Not available	3857 (28.1)	0 (0.0)	165 (100.0)	118 (100.0)	35 (100.0)	0 (0.0)	0 (0.0)	108 (35.1)	0 (0.0)	6 (9.0)	5 (12.5)	2 (3.9)	72 (100.0)	417 (100.0)	116 (100.0)	2 (4.2)	413 (100.0)	117 (100.0)	1227 (100.0)	1 (0.7)	20 (100.0)	691 (8.4)	342 (35.4)
**pCR vs RD (%)**																							
pCR	79 (0.6)	0 (0.0)	0 (0.0)	0 (0.0)	0 (0.0)	55 (21.3)	24 (14.3)	0 (0.0)	0 (0.0)	0 (0.0)	0 (0.0)	0 (0.0)	0 (0.0)	0 (0.0)	0 (0.0)	0 (0.0)	0 (0.0)	0 (0.0)	0 (0.0)	0 (0.0)	0 (0.0)	0 (0.0)	0 (0.0)
RD	347 (2.5)	0 (0.0)	0 (0.0)	0 (0.0)	0 (0.0)	203 (78.7)	144 (85.7)	0 (0.0)	0 (0.0)	0 (0.0)	0 (0.0)	0 (0.0)	0 (0.0)	0 (0.0)	0 (0.0)	0 (0.0)	0 (0.0)	0 (0.0)	0 (0.0)	0 (0.0)	0 (0.0)	0 (0.0)	0 (0.0)
Not available	12375 (90.1)	0 (0.0)	165 (100.0)	0 (0.0)	35 (100.0)	0 (0.0)	0 (0.0)	308 (100.0)	26 (100.0)	0 (0.0)	0 (0.0)	51 (100.0)	72 (100.0)	417 (100.0)	116 (100.0)	48 (100.0)	413 (100.0)	117 (100.0)	1227 (100.0)	137 (100.0)	20 (100.0)	8258 (100.0)	965 (100.0)

**PAM50 subtype (%)**																							
Basal	1999 (14.6)	110 (15.6)	37 (22.4)	21 (17.8)	8 (22.9)	65 (25.2)	46 (27.4)	74 (24.0)	12 (46.2)	10 (14.9)	8 (20.0)	15 (29.4)	10 (13.9)	59 (14.1)	35 (30.2)	11 (22.9)	75 (18.2)	14 (12.0)	194 (15.8)	15 (10.9)	7 (35.0)	983 (11.9)	190 (19.7)
Her2	1908 (13.9)	157 (22.3)	37 (22.4)	13 (11.0)	8 (22.9)	37 (14.3)	38 (22.6)	53 (17.2)	4 (15.4)	11 (16.4)	12 (30.0)	12 (23.5)	14 (19.4)	66 (15.8)	32 (27.6)	9 (18.8)	45 (10.9)	19 (16.2)	154 (12.6)	18 (13.1)	4 (20.0)	1054 (12.8)	111 (11.5)
LumA	3980 (29.0)	169 (24.0)	24 (14.5)	37 (31.4)	10 (28.6)	78 (30.2)	25 (14.9)	75 (24.4)	6 (23.1)	19 (28.4)	10 (25.0)	14 (27.5)	18 (25.0)	111 (26.6)	29 (25.0)	14 (29.2)	127 (30.8)	32 (27.4)	375 (30.6)	47 (34.3)	5 (25.0)	2459 (29.8)	296 (30.7)
LumB	5844 (42.6)	269 (38.2)	67 (40.6)	47 (39.8)	9 (25.7)	78 (30.2)	59 (35.1)	106 (34.4)	4 (15.4)	27 (40.3)	10 (25.0)	10 (19.6)	30 (41.7)	181 (43.4)	20 (17.2)	14 (29.2)	166 (40.2)	52 (44.4)	504 (41.1)	57 (41.6)	4 (20.0)	3762 (45.6)	368 (38.1)
**brca1 mut (mean (SD))**	0.03 (0.17)	NaN (NA)	0.03 (0.17)	NaN (NA)	NaN (NA)	NaN (NA)	NaN (NA)	NaN (NA)	NaN (NA)	NaN (NA)	NaN (NA)	NaN (NA)	NaN (NA)	NaN (NA)	NaN (NA)	NaN (NA)	NaN (NA)	NaN (NA)	NaN (NA)	NaN (NA)	NaN (NA)	NaN (NA)	NaN (NA)
**brca2 mut (mean (SD))**	0.07 (0.26)	NaN (NA)	0.07 (0.26)	NaN (NA)	NaN (NA)	NaN (NA)	NaN (NA)	NaN (NA)	NaN (NA)	NaN (NA)	NaN (NA)	NaN (NA)	NaN (NA)	NaN (NA)	NaN (NA)	NaN (NA)	NaN (NA)	NaN (NA)	NaN (NA)	NaN (NA)	NaN (NA)	NaN (NA)	NaN (NA)
**Grade (%)**																							
G1	1214 (8.8)	6 (0.9)	0 (0.0)	0 (0.0)	5 (14.3)	0 (0.0)	12 (7.1)	1 (0.3)	0 (0.0)	3 (4.5)	4 (10.0)	5 (9.8)	0 (0.0)	0 (0.0)	0 (0.0)	1 (2.1)	86 (20.8)	0 (0.0)	0 (0.0)	14 (10.2)	0 (0.0)	1077 (13.0)	0 (0.0)
G2	4181 (30.4)	320 (45.4)	0 (0.0)	0 (0.0)	18 (51.4)	0 (0.0)	58 (34.5)	4 (1.3)	0 (0.0)	35 (52.2)	15 (37.5)	17 (33.3)	0 (0.0)	0 (0.0)	0 (0.0)	19 (39.6)	152 (36.8)	0 (0.0)	0 (0.0)	44 (32.1)	0 (0.0)	3499 (42.4)	0 (0.0)
G3	3309 (24.1)	314 (44.5)	0 (0.0)	0 (0.0)	12 (34.3)	0 (0.0)	68 (40.5)	13 (4.2)	0 (0.0)	24 (35.8)	16 (40.0)	24 (47.1)	0 (0.0)	0 (0.0)	0 (0.0)	26 (54.2)	173 (41.9)	0 (0.0)	0 (0.0)	17 (12.4)	0 (0.0)	2622 (31.8)	0 (0.0)
Not available	5027 (36.7)	65 (9.2)	165 (100.0)	118 (100.0)	0 (0.0)	258 (100.0)	30 (17.9)	290 (94.2)	26 (100.0)	5 (7.5)	5 (12.5)	5 (9.8)	72 (100.0)	417 (100.0)	116 (100.0)	2 (4.2)	2 (0.5)	117 (100.0)	1227 (100.0)	62 (45.3)	20 (100.0)	1060 (12.8)	965 (100.0)
**CIBERSORTx_B_cells_naive (median [IQR])**	0.06 [0.03, 0.10]	0.09 [0.04, 0.16]	0.04 [0.02, 0.08]	0.13 [0.07, 0.17]	0.28 [0.24, 0.35]	0.12 [0.07, 0.17]	0.00 [0.00, 0.02]	0.07 [0.03, 0.12]	0.13 [0.05, 0.24]	0.04 [0.01, 0.09]	0.12 [0.09, 0.14]	0.07 [0.05, 0.10]	0.08 [0.05, 0.11]	0.09 [0.05, 0.11]	0.00 [0.00, 0.03]	0.06 [0.00, 0.13]	0.02 [0.00, 0.04]	0.10 [0.07, 0.13]	NA [NA, NA]	0.04 [0.02, 0.06]	0.29 [0.13, 0.52]	0.05 [0.03, 0.08]	0.14 [0.09, 0.22]
**CIBERSORTx_B_cells_memory (median [IQR])**	0.00 [0.00, 0.02]	0.02 [0.00, 0.07]	0.00 [0.00, 0.02]	0.00 [0.00, 0.07]	0.00 [0.00, 0.01]	0.02 [0.00, 0.08]	0.14 [0.10, 0.17]	0.01 [0.00, 0.06]	0.00 [0.00, 0.06]	0.00 [0.00, 0.02]	0.00 [0.00, 0.04]	0.09 [0.06, 0.12]	0.00 [0.00, 0.00]	0.00 [0.00, 0.02]	0.00 [0.00, 0.02]	0.11 [0.03, 0.22]	0.02 [0.00, 0.05]	0.03 [0.00, 0.06]	NA [NA, NA]	0.00 [0.00, 0.00]	0.04 [0.00, 0.15]	0.00 [0.00, 0.01]	0.00 [0.00, 0.00]
**CIBERSORTx_Plasma_cells (median [IQR])**	0.10 [0.04, 0.19]	0.12 [0.08, 0.20]	0.01 [0.00, 0.03]	0.17 [0.08, 0.25]	0.00 [0.00, 0.00]	0.12 [0.06, 0.20]	0.10 [0.05, 0.17]	0.31 [0.18, 0.41]	0.06 [0.03, 0.10]	0.64 [0.51, 0.91]	0.12 [0.08, 0.15]	0.14 [0.12, 0.18]	0.38 [0.27, 0.46]	0.20 [0.14, 0.30]	1.29 [0.92, 1.78]	0.65 [0.40, 0.75]	0.35 [0.23, 0.48]	0.13 [0.09, 0.18]	NA [NA, NA]	0.07 [0.05, 0.11]	0.00 [0.00, 0.01]	0.08 [0.04, 0.16]	0.06 [0.02, 0.12]
**CIBERSORTx_T_cells_CD8 (median [IQR])**	0.12 [0.05, 0.20]	0.10 [0.04, 0.20]	0.21 [0.13, 0.36]	0.02 [0.00, 0.09]	0.00 [0.00, 0.00]	0.07 [0.00, 0.15]	0.12 [0.08, 0.16]	0.00 [0.00, 0.02]	0.17 [0.10, 0.31]	0.01 [0.00, 0.06]	0.25 [0.21, 0.29]	0.33 [0.28, 0.39]	0.07 [0.03, 0.12]	0.02 [0.00, 0.05]	0.00 [0.00, 0.05]	0.41 [0.24, 0.61]	0.00 [0.00, 0.03]	0.32 [0.27, 0.38]	NA [NA, NA]	0.02 [0.01, 0.05]	0.03 [0.00, 0.10]	0.14 [0.08, 0.21]	0.09 [0.03, 0.20]
**CIBERSORTx_T_cells_CD4_naive (median [IQR])**	0.00 [0.00, 0.00]	0.00 [0.00, 0.00]	0.00 [0.00, 0.00]	0.07 [0.00, 0.14]	0.12 [0.08, 0.14]	0.10 [0.02, 0.19]	0.14 [0.08, 0.16]	0.01 [0.00, 0.08]	0.00 [0.00, 0.00]	0.00 [0.00, 0.00]	0.00 [0.00, 0.00]	0.00 [0.00, 0.00]	0.00 [0.00, 0.00]	0.00 [0.00, 0.00]	0.00 [0.00, 0.00]	0.00 [0.00, 0.07]	0.06 [0.01, 0.12]	0.00 [0.00, 0.00]	NA [NA, NA]	0.00 [0.00, 0.01]	0.28 [0.00, 0.41]	0.00 [0.00, 0.00]	0.00 [0.00, 0.00]
**CIBERSORTx_T_cells_CD4_memory_resting (median [IQR])**	0.33 [0.21, 0.43]	0.52 [0.39, 0.72]	0.41 [0.30, 0.60]	0.09 [0.02, 0.15]	0.07 [0.02, 0.13]	0.02 [0.00, 0.09]	0.00 [0.00, 0.00]	0.16 [0.09, 0.25]	0.50 [0.29, 0.60]	0.48 [0.34, 0.58]	0.23 [0.13, 0.32]	0.03 [0.00, 0.08]	0.11 [0.02, 0.15]	0.00 [0.00, 0.00]	0.25 [0.13, 0.39]	0.04 [0.00, 0.16]	0.00 [0.00, 0.06]	0.23 [0.15, 0.31]	NA [NA, NA]	0.30 [0.23, 0.38]	0.63 [0.40, 0.98]	0.35 [0.28, 0.44]	0.32 [0.17, 0.49]
**CIBERSORTx_T_cells_CD4_memory_activated (median [IQR])**	0.00 [0.00, 0.03]	0.00 [0.00, 0.01]	0.00 [0.00, 0.04]	0.00 [0.00, 0.03]	0.08 [0.05, 0.11]	0.02 [0.00, 0.06]	0.15 [0.09, 0.18]	0.05 [0.01, 0.10]	0.00 [0.00, 0.02]	0.02 [0.00, 0.07]	0.02 [0.00, 0.06]	0.02 [0.00, 0.04]	0.02 [0.00, 0.07]	0.00 [0.00, 0.01]	0.04 [0.00, 0.17]	0.11 [0.02, 0.26]	0.00 [0.00, 0.00]	0.02 [0.00, 0.04]	NA [NA, NA]	0.04 [0.01, 0.08]	0.00 [0.00, 0.07]	0.00 [0.00, 0.02]	0.00 [0.00, 0.02]
**CIBERSORTx_T_cells_follicular_helper (median [IQR])**	0.06 [0.03, 0.11]	0.05 [0.01, 0.10]	0.15 [0.11, 0.22]	0.12 [0.09, 0.17]	0.07 [0.05, 0.10]	0.12 [0.07, 0.18]	0.02 [0.01, 0.05]	0.10 [0.07, 0.14]	0.21 [0.14, 0.31]	0.18 [0.13, 0.23]	0.08 [0.04, 0.11]	0.08 [0.06, 0.09]	0.16 [0.12, 0.20]	0.46 [0.39, 0.54]	0.30 [0.22, 0.46]	0.06 [0.01, 0.14]	0.25 [0.20, 0.30]	0.02 [0.00, 0.05]	NA [NA, NA]	0.03 [0.01, 0.06]	0.05 [0.00, 0.22]	0.05 [0.02, 0.08]	0.17 [0.11, 0.28]
**CIBERSORTx_T_cells_regulatory_(Tregs) (median [IQR])**	0.03 [0.00, 0.08]	0.00 [0.00, 0.00]	0.02 [0.00, 0.05]	0.07 [0.04, 0.10]	0.02 [0.00, 0.03]	0.11 [0.07, 0.15]	0.05 [0.02, 0.07]	0.01 [0.00, 0.04]	0.04 [0.00, 0.10]	0.00 [0.00, 0.00]	0.01 [0.00, 0.04]	0.06 [0.03, 0.08]	0.02 [0.00, 0.05]	0.02 [0.00, 0.05]	0.00 [0.00, 0.03]	0.07 [0.03, 0.15]	0.06 [0.03, 0.09]	0.01 [0.00, 0.05]	NA [NA, NA]	0.00 [0.00, 0.02]	0.02 [0.00, 0.08]	0.04 [0.00, 0.08]	0.07 [0.03, 0.13]
**CIBERSORTx_T_cells_gamma_delta (median [IQR])**	0.00 [0.00, 0.04]	0.00 [0.00, 0.00]	0.01 [0.00, 0.06]	0.11 [0.02, 0.16]	0.21 [0.17, 0.25]	0.12 [0.06, 0.17]	0.01 [0.00, 0.03]	0.18 [0.13, 0.25]	0.00 [0.00, 0.00]	0.37 [0.25, 0.49]	0.00 [0.00, 0.00]	0.00 [0.00, 0.00]	0.17 [0.11, 0.26]	0.05 [0.01, 0.11]	0.27 [0.16, 0.41]	0.04 [0.00, 0.15]	0.19 [0.11, 0.28]	0.00 [0.00, 0.00]	NA [NA, NA]	0.06 [0.02, 0.09]	0.00 [0.00, 0.00]	0.00 [0.00, 0.01]	0.00 [0.00, 0.00]
**CIBERSORTx_NK_cells_resting (median [IQR])**	0.00 [0.00, 0.04]	0.08 [0.04, 0.13]	0.00 [0.00, 0.00]	0.04 [0.00, 0.09]	0.04 [0.01, 0.07]	0.09 [0.02, 0.17]	0.20 [0.14, 0.25]	0.01 [0.00, 0.05]	0.05 [0.00, 0.10]	0.00 [0.00, 0.00]	0.09 [0.07, 0.12]	0.09 [0.07, 0.13]	0.01 [0.00, 0.05]	0.02 [0.00, 0.06]	0.00 [0.00, 0.00]	0.00 [0.00, 0.00]	0.00 [0.00, 0.00]	0.08 [0.05, 0.09]	NA [NA, NA]	0.12 [0.09, 0.15]	0.14 [0.09, 0.30]	0.00 [0.00, 0.02]	0.05 [0.02, 0.09]
**CIBERSORTx_NK_cells_activated (median [IQR])**	0.04 [0.00, 0.08]	0.00 [0.00, 0.02]	0.05 [0.03, 0.09]	0.00 [0.00, 0.03]	0.00 [0.00, 0.00]	0.00 [0.00, 0.03]	0.00 [0.00, 0.00]	0.00 [0.00, 0.02]	0.04 [0.00, 0.10]	0.02 [0.00, 0.08]	0.00 [0.00, 0.01]	0.00 [0.00, 0.00]	0.00 [0.00, 0.03]	0.04 [0.01, 0.08]	0.04 [0.00, 0.12]	0.44 [0.33, 0.55]	0.13 [0.10, 0.18]	0.00 [0.00, 0.00]	NA [NA, NA]	0.00 [0.00, 0.00]	0.00 [0.00, 0.04]	0.05 [0.02, 0.08]	0.00 [0.00, 0.02]
**CIBERSORTx_Monocytes (median [IQR])**	0.04 [0.01, 0.07]	0.04 [0.01, 0.08]	0.04 [0.02, 0.08]	0.01 [0.00, 0.04]	0.00 [0.00, 0.00]	0.06 [0.01, 0.12]	0.03 [0.02, 0.05]	0.00 [0.00, 0.01]	0.02 [0.00, 0.04]	0.00 [0.00, 0.00]	0.11 [0.07, 0.13]	0.07 [0.04, 0.10]	0.00 [0.00, 0.00]	0.00 [0.00, 0.02]	0.00 [0.00, 0.00]	0.00 [0.00, 0.02]	0.00 [0.00, 0.01]	0.12 [0.08, 0.15]	NA [NA, NA]	0.03 [0.01, 0.05]	0.04 [0.02, 0.06]	0.05 [0.02, 0.08]	0.01 [0.00, 0.04]
**CIBERSORTx_Macrophages_M0 (median [IQR])**	0.19 [0.07, 0.35]	0.31 [0.17, 0.46]	0.11 [0.02, 0.22]	0.30 [0.17, 0.41]	0.05 [0.03, 0.07]	0.28 [0.17, 0.41]	0.04 [0.00, 0.13]	0.29 [0.18, 0.43]	0.26 [0.15, 0.44]	0.38 [0.20, 0.57]	0.15 [0.10, 0.24]	0.16 [0.12, 0.27]	0.18 [0.11, 0.27]	0.29 [0.20, 0.46]	0.74 [0.44, 1.16]	0.63 [0.46, 0.85]	0.29 [0.18, 0.45]	0.12 [0.06, 0.20]	NA [NA, NA]	0.25 [0.15, 0.35]	0.21 [0.15, 0.38]	0.14 [0.04, 0.30]	0.38 [0.18, 0.61]
**CIBERSORTx_Macrophages_M1 (median [IQR])**	0.13 [0.09, 0.18]	0.10 [0.06, 0.17]	0.24 [0.15, 0.34]	0.20 [0.17, 0.27]	0.18 [0.16, 0.20]	0.11 [0.07, 0.16]	0.04 [0.03, 0.07]	0.21 [0.16, 0.25]	0.10 [0.06, 0.14]	0.29 [0.20, 0.41]	0.07 [0.04, 0.13]	0.07 [0.05, 0.11]	0.26 [0.19, 0.31]	0.16 [0.10, 0.23]	0.61 [0.39, 0.83]	0.35 [0.28, 0.42]	0.18 [0.13, 0.23]	0.07 [0.04, 0.11]	NA [NA, NA]	0.20 [0.15, 0.24]	0.05 [0.01, 0.13]	0.12 [0.09, 0.16]	0.13 [0.08, 0.24]
**CIBERSORTx_Macrophages_M2 (median [IQR])**	0.51 [0.38, 0.66]	0.46 [0.36, 0.57]	0.66 [0.54, 0.80]	0.25 [0.17, 0.30]	0.11 [0.07, 0.14]	0.13 [0.08, 0.19]	0.08 [0.05, 0.11]	0.37 [0.30, 0.46]	0.30 [0.20, 0.37]	0.69 [0.58, 0.91]	0.30 [0.25, 0.35]	0.20 [0.18, 0.26]	0.44 [0.34, 0.50]	0.28 [0.21, 0.34]	0.73 [0.54, 0.95]	0.74 [0.62, 0.84]	0.32 [0.25, 0.41]	0.19 [0.16, 0.24]	NA [NA, NA]	0.29 [0.24, 0.36]	0.08 [0.01, 0.15]	0.56 [0.45, 0.69]	0.55 [0.43, 0.72]
**CIBERSORTx_Dendritic_cells_resting (median [IQR])**	0.02 [0.00, 0.05]	0.00 [0.00, 0.00]	0.03 [0.02, 0.05]	0.08 [0.06, 0.10]	0.17 [0.14, 0.18]	0.08 [0.06, 0.12]	0.12 [0.10, 0.14]	0.05 [0.02, 0.08]	0.00 [0.00, 0.00]	0.03 [0.02, 0.07]	0.00 [0.00, 0.00]	0.00 [0.00, 0.00]	0.05 [0.03, 0.06]	0.05 [0.03, 0.08]	0.05 [0.01, 0.10]	0.02 [0.00, 0.07]	0.03 [0.01, 0.06]	0.00 [0.00, 0.00]	NA [NA, NA]	0.01 [0.00, 0.02]	0.00 [0.00, 0.04]	0.02 [0.01, 0.05]	0.00 [0.00, 0.01]

CIBERSORTx_Dendritic_cells_activated (median [IQR])	0.00 [0.00, 0.01]	0.02 [0.00, 0.05]	0.00 [0.00, 0.00]	0.00 [0.00, 0.00]	0.00 [0.00, 0.02]	0.00 [0.00, 0.02]	0.02 [0.00, 0.03]	0.00 [0.00, 0.00]	0.03 [0.00, 0.08]	0.00 [0.00, 0.00]	0.05 [0.03, 0.07]	0.03 [0.01, 0.04]	0.00 [0.00, 0.02]	0.03 [0.01, 0.07]	0.00 [0.00, 0.00]	0.00 [0.00, 0.00]	0.01 [0.00, 0.03]	0.03 [0.02, 0.05]	NA [NA, NA]	0.00 [0.00, 0.00]	0.01 [0.00, 0.06]	0.00 [0.00, 0.00]	0.00 [0.00, 0.03]
CIBERSORTx_Mast_cells_resting (median [IQR])	0.15 [0.08, 0.26]	0.10 [0.06, 0.16]	0.11 [0.02, 0.19]	0.17 [0.09, 0.25]	0.10 [0.04, 0.13]	0.03 [0.00, 0.09]	0.00 [0.00, 0.04]	0.14 [0.07, 0.23]	0.04 [0.00, 0.09]	0.15 [0.03, 0.28]	0.10 [0.06, 0.13]	0.10 [0.05, 0.14]	0.13 [0.06, 0.19]	0.00 [0.00, 0.03]	0.00 [0.00, 0.10]	0.19 [0.03, 0.34]	0.13 [0.07, 0.22]	0.10 [0.07, 0.15]	NA [NA, NA]	0.28 [0.21, 0.38]	0.00 [0.00, 0.04]	0.18 [0.11, 0.28]	0.14 [0.08, 0.24]
CIBERSORTx_Mast_cells_activated (median [IQR])	0.00 [0.00, 0.02]	0.00 [0.00, 0.00]	0.01 [0.00, 0.06]	0.07 [0.01, 0.13]	0.12 [0.07, 0.15]	0.08 [0.02, 0.14]	0.08 [0.04, 0.11]	0.03 [0.00, 0.07]	0.00 [0.00, 0.02]	0.12 [0.07, 0.21]	0.00 [0.00, 0.00]	0.00 [0.00, 0.00]	0.03 [0.00, 0.07]	0.11 [0.06, 0.16]	0.12 [0.04, 0.27]	0.00 [0.00, 0.12]	0.00 [0.00, 0.05]	0.00 [0.00, 0.00]	NA [NA, NA]	0.00 [0.00, 0.00]	0.02 [0.00, 0.03]	0.00 [0.00, 0.01]	0.00 [0.00, 0.00]
CIBERSORTx_Eosinophils (median [IQR])	0.00 [0.00, 0.00]	0.00 [0.00, 0.00]	0.00 [0.00, 0.00]	0.00 [0.00, 0.00]	0.04 [0.02, 0.06]	0.00 [0.00, 0.02]	0.00 [0.00, 0.00]	0.00 [0.00, 0.01]	0.00 [0.00, 0.00]	0.00 [0.00, 0.01]	0.00 [0.00, 0.00]	0.00 [0.00, 0.00]	0.00 [0.00, 0.00]	0.00 [0.00, 0.00]	0.00 [0.00, 0.00]	0.00 [0.00, 0.00]	0.00 [0.00, 0.01]	0.00 [0.00, 0.00]	NA [NA, NA]	0.00 [0.00, 0.00]	0.00 [0.00, 0.01]	0.00 [0.00, 0.00]	0.00 [0.00, 0.00]
CIBERSORTx_Neutrophils (median [IQR])	0.02 [0.00, 0.03]	0.00 [0.00, 0.01]	0.00 [0.00, 0.01]	0.00 [0.00, 0.01]	0.02 [0.01, 0.02]	0.13 [0.06, 0.28]	0.06 [0.01, 0.19]	0.01 [0.00, 0.03]	0.01 [0.00, 0.02]	0.00 [0.00, 0.01]	0.00 [0.00, 0.00]	0.00 [0.00, 0.00]	0.01 [0.00, 0.02]	0.01 [0.00, 0.02]	0.04 [0.03, 0.06]	0.00 [0.00, 0.02]	0.01 [0.00, 0.02]	0.00 [0.00, 0.00]	NA [NA, NA]	0.00 [0.00, 0.00]	0.00 [0.00, 0.00]	0.02 [0.01, 0.04]	0.00 [0.00, 0.01]

Abbreviations: ER, estrogen receptor; PR, progesterone receptor; TNBC, triple-negative breast cancer.
